# Emergence of a New [NNN] Pincer Ligand via Si−H Bond Activation and β‐Hydride Abstraction at Tetravalent Cerium

**DOI:** 10.1002/chem.202000625

**Published:** 2020-09-07

**Authors:** Daniel Werner, Uwe Bayer, Dorothea Schädle, Reiner Anwander

**Affiliations:** ^1^ Institut für Anorganische Chemie University of Tübingen (EKUT) Auf der Morgenstelle 18 72076 Tübingen Germany

**Keywords:** cerium, hydride abstraction, lithium, pincer ligands, silicon

## Abstract

The cerium(IV) pyrazolate complexes [Ce(Me_2_pz)_4_]_2_ and [Ce(Me_2_pz)_4_(thf)] initiate β‐hydride abstraction of the bis(dimethylsilyl)amido moiety, to afford a heteroleptic cerium(IV) species containing a dimethylpyrazolyl‐substituted silylamido ligand, namely [Ce(Me_2_pz)_3_(bpsa)] (bpsa=bis((3,5‐dimethylpyrazol‐1‐yl)dimethylsilyl)amido; Me_2_pz =3,5‐dimethylpyrazolato), along with some cerium(III) species. Remarkably, the nucleophilic attack of the pyrazolyl at the silicon atom and concomitant Si−H‐bond cleavage is restricted to the tetravalent cerium oxidation state and appears to proceed via the formation of a transient cerium(IV) hydride, which engages in immediate redox chemistry. When [Ce(Me_2_pz)_4_]_2_ is treated with [Li{N(SiMe_3_)_2_}], that is, in the absence of the SiH functionality, any redox chemistry did not occur. Instead, the ceric ate complex [LiCe_2_(Me_2_pz)_9_] and the stable mixed‐ligand ceric species [Ce(Me_2_pz)_2_{N(SiMe_3_)_2_}_2_] were obtained.

## Graphical abstract

**Putative heteroleptic ceric species** [Ce(Me_2_pz)_*x*_{N(SiHMe_2_)_2_}_4−*x*_] promote the formation of a new monoanionic dipyrazolylamido pincer ligand, while both tetravalent [Ce(Me_2_pz)_2_{N(SiMe_3_)_2_}_2_] and trivalent [Li_2_(thf)_2_Ce(Me_2_pz)_2_{N(SiHMe_2_)_2_}_3_] are isolable and stable.

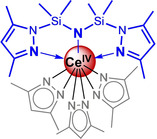

## Introduction

**Figure 1:**
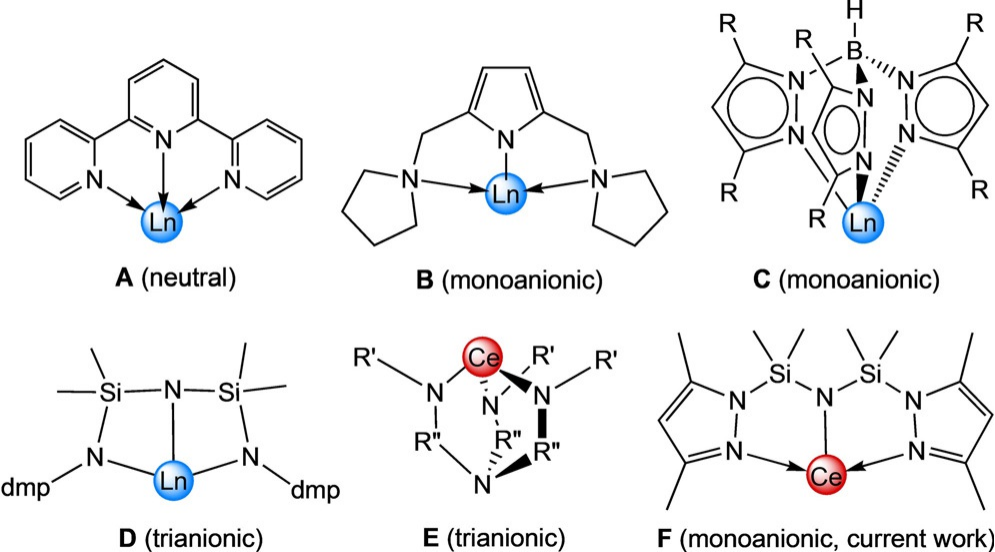
*Top and bottom left*: examples of [NNN] ligands (R=alkyl, aryl; dmp=2,6‐Mes_2_C_6_H_3_, Mes=2,4,6‐Me_3_C_6_H_2_) used in trivalent rare‐earth‐metal chemistry (oxidation state +3 is indicated by a blue sphere). *Bottom middle*: ligands which serve to encapsulate the cerium(IV) ion (e.g. R’=alkyl, TMS; R’’=Et, aryl), and the new monoanionic [NNN] ligand presented in this study (*right*) (oxidation state +4 is indicated by a red sphere).

Tridentate [NNN] ligand scaffolds play a key role for exploring the reactivity and applications of *f*‐block complexes.^1^ For example, neutral terpyridines (Figure 1, **A**, and its derivatives; oxidation state +3 is indicated by a blue sphere) are effective antennae for enhancing the luminescence and subsequent chemical detection properties of rare‐earth‐metal (Ln) complexes.1b, 2 Monoanionic [NNN] pincer ligands (Figure 1, **B**) are successfully utilized for the design of discrete Ln^III^ precatalysts for polymerization reactions.1c, 3, 4 Further, tripodal tris(R,R’‐pyrazolyl)hydroborato scorpionate ligands (Figure 1, **C**),^5^ provide sufficient steric protection to enable the isolation of highly reactive Ln complexes^6^ and to study and scrutinize their chemistry.^7^ Consequentially, the development of new [NNN] derivatives and pincers is a continuous and important aspect of rare‐earth‐metal and indeed inorganic chemistry.^8^ A recent notable addition to this ligand class is the trianionic [N(SiMe_2_Ndmp)_2_] ligand (Figure 1, **D**, dmp=2,6‐Mes_2_C_6_H_3_, Mes=2,4,6‐Me_3_C_6_H_2_), observed in [Ln{N(SiMe_2_Ndmp)_2_}] (Ln=Ce, Pr),^9^ which was obtained by Si−H bond activation, and concomitant formation of dihydrogen gas.

In the realms of tetravalent cerium, its ability to behave as a strong one‐electron oxidant^10^ has attracted considerable attention.^11^ The choice of an appropriate ligand system is delicate since the organic framework has to adopt to both of the cerium(III/IV) redox couple and, in particular, to the smaller ionic radius of cerium(IV) (Ce^III^(CN 6), 1.01 Å vs. Ce^IV^(CN 6), 0.87 Å).^12^ Currently, interest has turned toward mono‐ and multidentate ligand systems complimentarily,^13^ where utilization of chelating moieties (Figure 1, e.g., **E** type; oxidation state +4 is indicated by a red sphere) has undergone a significant revival since their debut in cerium chemistry nearly 20 years ago.^14^ Not only do they prevent the cerium(IV) ion from ligand/redox disproportionation, but the chelate/scaffold routinely leaves an open pocket for an additional reactive ligand.14a, 14b, 14e, 15 Accordingly, the isolation of cerium(IV) complexes featuring terminal Ce=O moieties,14d and even terminal azido ligands could be accomplished.14c Yet, despite the advancement of these polydentate ligands, implementation of simpler monoanionic [NNN] pincer ligands (such as **B**), has remained an underexplored area of ceric chemistry.

Our recent advances in cerium chemistry have also targeted new ligand sets to increase the synthetic value of cerium(IV),^11^, ^16^ with the primary goal to investigate beyond unidirectional redox chemistry. Referring to this, the pyrazolato ligand class has excelled,^16^ with two homoleptic cerium(IV) complexes for both 3,5‐dimethylpyrazolato (Me_2_pz, as [Ce(Me_2_pz)_4_]_2_ (**1**)) and 3,5‐di‐*tert*‐butylpyrazolato (*t*Bu_2_pz, as [Ce(*t*Bu_2_pz)_4_].16a Both complexes were obtained through application of a silylamine elimination protocol utilizing homoleptic cerium(IV) bis(dimethylsilyl)amide [Ce{N(SiHMe_2_)_2_}_4_] as a versatile precursor.^17^, ^18^ However, the synthesis of **1** appeared to involve a second reaction path reducing its yield and forming apparent trivalent products.16a Considering compound **1** is stable across a variety of different solvents (THF, toluene, [D_6_]benzene, and *n*‐hexane),16a and is resistant to thermal decomposition (up to 120 °C in toluene), it may be hypothesized that the second reaction path is related to the bis(dimethylsilyl)amine co‐product. Such reactivity is surprising, and warrants investigation, especially when considering the relevance of this silylamido ligand and other ligands with non‐innocent Si−H moieties, as starting materials.^19^

Herein, we examined the ability of HN(SiHMe_2_)_2_ and monoanionic [N(SiHMe_2_)_2_] to initiate redox chemistry with dimeric **1** and monomeric [Ce(Me_2_pz)_4_(thf)] (**2 a**). The new monoanionic [NNN] pincer ligand [N{SiMe_2_(Me_2_pz)}_2_] is proposed to form via β‐hydride abstraction and a transient ceric hydride. It features a new member of the class of anionic [NNN] pincers which contrasts the tripodal nature of classical [NNN] scorpionates (**C**).^5^ The significance of tetravalent cerium, and the Si−H bond, was supported by comparative reactions with both cerium(III) pyrazolate complexes and the monoanionic [N(SiMe_3_)_2_] ligand.

## Results and Discussion

### Reactivity of [Ce(Me_2_ pz)_4_]_2_ (1) toward HN(SiHMe_2_)_2_

**Scheme 1:**
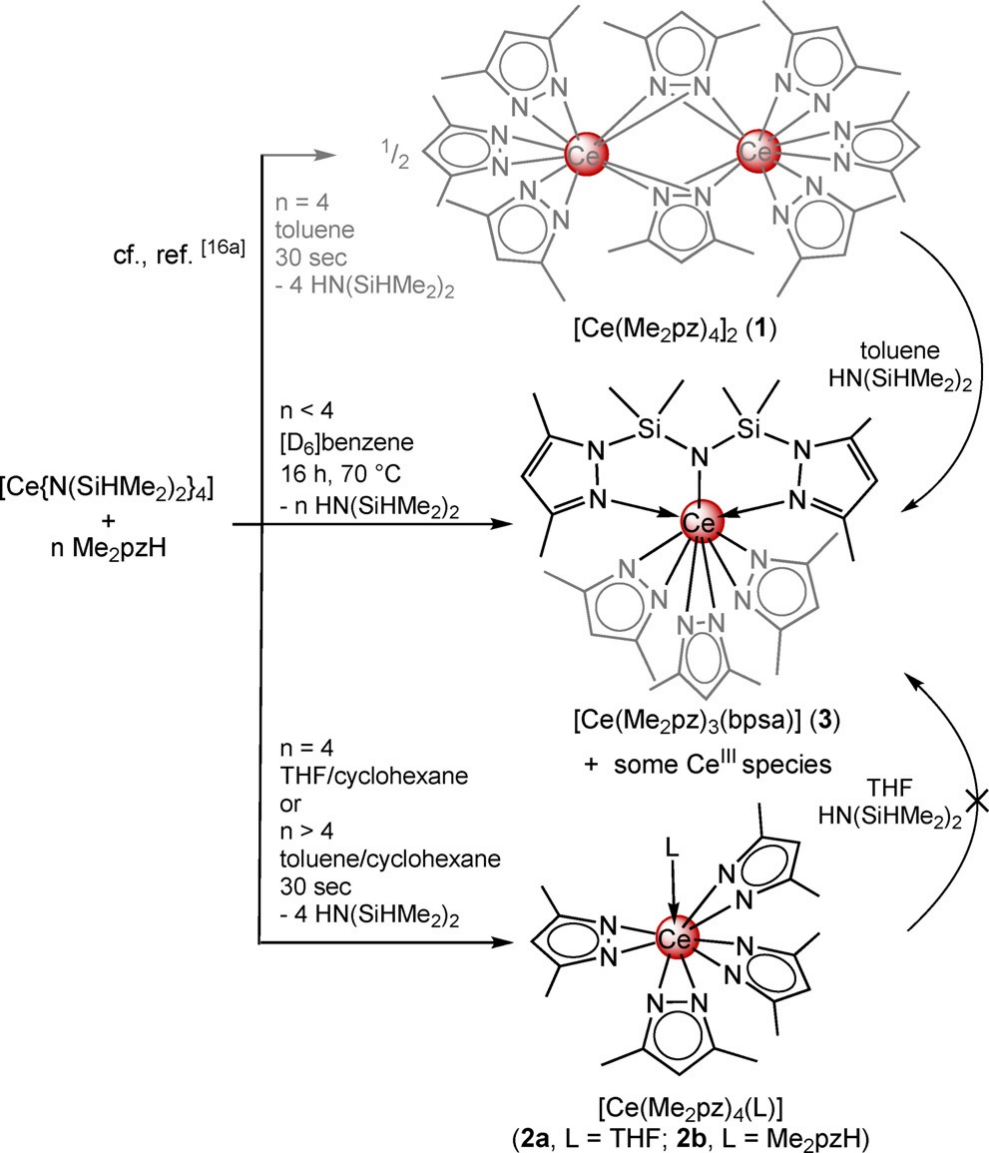
Synthesis of [Ce(Me_2_pz)_4_]_2_ (**1**),16a [Ce(Me_2_pz)_4_(thf)] (**2 a**),^20^ [Ce(Me_2_pz)_4_(Me_2_pzH)] (**2 b**), and cerium complex **3**, coordinated with the [NNN] pincer ligand.

As reported previously, the protonolysis of [Ce{N(SiHMe_2_)_2_}_4_] with four equivalents of Me_2_pzH affords either dimeric [Ce(Me_2_pz)_4_]_2_ (**1**) from toluene (Scheme 1),16a or monomeric [Ce(Me_2_pz)_4_(thf)] (**2 a**) in the presence of THF (Scheme 1).^20^ Monitoring the reaction of [Ce{N(SiHMe_2_)_2_}_4_] with four equivalents of 3,5‐dimethylpyrazole in [D_6_]benzene by ^1^H NMR spectroscopy, however, indicated multiple paramagnetic Ce^III^ and diamagnetic Ce^IV^ species.16a Although these unidentifiable species continued to form over 16 h, the processes dramatically decelerated over several days. The ^1^H NMR spectrum of this reaction mixture was complicated (Figure S1, Supporting Information), but crystallization from *n*‐hexane gave red block crystals of the heteroleptic cerium(IV) pyrazolate complex [Ce(Me_2_pz)_3_(bpsa)] (**3**) bearing a bis(3,5‐dimethylpyrazol‐1‐yl‐dimethylsilyl)amido (bpsa) ligand (Scheme 1). Complex **3** was obtained in low yield, however, it could be repeatedly isolated from the synthesis of either **1** or **2 a** at ambient temperatures. Furthermore, the ^1^H NMR signals attributed to **3** could be identified in the reaction mixture of [Ce{N(SiHMe_2_)_2_}_4_] and Me_2_pzH (Figure S1), indicating **3** is a co‐product of the occurring redox processes.

The formation of **3** supported our initial hypothesis that the [N(SiHMe_2_)_2_] moiety engages in the decomposition process,16a and the bpsa ligand formation involves Si−H bond activation. It should also be noted that when the diisopropylamide complex, [Ce(N*i*Pr_2_)_4_],^21^ was treated with four equivalents of Me_2_pzH in toluene, the reaction mixture changed color to light red within seconds of Me_2_pzH addition, and crystallization from *n*‐hexane gave only crystals of the previously reported trivalent complex [Ce(Me_2_pz)_3_(Me_2_pzH)]_2_,^22^ in moderate yield (34 %).

To further investigate into the parameters of Si−H bond activation, several experiments were performed. First, isolated **1** was treated with half an equivalent of HN(SiHMe_2_)_2_ in [D_6_]benzene. The analysis by NMR spectroscopy indicated a slow redox process. The formation of multiple trivalent cerium species was evident after two days at ambient temperatures, and complete conversion after four months afforded a light‐red solution with **3** as the only diamagnetic species (3 %, Figure S2). During the reaction progress, the following trends were observed: the gradual decrease of the SiH septet (4.72 ppm) and SiMe doublet (0.11 ppm), the broadening of the Me_2_pz singlet (5.85 ppm), and the formation of a variety of new resonances assignable to the new bpsa ligand framework, the most notable being the formation of the SiMe_2_ singlet at 0.13 ppm. Although the reaction between **1** and HN(SiHMe_2_)_2_ is slow at ambient temperatures, its conversion could be improved by heating the reaction mixture to 105 °C (Figure S3) and it appeared complete within 16 hours under optimized conditions. After cooling, colorless block crystals formed and analysis by X‐ray crystallography revealed the formation of trivalent co‐product [Ce^III^_4_(Me_2_pz)_12_(Me_2_pzH)_2_] (**2 c**, Figure S4), as a direct result of a redox process (vide infra). The overall structure of **2 c** is similar to polymeric [La(Me_2_pz)_3_]_∞_,^23^ but the putative polymeric chain is capped by two terminating Me_2_pzH ligands to form the tetrametallic species **2 c**. Such an oligomer formation is interesting considering that the homoleptic species forms a tetrametallic [Ce_4_(Me_2_pz)_12_] cluster.^23^ Nevertheless, it is evident that at ambient temperature release of free amine is presumably not the direct cause of the initial rapid degradation observed during the synthesis of **1**, and it is more likely that the amido ligand engages into Si−H bond activation in the form of transient heteroleptic complexes [Ce(Me_2_pz)_*x*_{N(SiHMe_2_)_2_}_4−*x*_] (*x=*2 (**4 a**) or *x=*3 (**4 b**), cf., Scheme 3).

Both **4 a** and **4 b** are formed during the protonolysis reactions, but also in minor amounts during the disfavored reverse protonolysis reaction (HN(SiHMe_2_)_2_: p*K*_a,THF_=22.6;^24^ pyrazole: p*K*_a,DMSO_=19.8).^25^ Thus, when the ratio of Me_2_pzH to [Ce{N(SiHMe_2_)}_4_] was changed to 5:1, quantitative formation of [Ce(Me_2_pz)_4_(Me_2_pzH)] (**2 b**) and four equiv of HN(SiHMe_2_)_2_ was indicated by ^1^H NMR spectroscopic analysis in [D_6_]benzene (see Figure S5). In contrast, when [Ce{N(SiHMe_2_)_2_}_4_] was treated with two or three equivalents of Me_2_pzH, decomposition occurred within minutes (see Figure S6).

The redox‐sensitive behavior of the silylamido ligand [N(SiHMe_2_)_2_] has been observed previously in rare‐earth‐metal chemistry, namely in trivalent→divalent transitions. There, treatment of [Eu^III^{N(SiMe_3_)_2_}_3_] with an excess of HN(SiHMe_2_)_2_, led to a mixed‐valent Eu^II^/Eu^III^ species,^26^ however, the oxidized organic co‐products remained unidentified. Moreover, Si−H bond activation of [N(SiHMe_2_)_2_] devoid of any metal‐based redox reaction has been found when [Ln{N(SiHMe_2_)_2_}_3_(thf)_2_] (Ln=Ce, Pr) were treated with H_2_Ndmp (e.g. dmp=2,6‐Mes_2_C_6_H_3_, see Figure 1, **D**). As a result, homoleptic complexes bearing a trianionic [N{SiMe_2_(Ndmp)}_2_] ligand were obtained along with concomitant formation of dihydrogen gas.^9^ In the latter reaction, the Si−H bond activation is driven by a powerful nucleophilic ligand, and only occurred upon displacement of coordinating thf from the initial adduct [Ln{N(SiHMe_2_)_2_}_3_(thf)_2_]. In a recent study, a cerium(III) complex bearing a carbon‐based analogue to [N(SiHMe_2_)_2_], namely [Ce{C(SiHMe_2_)_3_}_3_], was shown to exhibit pronounced Ce⋅⋅⋅H−Si β‐agostic interactions at low temperatures in solution.^27^ Upon treatment of [Ce{C(SiHMe_2_)_3_}_3_] with B(C_6_F_5_)_3_, β‐hydride abstraction occurred to afford borohydrido ligand [HB(C_6_F_5_)_3_] and disilacyclobutane as the co‐product, complementing previous similar findings.19e, 28

**Scheme 2:**
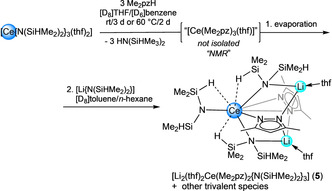
Successive treatment of [Ce{N(SiHMe_2_)_2_}_3_(thf)_2_] with Me_2_pzH and [Li{N(SiHMe_2_)_2_}] yielding [Li_2_(thf)_2_Ce(Me_2_pz)_2_{N(SiHMe_2_)_2_}_3_] (**5**) along with other cerous species.

Cerium(IV) is a powerful Lewis acid, and although structural elucidation of [Ce{N(SiHMe_2_)_2_}_4_] did indicate a degree of Ce⋅⋅⋅H−Si β‐agostic interactions,^17^ the homoleptic complex is stable. Considering that Me_2_pz is an effective nucleophile at cerium(IV) to form pyrazol‐functionalized multidentate ligands,^16^ we initially assumed Me_2_pz‐based nucleophilic chemistry to simply occur with HN(SiHMe_2_)_2_, while the ceric center acts only as a spectator. However, Si−H bond activation was not observed when the cerous [Ce{N(SiHMe_2_)_2_}_3_(thf)_2_] was reacted with Me_2_pzH (Scheme 2, Figure S7), even after heating to 60 °C for several days. It is even more remarkable that upon treatment of this reaction mixture with [Li{N(SiHMe_2_)_2_}] in [D_8_]toluene at ambient temperatures for several weeks, crystals of heteroleptic complex [Li_2_(thf)_2_Ce(Me_2_pz)_2_{N(SiHMe_2_)_2_}_3_] (**5**) were obtained. Crucially, complex **5** is stable (under inert atmospheres) despite clear Ce⋅⋅⋅H−Si β‐agostic interactions and the presence of Me_2_pz (solution ^1^H NMR spectra: Figures S8/9). Thus, the capability of the Me_2_pz ligand to initiate Si−H bond activation appears exclusively to those bound to the cerium(IV) ion.

**Scheme 3:**
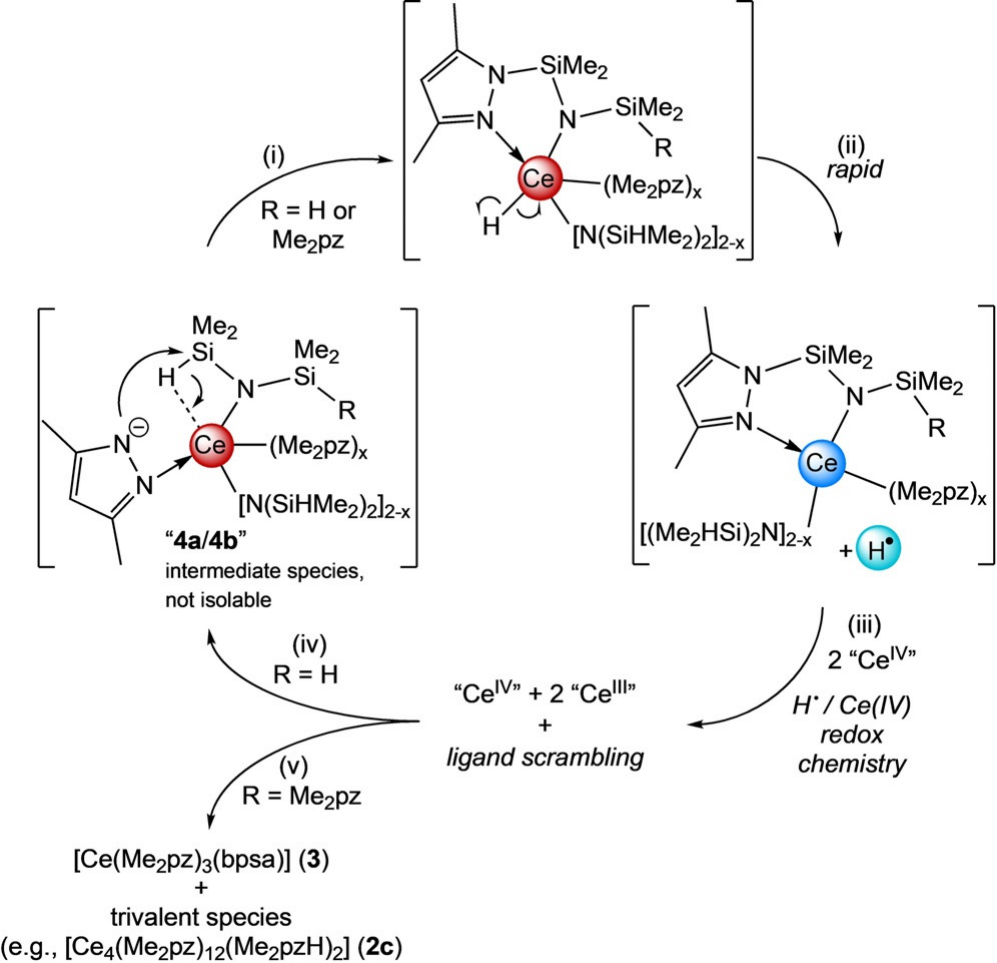
Likely formation of pincer complex **3** in the mixture [Ce{N(SiHMe_2_)_2_}_4_]/HN(SiHMe_2_)_2_ with [Ce(Me_2_pz)_4_]_2_ (**1**) as the main product. Non‐isolable heteroleptic complexes [Ce(Me_2_pz)(Me_2_pz)_*x*_{N(SiHMe_2_)_2_}_3−*x*_] (*x=*1 (**4 a**) or *x=*2 (**4 b**)) engage in cerium(IV) mediated Si−H‐bond activation and the generation of transient hydrogen radicals H^**⋅**^. The latter reduces **1** to “[Ce(Me_2_pz)_3_(Me_2_pzH)]”‐type complexes (e.g., [Ce_4_(Me_2_pz)_12_(Me_2_pzH)_2_], **2 c**). Note that this is not a catalytic cycle.

In a previous study we found that the reaction between **1** and benzophenone affords Me_2_pz‐functionalized diphenylmethoxy ligand moieties via a cascade of benzophenone association/dissociation and nucleophilic attack by the Me_2_pz co‐ligands, which is facilitated by weakening of the ketone C=O double bond upon coordination to cerium(IV).16b The present ligand transformation may bear some similarity, being triggered by the nucleophilic attack of the “activated” Si−H functionality by a pyrazolato ligand. The formation of the bpsa ligand is proposed to proceed via β‐hydride abstraction, most likely traversing a cerium(IV) hydride species (Scheme 3 (i)). The putative cerium(IV) hydride presumably undergoes immediate reduction, forming a cerium(III) species, and a hydrogen radical (Scheme 3 (ii)), which reduces other present cerium(IV) species (Scheme 3 (iii)), likely in a non‐selective manner.^29^ Along with tandem ligand scrambling, the Si−H bond activation occurs twice (Scheme 3 (iv)), ultimately giving complex **3** and other cerium(III) species (Scheme 3 (v)). Such an overall highly complicated mechanistic scenario is supported by the following observations. Although the generation of dihydrogen is observed (Figure S2), it is only detected in minor amounts. As dihydrogen does neither reduce complex **1** nor **3** at ambient temperatures, it is likely that transient hydrogen radicals reduce cerium(IV) species. Further to this, complex **3** emerged as the only remaining tetravalent species, suggesting that a degree of ligand scrambling occurred within the system, with a preference for bpsa coordination to the smaller cerium(IV) ion. As formation of the “active” heteroleptic species [Ce(Me_2_pz)_*x*_{N(SiHMe_2_)_2_}_4−*x*_] (*x=*2 (**4 a**) or *x=*3 (**4 b**)) can only occur through a disfavored reverse protonolysis reaction, it would form slowly and in minute amounts, leaving cerium(IV) in large excess and available for reaction with the highly reactive, transient hydrogen radicals. Thus, the reaction sequence is proposed to occur in the following manner: weakening of the Si−H moiety via β‐agostic interactions with the Ce^IV^ metal, pyrazolato attack at the silicon atom with concomitant Si−H‐bond cleavage and β‐hydride abstraction, cerium(IV) hydride reduction and hydrogen radical formation, finally hydrogen radical‐induced reduction of another cerium(IV) center. This reaction sequence continues until **1** is exhausted.

Considering that Ce^III^/Ce^IV^ products form relatively slowly, these complicated side reactions during the syntheses of **1** and **2 a** could be minimized by limiting the formation of **4 a** and **4 b**. Under optimized conditions crystals of [Ce{N(SiHMe_2_)_2_}_4_] are added to a stirring, concentrated solution of Me_2_pzH (in slight excess), and after addition, the solution is immediately dried under vacuum, to afford red crystals of **1** (toluene) or **2 a** (THF) in high yields.

### Reactions between [Ce(Me_2_ pz)_4_]_2_ (1) and lithium silylamides

**Scheme 4:**
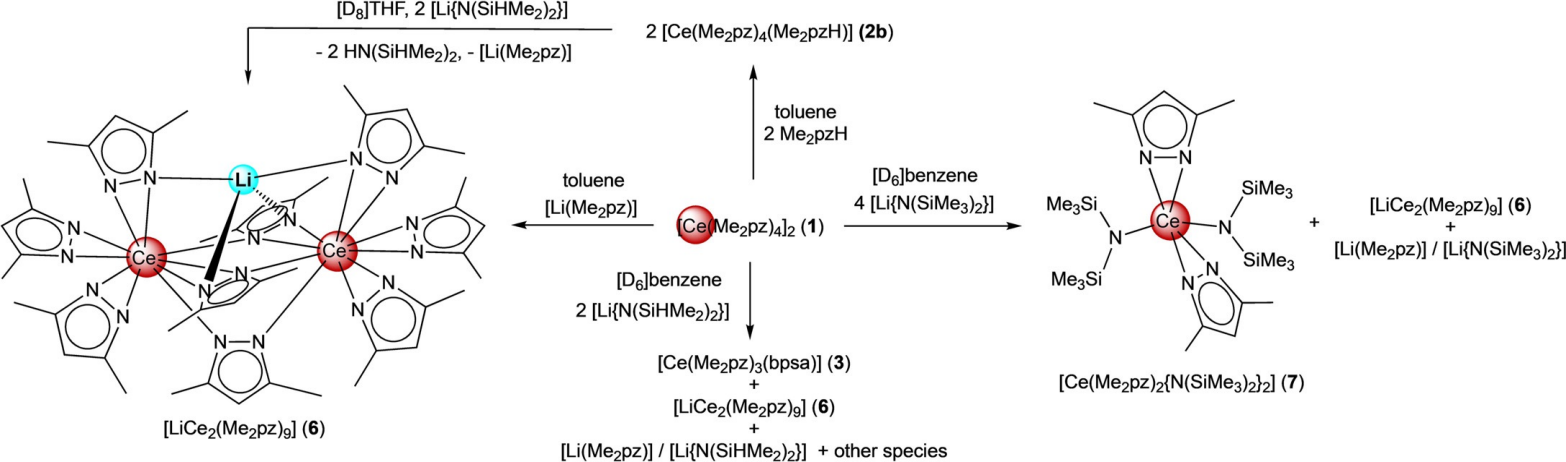
Reactions of [Ce(Me_2_pz)_4_]_2_ (**1**) with lithium reagents to afford [LiCe_2_(Me_2_pz)_9_] (**6**) and [Ce(Me_2_pz)_2_{N(SiMe_3_)_2_}_2_] (**7**).

During the syntheses of either **1** and **2 a**, we observed that lithium impurities such as Li(Me_2_pz) or lithium amide from the initial synthesis of [Ce{N(SiHMe_2_)_2_}_4_] can lead to the ate complex [LiCe_2_(Me_2_pz)_9_] (**6**). Complex **6** can be directly accessed by treating [Ce(Me_2_pz)_4_(Me_2_pzH)] (**2 b**) with an equimolar amount of [Li{N(SiHMe_2_)_2_}] in [D_8_]THF. Under inert conditions ceric **6** is thermally stable upon storage of the reaction mixture for several days at ambient temperatures, and any Si−H bond activation of the formed HN(SiHMe_2_)_2_ was not observable. Alternatively, **6** could be obtained when **1** was treated with half an equivalent of [Li(Me_2_pz)] (Scheme 4). As **6** was less soluble than both **1** and **2 a** in non‐coordinating solvents, it could be isolated by fractional crystallization and characterized by single‐crystal X‐ray crystallography. Complex **6** revealed the same structural arrangement in solution at ambient temperature as determined by ^1^H and ^13^C{^1^H} NMR spectroscopy (see Figures S10/11).

As formation of **6** appeared favorable, we envisioned that treating **1** with [Li{N(SiHMe_2_)_2_}] could provide an alternative path to form the transient heteroleptic complexes “[Ce(Me_2_pz)_*x*_{N(SiHMe_2_)_2_}_4−*x*_]” (*x=*2 (**4 a**), *x=*3 (**4 b**)) through ligand redistribution. When **1** was treated with [Li{N(SiHMe_2_)_2_}] in [D_6_]benzene, monitoring the reaction mixture by ^1^H NMR spectroscopy indicated the formation of **6**, and two different N(SiHMe_2_)_2_ environments (Si*H* septets at 6.08 and 6.14 ppm and Si*Me_2_* doublets at 0.28 and 0.34 ppm, Figures S12.1–3). These resonances are not attributable to [Li{N(SiHMe_2_)_2_}], which occur at 4.66 and 0.22 ppm, and are likely candidates for a heteroleptic Ce^IV^ species ligated by N(SiHMe_2_)_2_ (i.e. **4 a** or **4 b**), respectively. This is supported by the Si*H* shift observed in heteroleptic [Ce(L)(N{SiHMe_2_}_2_)] (L=tailored tris(hydroxylaminato), δ_H_ (Si*HMe*_2_): 6.71 and 0.89 ppm)15c and in homoleptic [Ce(N{SiHMe_2_}_2_)_4_] (δ_H_ (Si*HMe*_2_): 6.01 and 0.34 ppm).^17^ Furthermore, the resonances for HN(SiHMe_2_)_2_ were observed (4.72 and 0.11 ppm) along with the SiMe_2_ resonance attributable to the bpsa ligand (singlet at 0.13 ppm). After two days at ambient temperatures, the reaction mixture had lightened in color, and the ^1^H NMR spectrum indicated the formation of multiple Ce^III^/Ce^IV^ species (Figure S13).

To determine if such a decomposition occurs in the absence of the Si−H functionality, complex **1** was treated with lithium bis(trimethylsilyl)amide [Li{N(SiMe_3_)_2_}], in [D_6_]benzene. While ^1^H NMR spectroscopic analysis indicated a ligand redistribution process, any decomposition to paramagnetic species or formation of HN(SiMe_3_)_2_ was not observed. Instead, the following complexes were identified: the Li/Ce bimetallic complex **6**, unreacted [Li{N(SiMe_3_)_2_}], and [Li(Me_2_pz)], as well as the heteroleptic Ce^IV^ species [Ce(Me_2_pz)_2_{N(SiMe_3_)_2_}_2_] (**7**), containing two N(SiMe_3_)_2_ and two Me_2_pz ligands (Scheme 4). In stark contrast to the putative [N(SiHMe_2_)_2_] analogue **4 a**, compound **7** is thermally stable in [D_6_]benzene over several days in the presence of the corresponding silylamine (Figures S14‐16). The formation of **7** points to the likely intermediate formation of heteroleptic **4 a** when homoleptic **1** is treated with [Li{N(SiHMe_2_)_2_}]. Furthermore, the stability of **7**, which features a more sterically demanding silylamido ligand, further emphasizes that it is solely the Si−H functionality which initiates the observed redox chemistry.18b

### Monomeric ceric amide complex [Ce(Me_2_ pz)_2_{N(SiMe_3_)_2_}_2_] (7)

**Scheme 5:**
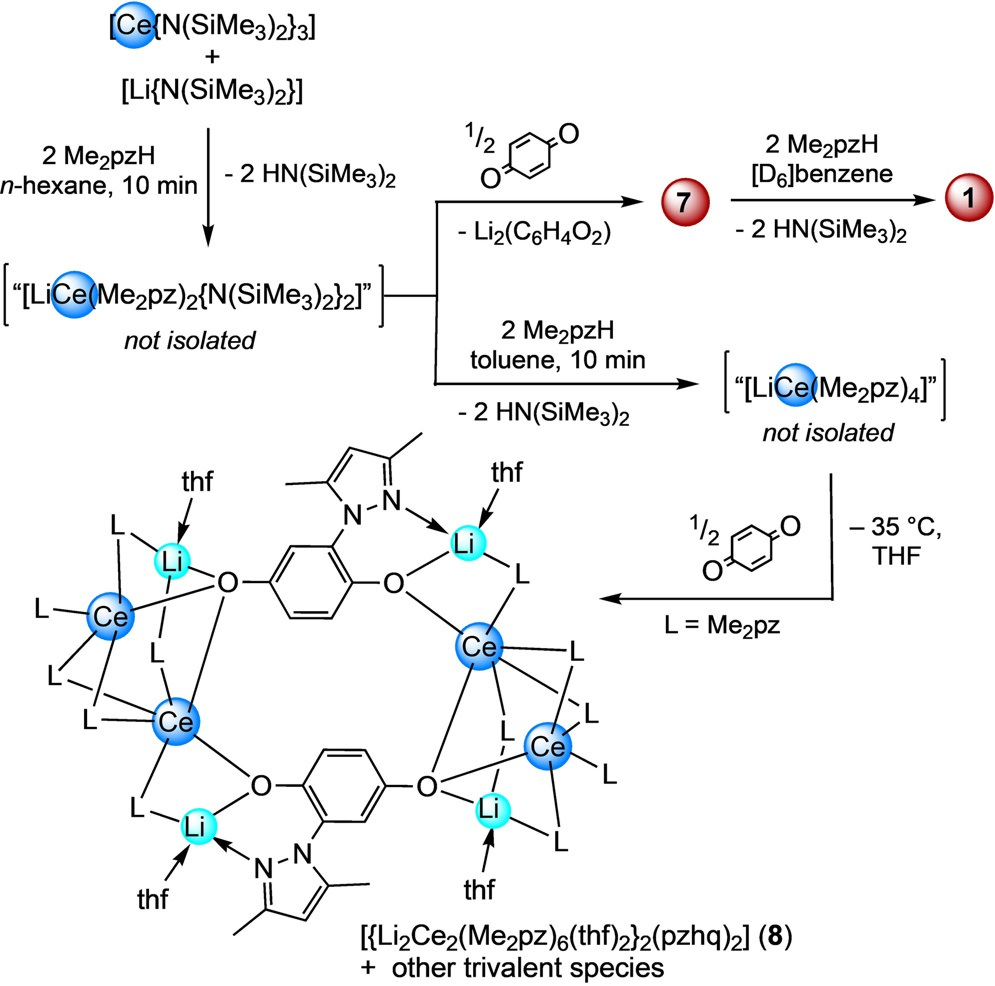
Distinct reactivity of in situ generated ate complexes “Li[Ce^III^(Me_2_pz)_2_{N(SiMe_3_)_2_}_2_]” and “Li[Ce^III^(Me_2_pz)_4_]” toward 1,4‐benzoquinone.

Complex **7** is a rare example of a ceric bis(amido) complex^30^ and can be accessed directly when 1,4‐benzoquinone is added to an in situ generated solution of “Li[Ce^III^(Me_2_pz)_2_{N(SiMe_3_)_2_}_2_]”, followed by immediate filtration (Scheme 5). It is surprising that 1,4‐benzoquinone is an effective oxidant for the isolation of complex **7**, considering our recent observations with the benzoquinone/hydroquinolate redox couple with tetravalent cerium pyrazolates.16a Further, when in situ generated “[LiCe^III^(Me_2_pz)_4_]” was treated with 1,4‐benzoquinone in THF/toluene solutions, as a means to access **1** through redox chemistry, only intractable product mixtures were observed. Fortuitous crystal formation from this reaction mixture revealed the formation of the octametallic complex [{Li_2_Ce_2_(Me_2_pz)_6_(thf)_2_}_2_(pzhq)_2_] (**8**) containing two 2‐(3,5‐dimethylpyrazol‐1‐yl)1,4‐hydroquinolato (pzhq) ligands (Scheme 5, Figure S18). Thus, in the absence of the sterically demanding N(SiMe_3_)_2_ ligands, the bq/hq and Ce^III^/Ce^IV^ redox couples and the double pyrazolato‐driven carbonyl attack dominate the reaction sequence.

Heteroleptic [Ce(Me_2_pz)_2_{N(SiMe_3_)_2_}_2_] (**7**) also proved to be an excellent precursor to quantitatively access **1** via treatment with two equivalents of Me_2_pzH (Scheme 5, Figure S17), even with an excess of **7**. More importantly, transsilylamination^31^ of **7** with HN(SiHMe_2_)_2_ was probed as a means to access putative heteroleptic species “[Ce(Me_2_pz)_2_{N(SiHMe_2_)_2_}_2_]” (**4 a**). Unfortunately, the envisaged protonolysis did not occur at ambient temperature, but was indicated at temperatures >60 °C along with decomposition (Figure S19).

### Spectroscopic and crystallographic characterization

Complexes **2 a**, **2 b**, **3**, and **7** display monomeric structures in the solid state. Compound [Ce(Me_2_pz)_4_(thf)] (**2 a**) was previously reported to crystallize from *n*‐hexane giving red block crystals with half a molecule within the asymmetric unit (monoclinic space group *P*2_1_/*m*; isostructural with the uranium analogue [U(Me_2_pz)_4_(thf)]^32^).^20^ From THF, even larger block‐like crystals were obtained (2 a*), crystallizing with three molecules in the asymmetric unit (triclinic space group P‐1). The av. Ce−N bond lengths of 2.38 Å are comparable to those of the terminal Me_2_pz ligands observed in dimeric **1** (av. Ce−N: 2.36 Å). The ^1^H NMR spectrum of **2 a** at ambient temperature shows one set of signals for the pyrazolato ligand, and magnetic susceptibility measurements (determined by the Evans method)^33^ were concordant with other cerium(IV) complexes.^34^

**Figure 2:**
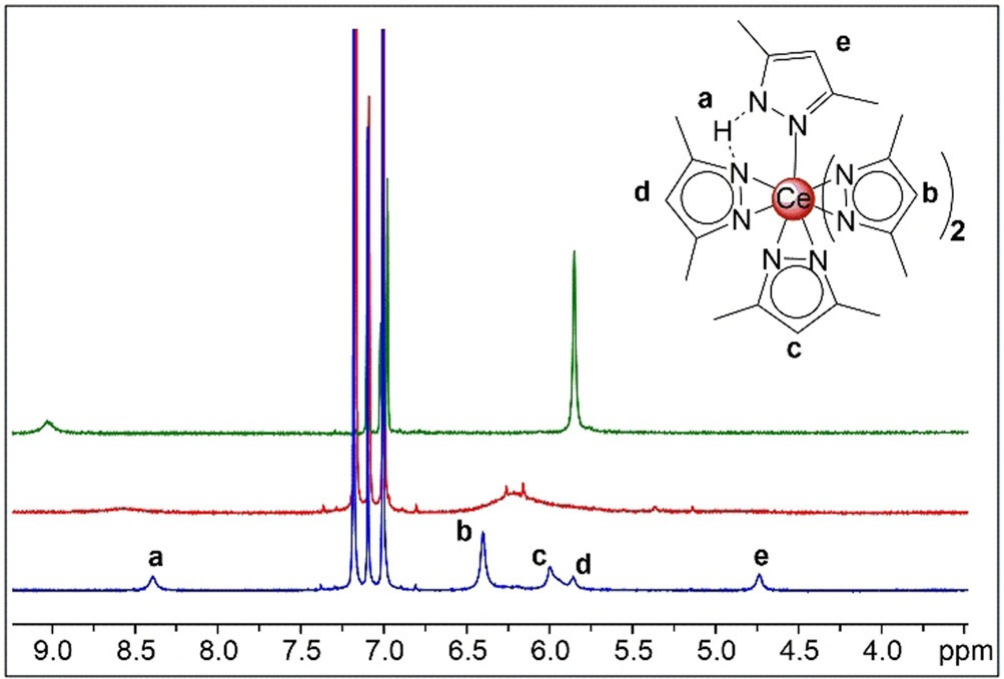
^1^H NMR spectra ([D_8_]toluene, 500 MHz) of [Ce(Me_2_pz)_4_(Me_2_pzH)] (**2 b**) at 26 °C (top, green), −80 °C (middle, red), and −100 °C (bottom, blue).

The Me_2_pzH‐ligated monomeric complex **2 b** adopts the same structural motif as **2 a**, with three Me_2_pz ligands on the meridional plane and the remaining pyrazole/pyrazolato ligands disordered 50:50 over both axial positions. Unfortunately, the structural solution was not straightforward. The compound crystallized with significant molecular disorder, with the entire molecule split over two positions (see Figure S20). When **2 b** was analyzed by ^1^H NMR spectroscopy, only one ligand environment was observed as revealed by sharp C*H*_3_ and C*H* resonances, and a broadened N*H* signal, shifted to higher field (9.03 ppm) compared to free Me_2_pzH (12.25 ppm, Figures S21/22). This indicates a high proton mobility among the pyrazolato/pyrazole ligands in solution (Figure 2, green spectrum).

To identify any distinct pyrazolato/pyrazole environments in **2 b**, variable temperature ^1^H NMR spectroscopic experiments were performed (Figures 2 and S23). When analyzed at −80 °C, the resonances had broadened significantly (Figure 2, red). Upon further cooling to −100 °C, the resonances decoalesced to identify four different ligand C(4)‐*H* environments (Figure 2, blue), integrating (from left to right) in a ratio of 2:1:1:1. In order to assist with peak assignment, the thf analogue, **2 a**, was also analyzed by ^1^H NMR spectroscopy at −100 °C, showing two pyrazolato C(4)‐*H* resonances in a ratio of 3:1, corresponding to magnetically distinct equatorial (6.34 ppm) and axial (5.91 ppm) pyrazolato ligands (Figures S23–26). Using these assignments as a basis for peak assignment in **2 b**, the far downfield shifted C(4)‐*H* resonance (*e*, Figure 2) corresponds to the Me_2_pzH ligand, which also has two different methyl environments for the 3‐ and 5‐positions (at 2.10 and 0.99 ppm). In accord with the ^1^H NMR spectrum of **2 a**, the axial Me_2_pz ligand (*c*, Figure 2), and two of the equatorial ligands (*b*, Figure 2) resonate in a similar region. However, the third C*H* resonance (*d*, Figure 2) indicates that one equatorial pyrazolato ligand is interacting with the nitrogen bound H atom of the pyrazole ligand (*a*, Figure 2). Such interactions between pyrazole‐H and pyrazolato ligands are well established in rare‐earth‐metal solid‐state chemistry,^35^ but weren't observed on the time scale of NMR spectroscopy. Furthermore, it should be noted that in hafnium complex [Hf(Me_2_pz)_4_(Me_2_pzH)] such pz⋅⋅⋅H‐pz interactions were observed crystallographically and that the ^1^H NMR spectra at ambient temperature are almost identical to **2 b**, with a broadened N*H* resonance at 13.0 ppm, and one resonance assignable to both Me_2_pz and Me_2_pzH (at 2.12 and 5.89 ppm).^36^ Unfortunately, additional low‐temperature NMR investigations such as ^13^C NMR spectroscopy were unsuccessful. The tetravalent oxidation state of **2 b** was difficult to confirm by X‐ray crystallography, due to the disorder, but surprisingly also by magnetic susceptibility measurements. Upon analysis of **2 b**, a large Δ*H* was observed between the two reference peaks giving a *μ*_eff_ of 2.43 BM (a value typically higher than those of cerium(III) complexes).^37^ Such a value is possibly caused by the hydrogen exchange between the pyrazole and the pyrazolato ligands. Nevertheless, the color of the complex, and the ability to form **2 b** by treating **1** with one equivalent of Me_2_pzH, suggests that **2 b** is indeed tetravalent.

**Figure 3:**
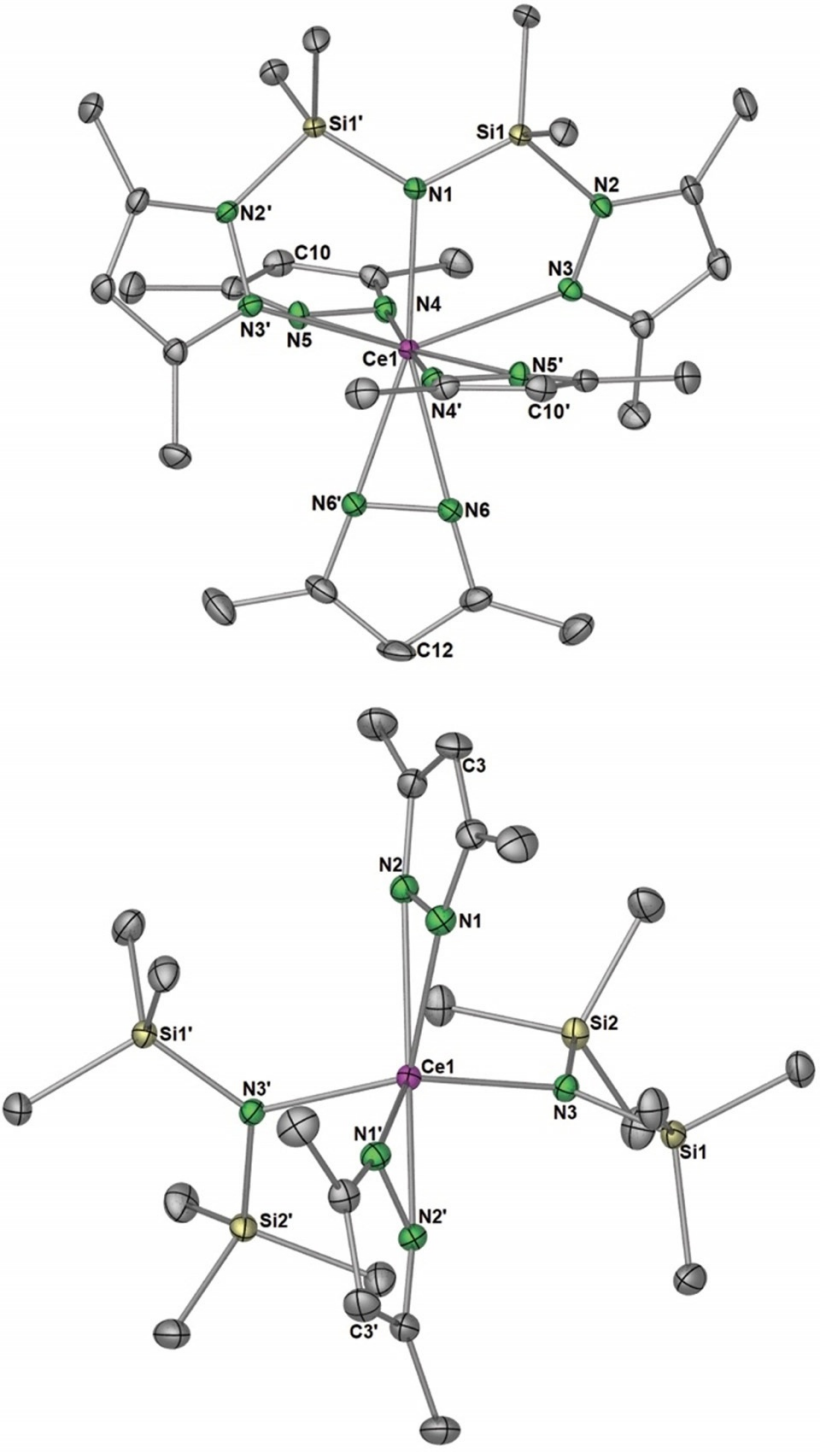
Crystal structures of monomeric cerium(IV) species [Ce(Me_2_pz)_3_(bpsa)] (**3**, top) and [Ce(Me_2_pz)_2_{N(SiMe_3_)_2_}_2_] (**7**, bottom). Ellipsoids shown at 50 % probability, hydrogen atoms omitted for clarity. Selected bond lengths (Å) and angles (°) for **3**: Ce‐N(1) 2.350(3), Ce‐N3 2.598(2), Ce‐N4 2.395(2), Ce‐N5 2.372(2), Ce‐N6 2.411(2); N3‐Ce‐N3′ 146.23(9), N1‐Ce1‐N3 73.11(4), N1‐Ce1‐C12 180.0(2). For **7**: Ce‐N(1) 2.330(3), Ce‐N2 2.396(2), Ce‐N3 2.217(2); C3‐Ce1‐C3′ 118.71(6), N3‐Ce1‐N3 128.20(9), N3‐Ce1‐N1/2(centroid) 110.70(4).

The X‐ray data for heteroleptic complex **3** were solved and refined in the monoclinic space group *C*2/*c*, where half of the molecule resided within the asymmetric unit (Figure 3, *top*). The bpsa ligand has a large coordination bite angle across the 6‐coordinate cerium center (N3‐Ce1‐N3′, 146.23(9)°). Considering the unique nature of the bpsa ligand, it was difficult to find a suitable literature comparison, however, the coordination of bis(2‐methoxyethyl)ether in tetravalent [Ce{OCH(CF_3_)_2_}_4_(diglyme)] (diglyme=bis(2‐methoxyethyl)ether) appeared a suitable candidate. There, the diglyme ligand binds across the equatorial position with two OCH(CF_3_)_2_ ligands, giving a diglyme coordination angle of 124.26(16)°. This angle is considerably smaller compared to the one of bpsa in **3** and is likely due to the more compact nature of diglyme and the 7‐coordinate cerium center.^38^ The Ce‐N(amido) bond length of the [NNN] pincer of 2.350(3) Å is shorter than the Ce−N(pz) bond lengths (av. 2.393 Å) and the pincer‐pyrazolato distances of av. 2.598 Å. The structural arrangement of **3** is retained in solution, with signal sets in the ^1^H NMR spectrum showing two C(4)‐*H* environments in a 2:3 ratio, and three methyl group resonances in a 3:1:1 ratio, with the methyl group at the 3‐position of the Me_2_pz substituents on the bpsa ligand resonating significantly shifted downfield than those in 5‐position (Figure S27). The tetravalent oxidation state was further confirmed by the Evans method, which gave a *μ_eff_* of approximately zero.

The X‐ray data for heteroleptic **7** were solved in the monoclinic space group *C*2/*c* with half the molecule within the asymmetric unit (Figure 3, *bottom*). Ceric complex **7** adopts a tetrahedral geometry reminiscent of that in Ce^IV^ complex [Ce(O*t*Bu)_2_{N(SiMe_3_)_2_}_2_] (Ce−N 2.260(7) Å),^39^ and the amido bond lengths of 2.2172(17) Å compare well with the latter. The ^1^H NMR spectrum of **7** supports the same structure in solution, with signal sets for Me_2_pz and N(SiMe_3_)_2_ in a 1:1 ratio (Figures S15/16). The tetravalent oxidation state was further corroborated by the magnetic susceptibility measurements, determined by the Evans method.

**Figure 4:**
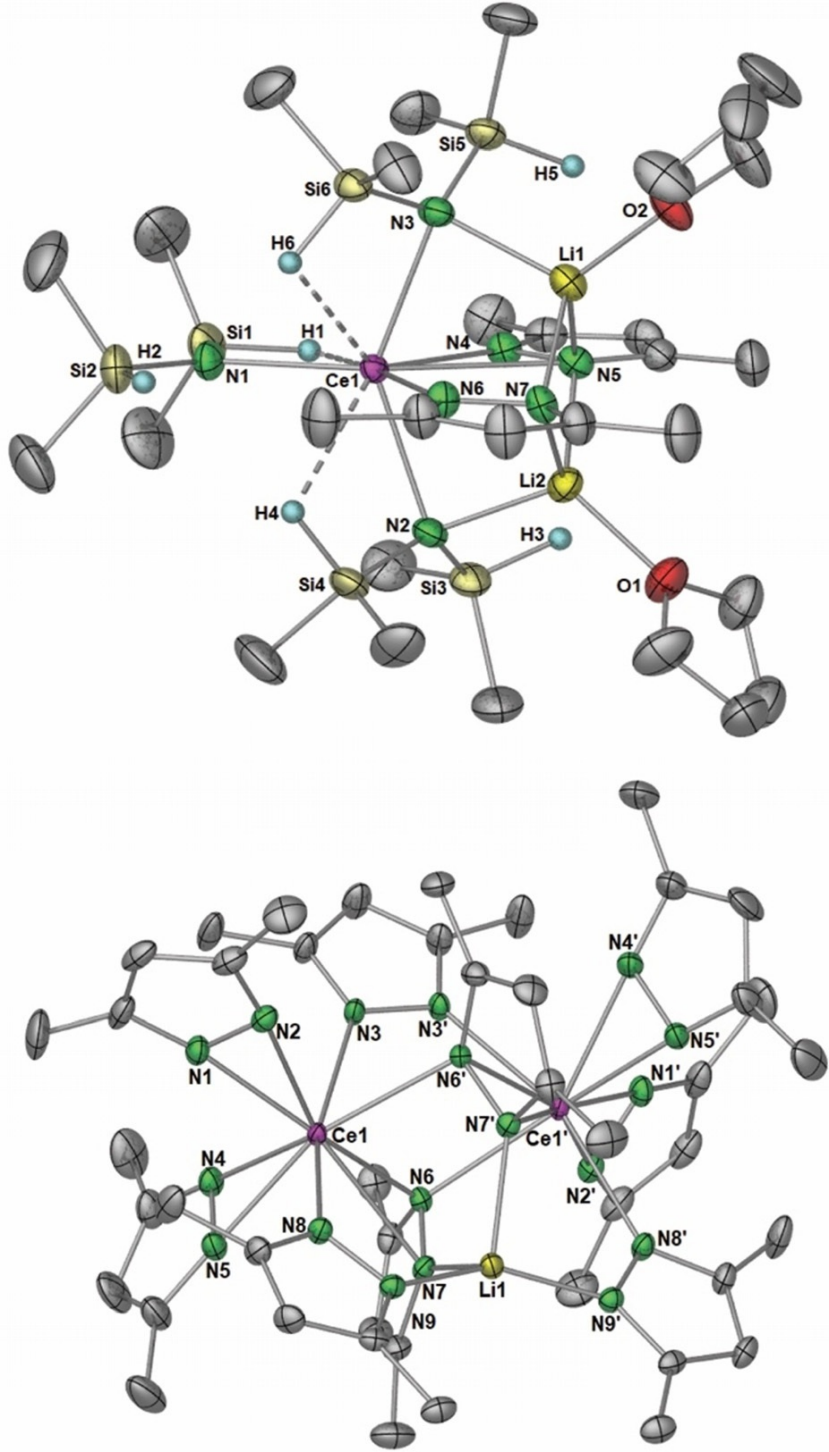
Crystal structures of [Li_2_(thf)_2_Ce(Me_2_pz)_2_{N(SiHMe_2_)_2_}_3_] (**5**, *top*), and [LiCe_2_(Me_2_pz)_9_] (**6**, *bottom*, cf; Scheme 4). Ellipsoids are shown at 50 % probability. Hydrogen atoms and lattice solvent removed for clarity. Selected bond lengths (Å) for **5**: Ce1‐N1 2.392(2), Ce1‐N2 2.516(2), Ce1‐N3 2.524(3), Ce1‐N4 2.508(2), Ce1‐N5 2.926(2), Ce1‐N6 2.573(3), Ce1⋅⋅⋅H1 2.79(3), Ce1⋅⋅⋅H4 2.74(3), Ce1⋅⋅⋅H6 2.67(2), Li1‐N3 2.163(4), Li1‐N5 2.155(4), Li1‐N7 2.142(4), Li2‐N2 2.159(4), Li2‐N5 2.176(4), Li2‐N7 2.104(7), Li1‐O2 1.987(4), Li2‐O1 1.964(4). **6**: Ce‐N1 2.297(2), Ce‐N2 2.388(2), Ce‐N3 2.533(3), Ce‐N4 2.385(3), Ce‐N5 2.336(3), Ce‐N6 2.523(3), Ce1′‐N6 2.503(2), Ce1′‐N7 2.633(2), Ce1‐N8 2.489(2), Li1‐N7 2.062(4), Li1‐N9 1.971(3).

The X‐ray diffraction data of lithium‐incorporated cerous ate complex [Li_2_(thf)_2_Ce(Me_2_pz)_2_{N(SiHMe_2_)_2_}_3_] (**5**, monoclinic space group *P*2_1_/*c*) allowed for the manual assignment of the hydrogen atoms of the Si−H groups and hence, the elucidation of Ce⋅⋅⋅H−Si β‐agostic interactions of the tilted amido ligand (Figure 4, *top*). Accordingly, the shortest Ce⋅⋅⋅H and Ce⋅⋅⋅Si distances of 2.67(2)‐2.79(3) Å and 3.2370(7)‐3.2648(7) Å, respectively, are similar to those observed in the heteroleptic cerium(III) dimethylsilylamide bis(pentamethylcyclopentadienyl) complex [Cp*_2_Ce{N(SiHMe_2_)_2_}] (2.65(2)‐2.77(2) Å).^40^ Each lithium atom is encapsulated by a terminal thf ligand, and a bridging silylamido, and both are coordinated by two pyrazolato ligands in κ^1^(*N′*) fashion.

The heterobimetallic ceric complex [LiCe_2_(Me_2_pz)_9_] (**6**) crystallized in the monoclinic space group *C*_2_/*c*. The crystal structure features four different Me_2_pz environments. Four pyrazolato ligands coordinate in a typical η^2^(*N*,*N*′) terminal fashion to cerium, and cap the edges of the complex. Three Me_2_pz ligands coordinate in an *μ*‐1κ(N)‐2κ(N′) fashion, bridging between either cerium atoms, or lithium and cerium atoms, while the two remaining ones bridge between all three metal atoms in a *μ*_3_‐η^2^(*N*,*N*′):2κ(N):3κ(N′) fashion (Figure 4, *bottom*). The ^1^H NMR spectrum at ambient temperature revealed distinct signal sets for each pyrazolato environment (Figures S10/11). The four terminal Me_2_pz ligands resonate at 6.00 ppm, while the signals of the bridging ones are located at 5.76, 5.73 and 4.75 ppm with an integral ratio of 1:2:2. Such a difference in chemical shift was also observed when dimeric **1** was analyzed at −80 °C, which led to decoalescence of the bridging and terminal Me_2_pz ligand resonances (5.04 and 6.00 ppm).16b Magnetic susceptibility measurements determined by the Evans method further confirmed that **6** is diamagnetic, giving a *μ_eff_* value of approximately zero.

### Comparisons of selected spectroscopic features

Selected spectroscopic characterization data for complexes **1**–**3**, **6**, and **7** are presented in Table 1 (data for [Ce(Me_2_pz)_4_]_2_ (**1**) and **2 a** obtained as previously reported).16b, 20 The ^1^H NMR spectra for complexes **1**, **2 a**, and **2 b** all show only one Me_2_pz environment at ambient temperatures. At lower temperatures, decoalescence of the resonances can be observed in line with the respective solid‐state structures. In general, the resonances for the terminal/equatorial Me_2_pz ligands occur at lower fields than those of the bridging/axial ligands. This is also reflected in the LiCe_2_ ate complex **6**, where the Me_2_pz ligand bridges between all three metal centers to resonate at a comparatively higher field. The IR spectra show the Me_2_pz *v̄*(CN) absorption at 1521‐1516 cm^−1^ for complexes **1**–**3**, **6**, and **7**, with an additional band at 1538 cm^−1^ in **2 b**, corresponding to the Me_2_pzH ligand. Such spectroscopic features are consistent with other rare‐earth‐metal pyrazolate complexes.^22^ The UV/Vis spectra of all compounds as depicted in Figures S28–32 revealed molar absorptivities consistent with ligand to metal charge transfers in other Ce^IV^ systems,18a, 21, 41 e.g., homoleptic [Ce{N=C(NMe_2_)_2_}_4_]_2_ (17 155 and 12 078 mol^−1^ cm^−1^),^21^ or [Ce{N(SiMe_3_)_2_}_3_Cl] (*ϵ*=2520 mol^−1^ cm^−1^),^42^ Notably, the mono‐solvated [Ce(Me_2_pz)_4_(thf)] (**2 a**) complex exhibits a significantly larger molar absorptivity of 15 000 mol^−1^ cm^−1^ compared with [Ce(Me_2_pz)_4_]_2_ (**1**, 7900 mol^−1^ cm^−1^). For further comparison, the Me_2_pzH analogue **2 b** gave a considerably lower value of 5100 mol^−1^ cm^−1^. Such a difference highlights the significant effects of the coordinating co‐ligands on such L→M charge transfer complexes in rare‐earth‐metal chemistry, with the bpsa complex showing the highest absorptivity of 20 000 mol^−1^ cm^−1^.

**Table 1:** Spectroscopic data for cerium(IV) complexes under study

Compound	Yield (%)	^1^H NMR* (ppm) r.t.	^1^H NMR* (ppm) vt	UV/Vis (*λ*_max_)	*ϵ* (mol^−1^ cm^−1^)	*μ*_eff_ (BM)
[Ce(Me_2_pz)_4_]_2_ (**1**)16a	70,16a (95)	5.8416a	5.04/6.00^[16a][a]^	420	7900	1.22
[Ce(Me_2_pz)_4_(thf)] (**2 a**)^20^	96	6.13	5.91/6.34^[b]^	418	15 000	1.05
[Ce(Me_2_pz)_4_(Me_2_pzH)] (**2 b**)	>99	5.93	4.65/5.77/5.91/6.32^[b]^	409	5100	2.43
[Ce(Me_2_pz)_3_(bpsa)] (**3**)	≈8	5.95/6.00	–	422	20 000	≈0
[LiCe_2_(Me_2_pz)_9_] (**6**)	41	4.75/5.73/5.76/6.00	–	423	10 400	≈0
[Ce(Me_2_pz)_2_{N(SiMe_3_)_2_}_2_] (**7**)	27 (crystal yield)	6.21	–	430	3800	1.02

[a] Performed at −80 °C. [b] performed at −100 °C. * ^1^H NMR resonance of the pyrazolato methine.

As mentioned above, the magnetic susceptibility for complexes **1**–**7** were determined by the Evans method,^25^ and except complex **2 b**, each species gave a value consistent with other Ce^IV^ complexes. It appears that ligand exchange processes (or NH hydrogen exchange in **2 b**) result in an increased Δ*H*z between the two reference peaks in the NMR spectrum, consequentially giving larger magnetic susceptibility values when analyzed by this method. In contrast, more inflexible ligand arrangements (e.g. those in **6**, or **3**) give values of approximately zero. Thus, this spectroscopic method might be not suitable to determine the tetravalent oxidation state for systems with a high degree of solution‐based ligand dynamics.

## Conclusions

Tetravalent cerium complex [Ce{N(SiHMe_2_)_2_}_4_] revealed competing reactivities toward dimethylpyrazole. Although, the protonolysis reaction gives the desired [Ce(Me_2_pz)_4_]_2_ (or [Ce(Me_2_pz)_4_(thf)]) in an efficient manner, an alternative path involves Si−H‐bond activation and hydride abstraction at cerium(IV), followed by rapid reduction to cerium(III) and a hydrogen radical. The latter complicated reaction sequence accomplishes the [NNN] dipyrazolyl pincer ligated [Ce(Me_2_pz)_3_(bpsa)] as a minor product, involving heteroleptic species of type “[Ce(Me_2_pz)_*x*_{N(SiHMe_2_)_2_}_4−*x*_]” (*x=*2, 3) as intermediates. In the absence of the Si−H functionality such pincer co‐products were not observed. In accordance, treatment of [Ce(Me_2_pz)_4_]_2_ with [Li{N(SiMe_3_)_2_}] afforded two isolable ceric complexes, bimetallic [LiCe_2_(Me_2_pz)_9_] and heteroleptic [Ce(Me_2_pz)_2_{N(SiMe_3_)_2_}_2_]. Crucially, the observed hydride abstraction at the dimethylsilylamido moiety only occurred in cerium(IV) systems and, for comparison, not in reactions with the weaker Lewis acidic cerium(III) compounds. Such findings highlight an additional synthetic scope of cerium(IV) in chemical transformations, and provide a potential pathway for obtaining cerium(IV) hydride species. Future work will involve determining a direct high‐yielding protocol for the bpsa ligand, since our initial attempt of the noncerium(IV) mediated reaction of [Li{N(SiHMe_2_)_2_}] and [Li(Me_2_pz)] did not lead to the desired ligand framework (Figure S33), further highlighting the importance of tetravalent cerium.

## Experimental Section

***General methods, instrumentation, and starting materials***: All manipulations were performed using glovebox (MBraun 200B; <0.1 ppm O_2_, <0.1 ppm H_2_O) or Schlenk techniques under an atmosphere of purified argon gas, in oven dried glassware. Solvents THF, *n*‐hexane, and toluene were purified by Grubbs‐type columns (MBraun SPS, solvent purification system), while cyclohexane, [D_8_]THF, [D_8_]toluene, and [D_6_]benzene were dried over NaK alloy and degassed. All solvents were stored inside a glovebox. Me_2_pzH was purchased from Sigma Aldrich and used as received. Anhydrous CeCl_3_ was purchased from ABRC chemicals and was activated by Soxhlet extraction with THF, giving [CeCl_3_(thf)_1.05_]. [Li{N(SiHMe_2_)_2_}] was synthesized according to published procedures.^43^ [Ce{N(SiHMe_2_)_2_}_3_(thf)_2_] was synthesized by treatment of [CeCl_3_(thf)_1.05_] with three equivalents of [Li{N(SiHMe_2_)_2_}] in *n*‐hexane and was purified by filtration, evaporation to dryness and crystallization from *n*‐hexane. 1,4‐Benzoquinone was purchased from Sigma–Aldrich and sublimed before use. [Ce{N(SiHMe_2_)_2_}_4_] and [Ce(N*i*Pr_2_)_4_] were synthesized according to the literature,17b, 18b, 21 the former involving oxidation of the ate complex [Li(thf)Ce{N(SiHMe_2_)_2_}_4_] with 1,4‐benzoquinone in toluene. The NMR spectra of air‐ and moisture‐sensitive compounds in [D_6_]benzene or [D_8_]THF were recorded with J.Young‐valved NMR tubes. Unless specified otherwise, analyses were performed at 26 °C with either a Bruker‐Avance II 400 (^1^H: 400 MHz, ^13^C: 101 MHz), a Bruker‐Avance II 500 (^1^H: 500 MHz; ^13^C: 126 MHz) or a Bruker DRX‐250 (^1^H: 250 MHz, ^13^C: 63 MHz) spectrometer. Magnetic susceptibilities were determined by the Evans method.^33^ Infrared spectra were recorded on a Nicolet 6700 FTIR spectrometer (*ṽ*=4000‐600 cm^−1^) by using a DRIFT chamber with dry KBr/sample mixtures and KBr windows. Elemental analyses (C, H, N) were performed on the bulk sample (unless specified otherwise), with an Elementar Vario Micro cube by Mr. W. Bock (EKUT). Unless specified otherwise, reported yields are given for compounds after a satisfactory microanalysis was obtained from the bulk sample. [Ce(Me_2_pz)_4_]_2_ (**1**) was initially synthesized by a literature procedure.16a Note that dimeric **1** could be isolated with varied degrees of toluene solvation, namely either [Ce(Me_2_pz)_4_]_2_⋅^1^/_2_PhMe or [Ce(Me_2_pz)_4_]_2_⋅^1^/_4_PhMe. The complex used was treated as a monomeric species (e.g. [Ce(Me_2_pz)_4_]⋅^1^/_8_PhMe) for stoichiometric calculations.

### Products obtained from protonolysis reactions

**Optimized synthesis of [Ce(Me_2_** **pz)_4_]_2_ (1)**: [Ce{N(SiHMe_2_)_2_}_4_] (120 mg, 0.18 mmol) was intermittently added (over a 30 second period) to a concentrated, stirring, toluene solution of Me_2_pzH (70.0 mg, 0.71 mmol). After addition, the sample was immediately evaporated to dryness leaving a red microcrystalline powder of **1** (92.7 mg, 96 %). The spectroscopic data were consistent with those previously published.16a

### [Ce(Me_2_ pz)_4_(thf)] (2 a)

*Method a*, *in THF*: [Ce{N(SiHMe_2_)_2_}_4_] (99.6 mg, 0.15 mmol) was dissolved in THF, and added to a THF solution of Me_2_pzH (57.0 mg, 0.62 mmol) at ambient temperature. The reagents were shaken for 30 seconds before immediate exposure to vacuum. Concentration, filtration, and storage at −35 °C, gave large red block crystals of [Ce(Me_2_pz)_4_(thf)] (2 a*, 39.0 mg, 42 %, crystals indicated three molecules of **2 a** within the asymmetric unit). ^1^H NMR ([D_6_]benzene, 250 MHz, 300 K): *δ*=6.13 ppm (s, 4 H, C*H*), 3.31 (m, 4 H, α‐thf), 2.34 (s, 24 H, C*H*_3_), 0.94 (m, 4 H, β‐thf); ^13^C{^1^H} NMR ([D_6_]benzene, 63 MHz, 300 K): *δ*=144.6 ppm (s, Me_2_pz‐C(4)), 112.6 (s, Me_2_pz‐C(3,5)), 70.3 (s, α‐thf‐*C*H_2_(1,4)), 25.2 (s, β‐thf‐*C*H_2_(2,3)), 13.5 (s, *C*H_3_); DRIFT: *v̄*=3099 (w), 3024 (w), 3013 (s), 2942 (s), 2878 (s), 2859 (m), 1517 (vs), 1474 (w), 1470 (w), 1455 (w), 1444 (m), 1432 (vs), 1417 (s), 1364 (m), 1315 (w), 1301 (w), 1106 (w), 1050 (w), 1028 (w), 1006 (m), 959 (w), 921 (w), 871 (m), 806 (w), 781 (w), 728 (w) cm^−1^; χ_mol_=4.64×10^−4^ cm^3^ mol^−1^, *μ*_eff_=1.05 BM (Δ*H*z: 1.2 Hz, 0.033 mol L^−1^); UV(toluene): *λ*_max_=418 (*ϵ*=15 000 mol^−1^ cm^−1^), 316 nm (*ϵ*=8400 mol^−1^ cm^−1^).

*Method b, in THF and cyclohexane*: see ref. [20]

*Method c, the optimized synthesis*: Crystals of [Ce{N(SiHMe_2_)_2_}_4_] (118.0 mg, 0.18 mmol) were intermittently added (over a 30 second period) to a concentrated, stirring, THF solution of Me_2_pzH (68.0 mg, 0.71 mmol) at ambient temperature. After addition, the sample was immediately evaporated to dryness leaving a red microcrystalline powder of **2 a** (100.6 mg, 96 %). The ^1^H NMR spectrum was consistent with the data of the sample obtained by method a. ^1^H NMR ([D_8_]toluene, 400 MHz, 173 K): *δ*=6.34 (br s, 3 H, Me_2_pz‐C*H*_equatorial_), 5.91 (br s, 1 H, Me_2_pz‐C*H*_axial_), 3.08 (s, 4 H, β‐thf), 2.47 (br s, 18 H, Me_2_pz‐C*H*_3equatorial_), 2.11 (br s, 6 H, Me_2_pz‐C*H*_3axial_), 0.36 (s, 4 H, β‐thf) ppm. Elemental analysis calcd (%) for C_24_H_36_CeN_8_O (592.71 g mol^−1^): C 48.63, H 6.12, N 18.91; found: C 48.47, H 5.56, N 19.16.

### [Ce(Me_2_ pz)_4_(Me_2_ pzH)] (2 b)

*Method a, with an excess of Me_2_pzH*: [Ce{N(SiHMe_2_)_2_}_4_] (74.5 mg, 0.11 mmol) was dissolved in cyclohexane and added to a toluene solution of excess Me_2_pzH (53.0 mg, 0.55 mmol) at ambient temperature. The reaction mixture was concentrated and stored at −35 °C, giving dark red crystals of [Ce(Me_2_pz)_4_(Me_2_pzH)] (**2 b**, crystal yield: 17.0 mg, 25 %). ^1^H NMR ([D_6_]benzene, 400 MHz, 300 K): *δ*=9.03 ppm (s, 1 H, N−*H*), 5.93 (s, 5 H, C−*H*), 2.20 (s, 30 H, C*H*_3_); ^13^C{^1^H} NMR ([D_6_]benzene, 100 MHz, 300 K): *δ*=144.6 ppm (s, Me_2_pz‐*C*(4)), 110.9 (s, Me_2_pz‐*C*(3,5)), 13.0 (s, Me_2_pz‐*C*H_3_); ^1^H NMR ([D_8_]toluene, 500 MHz, 173 K,): *δ*=8.30 (br s, 1 H, N*H*), 6.32 (s, 2 H, C*H*), 5.91 (br s, 1 H, C*H*), 5.77 (br s, 1 H, C*H*), 4.65 (br s, 1 H, C*H*), 2.47 (br s, 18 H, C*H*_3_), 2.16 (br s, 3 H, C*H*_3_), 2.10 (br s, 6 H, C*H*_3_), 0.89 (br s, 3 H, C*H*_3_) ppm; DRIFT: *v̄*=3348 (br m), 3105 (w), 2940 (m), 2916 (m), 2870 (m), 1566 (m), 1538 (m), 1516 (vs), 1486 (w), 1470 (s), 1456 (s), 1430 (vs), 1418 (vs), 1373 (m), 1363 (s), 1302 (m), 1275 (s), 1153 (w), 1102 (w), 1029 (w), 1016 (m), 1006 (m), 957 (w), 782 (m), 728 (m) cm^−1^. Elemental analysis calcd (%) for C_25_H_36_CeN_10_ (616.75 g mol^−1^): C 48.69, H 5.88, N 22.71; Found: C 48.44, H 5.42, N 22.72; χ_mol_=0.00247 cm^3^ mol^−1^, *μ*_eff_=2.43 BM (Δ*H*z: 36 Hz, 0.0022 mol L^−1^); UV(toluene): *λ*_max_=409 (*ϵ*=5100 mol^−1^ cm^−1^), 321 nm (*ϵ*=2800 mol^−1^ cm^−1^).

*Method b,* ^*1*^ *H NMR‐scale experiment*: [Ce{N(SiHMe_2_)_2_}_4_] (15.1 mg, 0.026 mmol) and Me_2_pzH (11.0 mg, 0.11 mmol) were combined in [D_6_]benzene at ambient temperature indicating complete conversion to [Ce(Me_2_pz)_4_(Me_2_pzH)] and HN(SiHMe_2_)_2_. ^1^H NMR ([D_6_]benzene, 400 MHz, 300 K): *δ*=0.12 (d, 48 H, Si(C*H*_3_)_2_), 2.20 (s, 30 H, C*H*_3_), 4.72 (septet, 6 H, Si*H*, lower integration than expected), 6.09 (s, 5 H, C*H*), 9.00 ppm (s, 1 H, N*H*).

### Ce(Me_2_ pz)_3_(bpsa)] (3)

*Method a, heating reaction mixture in* [D_6_]benzene: [Ce{N(SiHMe_2_)_2_}_4_] (23.8 mg, 0.036 mmol) and Me_2_pzH (13.0 mg, 0.014 mmol) were combined and shaken in [D_6_]benzene. After 30 min, the reaction mixture was heated in a J. Young‐valved NMR tube at 70 °C for 16 h giving a light red solution (refer to Figure S1). The contents were transferred into a glovebox and evaporated to dryness (in vacuo). *n*‐Hexane was added, and the sample was filtrated and stored at −35 °C, giving light red plate‐like crystals of [Ce(Me_2_pz)_3_(bpsa)] (approximate yield: 2 mg, ≈8 %), along with a colorless precipitate. The crystals were separated by hand picking. Elemental analysis calcd (%) for C_29_H_47_CeN_11_Si_2_ (746.06 g mol^−1^, performed on two handpicked crystals, dried under vacuum prior to analysis): C 46.69, H 6.35, N 20.65; Found: C 47.27, H 5.56, N 20.42; χ_mol_=−2.39×10^−4^ cm^3^ mol^−1^, *μ*_eff_=−0.07 BM (Δ*H*z: −3, 3.0×10^−3^ mol L^−1^).

*Method b, from a THF solution*: From the attempted synthesis of **2 a** (see above), the reaction mixture was allowed to stand at ambient temperature for several weeks. Upon concentration and *n*‐hexane layering, storage at −35 °C gave light red crystals of **3** amongst a colorless microcrystalline powder. ^1^H NMR ([D_6_]benzene, 400 MHz, 300 K, trace solvent peaks not included): *δ*=6.00 (br s, 3 H, Me_2_pz‐C*H*), 5.95 (br s, 2 H, Me_2_pzSiMe_2_‐C*H*), 2.82 (br s, 6 H, Me_2_pzSiMe‐C*H*_3_), 2.10 (br s, 6 H, Me_2_pzSiMe_2_‐C*H*_3_), 2.03 (s, 18 H, Me_2_pz‐C*H*_3_), 0.13 (s, 12 H, Si(C*H*_3_)_2_) ppm. UV(*n*‐hexane): *λ*_max_=422 (*ϵ*=20 000 mol^−1^ cm^−1^).

**Protonolysis reaction using [Ce(N*i*Pr_2_)_4_] and Me_2_** **pzH**: [Ce(N*i*Pr_2_)_4_] (153.5 mg, 0.28 mmol), was dissolved in toluene (1 mL), and a solution of Me_2_pzH (110.0 mg, 1.1 mmol), was added under vigorous stirring. The flask was shaken and within seconds the color changed from dark blue to light red. Crystallization from *n*‐hexane gave colorless single crystals of trivalent complex [Ce(Me_2_pz)_3_(Me_2_pzH)]_2_. The unit cell was in accord with the literature data.^18^ Crystal yield ≈50 mg (≈34 %); Elemental analysis calcd (%) for C_40_H_58_Ce_2_N_16_ (1042.25 g mol^−1^): C 46.05, H 5.60, N 21.48; found: C 46.14, H 5.54, N 20.99. It should be noted that adding [Ce(N*i*Pr_2_)_4_] to a solution of Me_2_pzH also did not give **1** and reduction ensued in a similar manner as above.

### Investigations into Si−H bond activation

**Reaction between [Ce{N(SiHMe_2_)_2_}_4_] and three equivalents of Me_2_** **pzH**: [Ce{N(SiHMe_2_)_2_}_4_] (13.5 mg, 0.02 mmol) and Me_2_pzH (6.0 mg, 0.06 mmol) were combined and shaken in [D_6_]benzene and analyzed immediately by ^1^H NMR spectroscopy. The ^1^H NMR spectrum indicated decomposition after several minutes of stirring (see Figure S6).

**Reaction between [Ce^III^{N(SiHMe_2_)_2_}_3_(thf)_2_] and three equivalents of Me_2_** **pzH**: [Ce{N(SiHMe_2_)_2_}_3_(thf)_2_] (72.0 mg, 0.11 mmol) and Me_2_pzH (30.1 mg, 0.32 mmol) were combined in an NMR tube. [D_8_]THF (≈0.1 mL) and 0.4 mL of [D_6_]benzene were added, dissolving the solids. The pale‐yellow solution was analyzed by ^1^H NMR spectroscopy after either 5 minutes or 30 minutes, showing no differences, and exclusive formation of [Ce(Me_2_pz)_3_] and HN(SiHMe_2_)_2_. ^1^H NMR ([D_6_]benzene, 300 K, 400 MHz): *δ*=11.39 ppm (br s, ≈3 H, Me_2_pz‐C*H*), 6.08 (br s, ≈18 H, Me_2_pz‐C*H*_3_), 4.58 (m, 6 H, Si−H), 0.14 (br s, 36 H, Si‐C*H*_3_) ppm. After 3 d at ambient temperature, or 2 d at 60 °C, no changes were observed by ^1^H NMR spectroscopy. After evaporation of the reaction mixture to dryness, addition of [D_8_]toluene, and addition of [Li{N(SiHMe_2_)_2_}] (14.0 mg, 0.10 mmol), a complex spectrum was obtained but did not change over time. Storing this reaction mixture at ambient temperature for several weeks, followed by addition of *n*‐hexane and storage at −35 °C, crystallization from this reaction mixture gave a mixture of powder and colorless crystals. Some crystals were handpicked and analyzed by single‐crystal X‐ray crystallography giving the structure of [Li_2_(thf)_2_Ce(Me_2_pz)_2_{N(SiHMe_2_)_2_}_3_] (**5**); approximate yield after separation (15.2 mg, 51 %, from handpicked crystals). Elemental analysis calcd (%) for: C_30_H_72_CeLi_2_N_7_O_2_Si_6_ (885.46 g mol^−1^); C 40.69, H 8.20, N 11.07; found: C 40.48, H 7.44, N 11.71.

**Reaction between [Ce(Me_2_** **pz)_4_] and HN(SiHMe_2_)_2_ in [D_6_]benzene at ambient temperature**: [Ce(Me_2_pz)_4_]⋅^1^/_2_PhMe (**1**⋅^1^/_2_PhMe, 44.0 mg, 0.08 mmol) and HN(SiHMe_2_)_2_ (≈5.0 mg, 0.04 mmol) were combined in [D_6_]benzene and periodically monitored by ^1^H NMR spectroscopy over the course of four months at ambient temperature (refer to Figure S2 for spectrum). After four months, FeCp_2_ (1.0 mg, 0.01 mmol) was added to determine the yield of **3** as approximately 3 %.

**Reaction between [Ce(Me_2_** **pz)_4_] and HN(SiHMe_2_)_2_ in [D_8_]toluene at elevated temperatures**: [Ce(Me_2_pz)_4_]⋅^1^/_2_PhMe (**1**⋅^1^/_2_PhMe, 10.4 mg, 0.02 mmol), FeCp_2_ (1.3 mg, 0.01 mmol), and HN(SiHMe_2_)_2_ (5.3 g, 0.04 mmol), were combined in [D_8_]toluene and heated at 80 °C for 3 h. No change was noted in the ^1^H NMR spectrum. The temperature was increased to 105 °C, and after 5 min decomposition was noted by the formation of small peaks assignable to paramagnetic species. After 16 h, the reaction was cooled and analysis by ^1^H NMR spectroscopy indicating complete consumption of **1** (refer to Figure S3). Small colorless crystals were observed within the NMR tube, and analysis by X‐ray crystallography revealed the formation of [Ce_4_(Me_2_pz)_12_(Me_2_pzH)_2_] (**2 c**). The crystals were insoluble in [D_8_]toluene even upon heating.

**Reaction between [Li{N(SiHMe_2_)_2_}] and [Li(Me_2_** **pz)]**: [Li{N(SiHMe_2_)}] (0.114 g, 0.82 mmol, 3 equiv) and Me_2_pzH (52.6 mg, 0.54 mmol, two equiv) were stirred in toluene (5 mL) for 1–2 h. The reaction mixture was evaporated to dryness, toluene was re‐added, and the sample was heated to 115 °C for two d. The sample was evaporated to dryness leaving a white powder. Analysis by ^1^H NMR spectroscopy indicated only slight changes in the spectrum of [Li(Me_2_pz)] and [Li{N(SiHMe_2_)_2_}] (refer to Figure S33).

### Reaction of [Ce(Me_2_ pz)_4_]_2_ (1) with lithium reagents: [LiCe_2_(Me_2_ pz)_9_] (6)

*Method a, Li impurity in the initial synthesis of **1***: Me_2_pzH (30.0 mg, 0.3 mmol)) was dissolved in toluene and added to a toluene solution of [Ce{N(SiHMe_2_)_2_}_4_] (52.2 mg, 0.08 mmol, note: reaction mixture contained an unknown amount of a Li impurity from initial synthesis of [Ce{N(SiHMe_2_)_2_}_4_]). The solution was immediately exposed to vacuum, and after 30 seconds it was stored at −35 °C, producing dark red block crystals of [LiCe_2_(Me_2_pz)_9_] (**6**) identified by X‐ray crystallography. The crystals were briefly dried under vacuum (18.0 mg, 40 %). DRIFT: *v̄*=3100 (vw), 2951 (w), 2920 (m), 2862 (w), 1565 (vw), 1521 (vs), 1460 (m), 1434 (s), 1413 (vs), 1376 (m), 1365 (w), 1328 (w), 1309 (s), 1264 (m), 1057 (w), 1021 (m), 1012 (m), 965 (m), 821 (vw), 797 (w), 787 (w), 762 (m), 740 (vw), 729 (m), 694 (vw) cm^−1^. Elemental analysis calcd (%) for C_52_H_71_Ce_2_LiN_18_ (1235.41 g mol^−1^): C 50.55, H 5.79, N 20.41; found: C 50.52, H 5.24, N 20.69. The crystals were further dried under vacuum before ^1^H NMR analysis, removing the lattice toluene (assignments are provided in Figures S10 and S11). ^1^H NMR ([D_6_]benzene, 300 K, 250 MHz): *δ*=6.00 (s, 4 H, pz‐C(4)*H*), 5.76 (s, 1 H, pz‐C(4)*H*), 5.73 (s, 2 H, pz‐C(4)*H*), 4.75 (s, 2 H, pz‐C(4)*H*), 2.65 (s, 6 H, C*H*_3_), 2.17 (s, 12 H, C*H*_3_), 1.98 (s, 24 H, C*H*_3_), 1.95 (s, 12 H, C*H*_3_) ppm; ^13^C{^1^H} NMR ([D_6_]benzene, 300 K, 400 MHz): *δ*=165.1, 145.0, 144.8, 143.6, 113.3, 105.8, 100.7, 14.5, 13.4, 12.81, 12.77 ppm; χ_mol_=−2.192×10^−4^ cm^3^ mol^−1^, *μ*_eff_=≈0.0 BM (Δ*H*z: −4.3, 2×10^−3^ mol L^−1^); UV(toluene): *λ*_max_=423 (*ϵ*=10 400 mol^−1^ cm^−1^).

*Method b, protonolysis with [Ce(Me_2_pz)_4_(Me_2_pzH)]⋅^1^/_2_PhMe*: [Ce(Me_2_pz)_4_(Me_2_pzH)]⋅^1^/_2_PhMe (7.0 mg, 0.01 mmol) and [Li{N(SiHMe_2_)_2_}] (2.0 mg, 0.01 mmol) were combined in [D_8_]THF and stored for 12 h. *n*‐Hexane was added, and upon storage at −35 °C, crystals of [LiCe_2_(Me_2_pz)_9_] (**6**) formed and were identified by ^1^H NMR spectroscopy (Figure S14.2); the spectrum was in accordance with the sample obtained by method a (crystal yield: 5.0 mg, 51 %).

**Reaction between [Ce(Me_2_** **pz)_4_] and [Li{N(SiHMe_2_)_2_}]**: [Ce(Me_2_pz)_4_]⋅^1^/_2_PhMe (22.0 mg, 0.0421 mmol) and [Li{N(SiHMe_2_)_2_}] (45.0 mg, 0.03 mmol) were combined in [D_6_]benzene giving a deep red solution. The initial ^1^H NMR spectrum indicated the following species were present in solution: [Li(Me_2_pz)], [LiCe_2_(Me_2_pz)_9_] (**6**), [Ce(Me_2_pz)_3_(bpsa)] (**3**), and two different species containing “Ce{N(SiHMe_2_)_2_}“ moieties (see Figure S12.1–12.3). After two days, the reaction mixture showed complete conversion to multiple metal species (see Figure S13).

**Reaction between [Ce(Me_2_** **pz)_4_] and [Li{N(SiMe_3_)_2_}]**: [Ce(Me_2_pz)_4_]⋅^1^/_2_PhMe (11.3 mg, 0.02 mmol) and [Li{N(SiMe_3_)_2_}] (3.5 mg, 0.02 mmol) were combined in [D_6_]benzene giving a deep red solution. ^1^H NMR analysis indicated the presence of [Li{N(SiMe_3_)_2_}], [Li(Me_2_pz)], [LiCe_2_(Me_2_pz)_9_] (**6**), and [Ce(Me_2_pz)_2_{N(SiMe_3_)_2_}_2_] (**7**) (see Figures S14.1–14.3 and S15).

**Deliberate synthesis of [Ce(Me_2_** **pz)_2_{N(SiMe_3_)_2_}_2_] (7)**: [Ce{N(SiMe_3_)_2_}_3_] (150.8 mg, 0.24 mmol) and [Li{N(SiMe_3_)_2_}] (40.6 mg, 0.25 mmol) were stirred for 10 minutes in *n*‐hexane (≈2–5 mL) prior to addition of Me_2_pzH (46.7 mg, 0.486 mmol), dissolved in toluene (2 mL). The reaction was stirred for additional 10 minutes before 1,4‐benzoquinone (13.0 mg, 0.28 mmol) was added. The solution turned to dark brown/red. After one minute of stirring the reaction mixture was filtered, concentrated to approximately 1–2 mL, and stored at −35 °C, giving large red block crystals of [Ce(Me_2_pz)_2_{N(SiMe_3_)_2_}_2_] (**7**) (43.4 mg, 27 % crystal yield). ^1^H NMR ([D_6_]benzene, 300 K, 400 MHz): *δ*=6.21 (s, 2 H, C*H*), 2.44 (s, 12 H, C*H*_3_), 0.29 (s, 36 H, Si(C*H*_3_)_3_) ppm; ^13^C{^1^H} NMR ([D_6_]benzene, 100 MHz, 300 K): *δ*=145.63 (*C*(4)), 114.09 (*C*(3/5)), 13.6 (Me_2_pz‐*C*H_3_), 3.5 (Si(*C*H_3_)_3_) ppm; UV(toluene): *λ*_max_=430 (*ϵ*=3800 mol^−1^ cm^−1^). DRIFT: *v̄*=3103 (vw), 2949 (m), 2893 (w), 1539 (vw), 1517 (s), 1471 (w), 1455 (m), 1432 (s), 1366 (w), 1311 (w), 1297 (w), 1247 (vs), 1100 (vw), 1053 (vw), 1003 (w), 935 (vs), 837 (vs), 795 (m), 775 (m), 756 (m), 725 (w), 680 (w), 654 (w), 609 (s) cm^−1^. χ_mol_=4.38×10^−4^ cm^3^ mol^−1^, *μ*_eff_=1.02 BM (Δ*H*z: 6, 0.0614 mol L^−1^). Elemental analysis calcd (%) for C_22_H_50_CeN_6_Si_4_ (651.13 g mol^−1^): C 40.58, H 7.74, N 12.91; found: C 40.76, H 7.64, N 12.91.

**Isolation of [{Li_2_Ce_2_(Me_2_** **pz)_6_(thf)_2_}_2_(pzhq)_2_] (8) from the reaction between 1,4‐benzoquinone and “Li[Ce(Me_2_** **pz)_4_]”**: [Ce{N(SiMe_3_)_2_}_3_] (154.3 mg, 0.25 mmol), Li(N(SiMe_3_)_2_) (28.0 mg, 0.2 mmol), and Me_2_pzH (76.9 mg, 0.8 mmol) were stirred in THF for 16 h. The solution was dried in vacuo, and the resulting powder dissolved in toluene and stored at −35 °C. 1,4‐Benzoquinone (13.0 mg, 0.12 mmol) was added at −35 °C, giving an immediate Bordeaux‐colored solution. The solution was stirred for 60 seconds before storage at −35 °C for 12 h. The solution was filtered, concentrated in vacuo, and *n*‐hexane was added giving a colorless powder and Bordeaux‐colored crystals. X‐ray structural analysis of the crystals indicated the formation of [{Li_2_Ce_2_(Me_2_pz)_6_(thf)_2_}_2_(pzhq)_2_] (**8**).

**Reaction between [Ce(Me_2_** **pz)_4_] and [Ce{N(SiHMe_2_)_2_}_4_]**: [Ce(Me_2_pz)_4_]_2_⋅^1^/_2_PhMe (0.0080 g, 0.0077 mmol) and [Ce{N(SiHMe_2_)_2_}_4_] (0.010 g, 0.015 mmol) were combined in C_6_D_6_ giving a deep red solution. The initial ^1^H NMR spectrum indicated no reaction. After one day, the reaction mixture showed decomposition of [Ce(Me_2_pz)_4_]_2_ and evolution of H_2_ (see Figure S34).

**Reaction between [Ce(Me_2_** **pz)_4_] and H_2_**: [Ce(Me_2_pz)_4_]_2_⋅^1^/_2_PhMe (8.4 mg, 8.1 μmol) was dissolved in C_6_D_6_ and treated with H_2_. The ^1^H NMR spectrum indicated no reaction after one day (see Figure S35).

**X‐ray crystallography**: Prior analysis, all compounds were submerged in *n*‐paratone crystallography oil, mounted on a fiber loop, and measured on a Bruker APEX‐II CCD′ diffractometer (Mo_Kα_, *λ*=0.71073 Å), at either 100 K (2 a*, **2 b**, **3**, **6**, **7**, **8**) or 161 K (**5**). Absorption corrections were completed using the Apex II program suite.^44^ Structural solutions were obtained by charge flipping methods or direct methods and refined using full matrix least squares methods against *F* ^2^ using SHELX2013,^45^ within the OLEX 2 graphical interface.^46^ A list of the parameters are depicted in Tables S1 and S2. Notes: 2 a*: ISOR command used on disordered carbon atoms of a THF ring and on NPD atom of a Me_2_pz ligand. **2 b**: Entire molecule was disordered over two positions. Metals and axial ligands were modelled over two positions with some atoms left isotropic, meridional ligands were not (attempted refinement with anisotropic atoms led to an unstable solution). ISOR command was used to restrain NPD atoms, and DFIX command was used to model partial disorder. N‐H hydrogen atom assignment was unstable and was not included in the model. **5**: Si−H hydrogen atoms were manually assigned. **6**: ISOR command used on disordered toluene molecule within lattice. Toluene was disordered over two positions, and on a special position, refined with PART refinement. **8**: One THF ligand, and one Me_2_pz ligand were disordered over several positions. ISOR command used on NPD carbon atoms (two THF carbon atoms remain isotropic), and the Me_2_pz ligand was modelled over two positions, attempts at modelling the THF ligand led to an unstable refinement. Highly disordered solvent within the lattice, identified as toluene and also potentially THF were removed through application of the SQUEEZE program suite in PLATON.^47^

Deposition Numbers 1540352 (2 a*), 1540356 (**2 b)**, 1858038 (**2 c)**, 1540356 (**3)**, 1858039 (**5)**, 1540351 (**6)**, 1540350 (**7)**, and 1540355 (**8)**   contain the supplementary crystallographic data for this paper. These data are provided free of charge by the joint Cambridge Crystallographic Data Centre and Fachinformationszentrum Karlsruhe Access Structures service www.ccdc.cam.ac.uk/structures. 

## Conflict of interest

The authors declare no conflict of interest.

## Supporting information

As a service to our authors and readers, this journal provides supporting information supplied by the authors. Such materials are peer reviewed and may be re‐organized for online delivery, but are not copy‐edited or typeset. Technical support issues arising from supporting information (other than missing files) should be addressed to the authors.

Supplementary
