# Supplementary material for: Emergence of a New [NNN] Pincer Ligand via Si−H Bond Activation and β‐Hydride Abstraction at Tetravalent Cerium
Source: Chemistry. 2020 Sep 7;26(53):12194–205. doi: 10.1002/chem.202000625 (PMC7540680; doi:10.1002/chem.202000625)
Supplement: Supplementary file 1 — Supplementary [file CHEM-26-12194-s001.pdf]

// Chemistry–A European Journal

Supporting Information

Emergence of a New [NNN] Pincer Ligand via Si–H Bond Activation and β-Hydride Abstraction at Tetravalent Cerium

Daniel Werner, Uwe Bayer, Dorothea Schädle, and Reiner Anwander*[a]

Table of Contents

Figure S1. ^{1}H NMR spectrum of [Ce{N(SiHMe$_2$)$_2$}$_4$] and 4 equiv. Me$_2$pzH........ S4

Figure S2. In situ ^{1}H NMR spectra of [Ce(Me$_2$pz)$_4$]$_2$ (**1**) + HN(SiHMe$_2$)$_2$. S5

Figure S3. ^{1}H NMR spectra of [Ce(Me$_2$pz)$_4$]$_2$ (**1**) and HN(SiHMe$_2$)$_2$ before and after thermal treatment S6

Figure S4. Molecular structure of [Ce$_4$(Me$_2$pz)$_{12}$(Me$_2$pzH)$_2$] (**2c**)........ S7

Figure S5. ^{1}H NMR spectrum of [Ce{N(SiHMe$_2$)$_2$}$_4$] and 5 equiv. Me$_2$pzH........ S8

Figure S6. ^{1}H NMR spectrum of the reaction between [Ce{N(SiHMe$_2$)$_2$}$_4$] and 3 equiv. Me$_2$pzH S9

Figure S7. ^{1}H NMR spectrum of the reaction between [Ce{N(SiHMe$_2$)$_2$}$_3$(thf)$_2$] and 3 equiv. Me$_2$pzH S10

Figure S8. ^{1}H NMR spectrum of the reaction mixture between [Ce(Me$_2$pz)$_3$(thf)$_2$] and [Li(N{SiHMe$_2$}$_2$)]........ S11

Figure S9. ^{1}H NMR spectrum of crystalline [Li$_2$(thf)$_2$Ce(Me$_2$pz)$_2${N(SiHMe$_2$)$_2$}$_3$] (**5**).. .... S12

Figure S10. ^{1}H NMR spectrum of [LiCe$_2$(Me$_2$pz)$_9$] (**6**). S13

Figure S11. ^{13}C NMR spectrum of [LiCe$_2$(Me$_2$pz)$_9$] (**6**). S14

Figure S12.1-12.3 ^{1}H NMR spectrum of the reaction between [Ce(Me$_2$pz)$_4$] (**1**) and [Li{N(SiMe$_3$)$_2$}] S15-17

Figure S13. ^{1}H NMR spectrum of [Ce(Me$_2$pz)$_4$] (**1**) and [Li{N(SiHMe$_2$)$_2$}] after two days S18

Figure S14.1-14.3 ^{1}H NMR spectrum of [Ce(Me$_2$pz)$_4$] (**1**) and [Li{N(SiMe$_3$)$_2$}]........ S19-S21

Figure S15. ^{1}H NMR spectrum of [Ce(Me$_2$pz)$_2${N(SiMe$_3$)$_2$}$_2$] (**7**)........ S22

Figure S16. ^{13}C NMR spectrum of [Ce(Me$_2$pz)$_2${N(SiMe$_3$)$_2$}$_2$] (**7**)........ S23

Figure S17. ^{1}H NMR spectrum of the reaction between excess [Ce(Me$_2$pz)$_2${N(SiMe$_3$)$_2$}$_2$] (**7**) and Me$_2$pzH. S24

Figure S18. Crystal structure of [Li$_4$Ce$_4$(Me$_2$pz)$_{10}$(Me$_2$pzH)$_4$(pzHq)$_2$]·2PhMe (**8**) S25

Figure S19. ^{1}H NMR spectra of [Ce(Me$_2$pz)$_2${N(SiMe$_3$)$_2$}$_2$] (7) + HN(SiHMe$_2$)$_2$ S26

Figure S20. Crystal structure of [Ce(Me$_2$pz)$_4$(Me$_2$pzH)] (**2b**) S27

Figure S21. ^{1}H NMR spectrum of [Ce(Me$_2$pz)$_4$(Me$_2$pzH)] (**2b**). S28

Figure S22. ^{13}C NMR spectrum of [Ce(Me$_2$pz)$_4$(Me$_2$pzH)] (**2b**). S29

Figure S23. ^{1}H NMR spectrum of [Ce(Me$_2$pz)$_4$(Me$_2$pzH)] (**2b**) at 173 K. S30

Figure S24. ^{1}H NMR spectrum of [Ce(Me$_2$pz)$_4$(thf)] (**2a**)........ S31

Figure S25. ^{13}C NMR spectrum of [Ce(Me$_2$pz)$_4$(thf)] (**2a**). S32

Figure S26. ^{1}H NMR spectrum of [Ce(Me$_2$pz)$_4$(thf)] (**2a**) at 173 K........ S33

Figure S27. ^{1}H NMR spectrum of [Ce(Me$_2$pz)$_3$(bpsa)] (**3**). S34

Figure S28. UV vis spectrum of [Ce(Me$_2$pz)$_4$(thf)] (**2a**) in toluene at ambient temperature. S35

Figure S29. UV vis spectrum of [Ce(Me$_2$pz)$_4$(Me$_2$pzH)] (**2b**)........ S35

Figure S30. UV vis spectrum of [Ce(Me$_2$pz)$_3$(bpsa)] (**3**) in *n*-hexane at ambient temperature........ S36

Figure S31. UV vis spectrum of [$LiCe_2(Me_2pz)_9$] (**6**) in toluene at ambient temperature. S36

Figure S32. UV vis spectrum of [$Ce(Me_2pz)_2\{N(SiMe_2H)_2\}_2$] (**7**) at ambient temperature in toluene S37

Figure S33. ^{1}H NMR spectrum of the reaction between 2 equiv. [$Li(Me_2pz)$] and [$Li\{N(SiHMe_2)_2)$])................ S38

Figure S34. ^{1}H NMR spectra of [$Ce(Me_2pz)_4]_2.{}^1/_2$PhMe + [$Ce\{N(SiHMe_2)_2\}_4$].. .... S39

Figure S35. ^{1}H NMR spectrum of [$Ce(Me_2pz)_4]_2.{}^1/_2$PhMe + H_2... .... S40

Table S1. Crystallographic parameters for complexes **2*-3**... S41

Table S2. Crystallographic parameters for complexes **5-8**.. S42

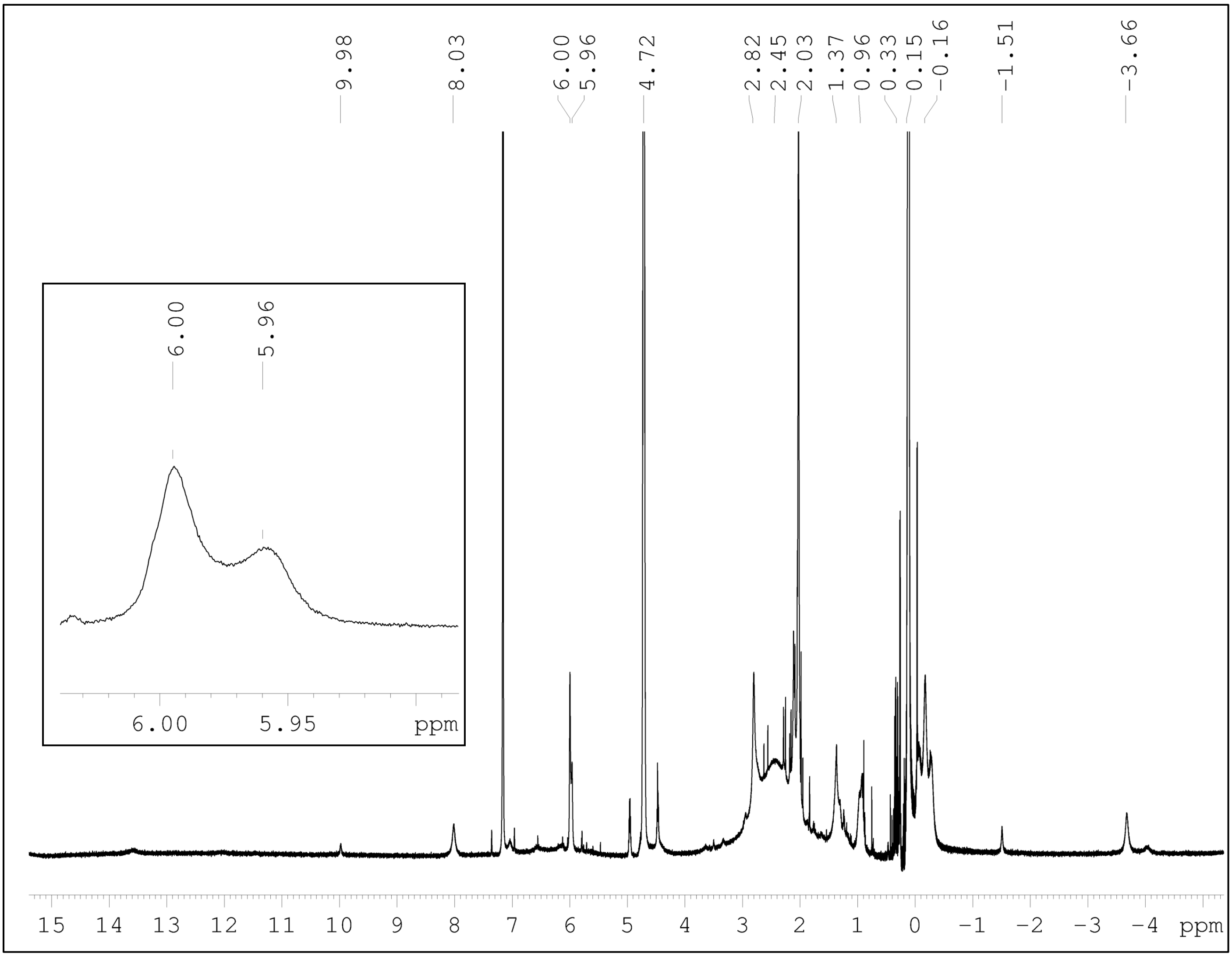

Figure S1. ^{1}H NMR ([D$_6$]benzene, 400 MHz, 300 K) spectrum of $[Ce\{N(SiHMe_2)_2\}_4]$ and 4 equiv. Me_2pzH. The sample was heated at 70 °C overnight to yield $[Ce(Me_2pz)_3(bpsa)]$ (**3**, also identified by the two resonances at 6.00 and 5.96 ppm) and other unidentified trivalent cerium complexes.

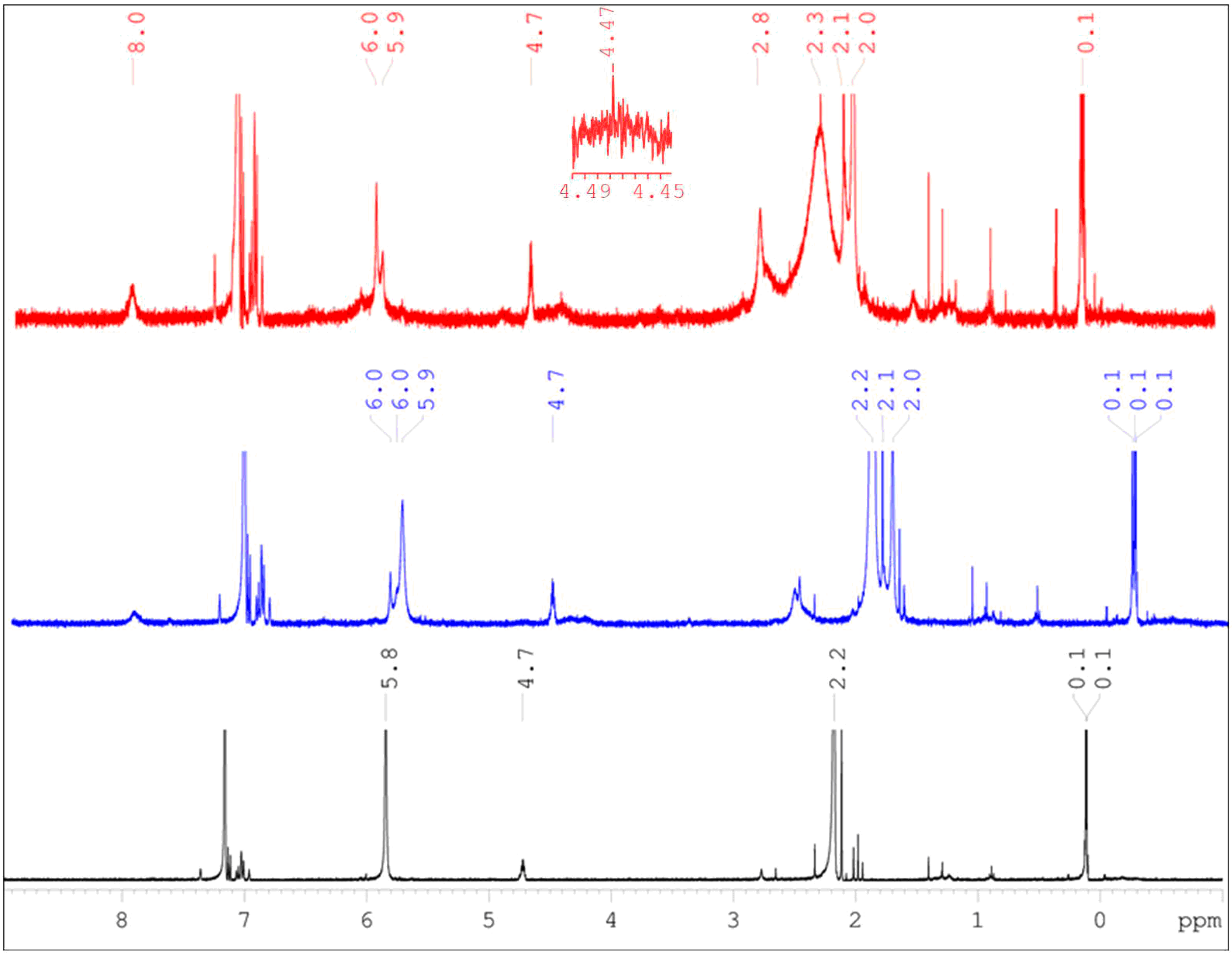

Figure S2. ^{1}H NMR ([D$_6$]benzene, 400 MHz, 300 K) spectra of [Ce(Me$_2$pz)$_4$]$_2$ (**1**) + HN(SiHMe$_2$)$_2$ at ambient temperature after: one day (black, bottom), one month (blue, middle), four months (red, top). To the final solution FeCp$_2$ was added as an internal standard to determine the yield of **3**.

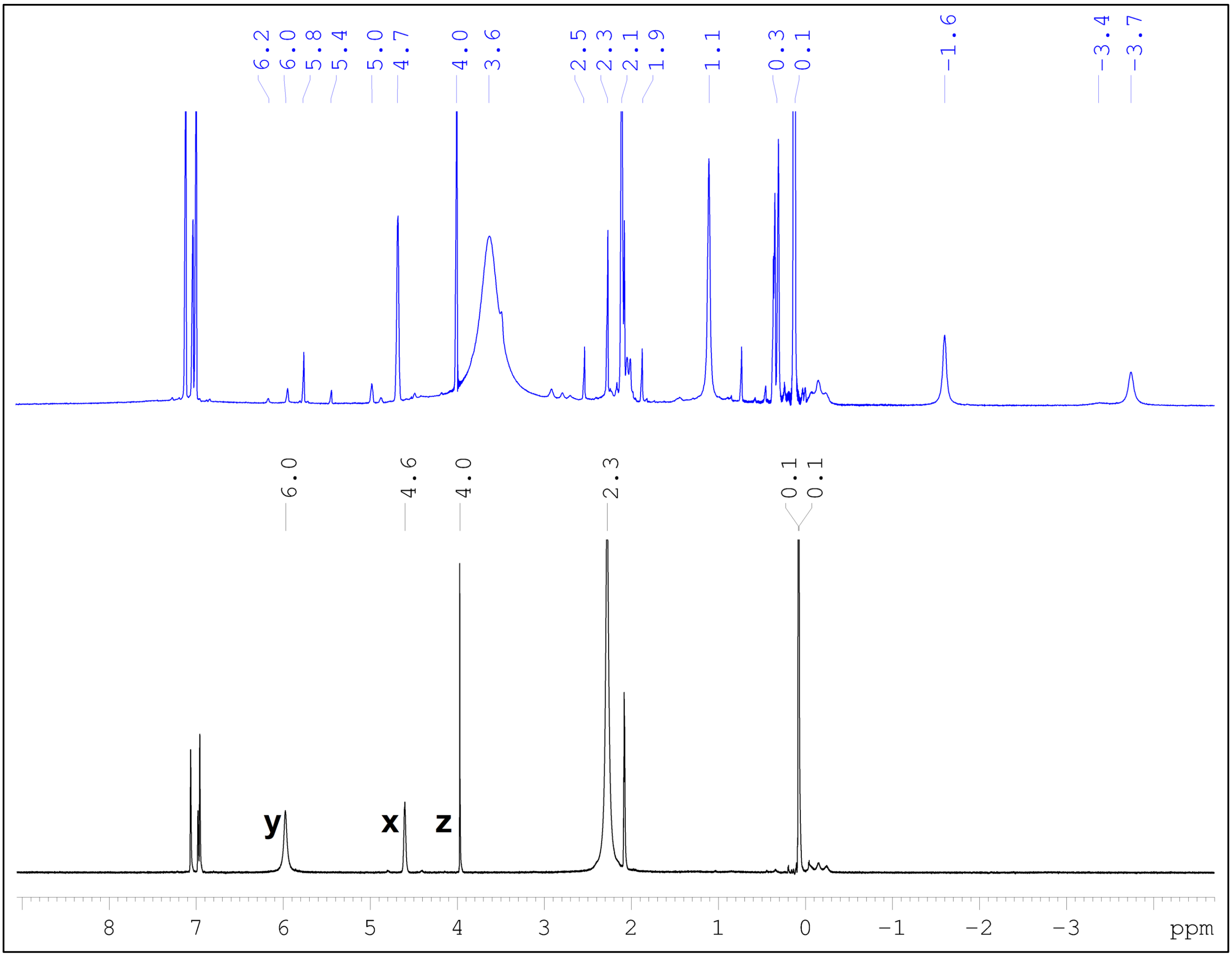

Figure S3. Bottom: ^{1}H NMR ([D$_8$]toluene, 500 MHz, 343 K) spectrum of [Ce(Me$_2$pz)$_4$]$_2$ (**1**) (**y**) + HN(SiHMe$_2$)$_2$ (**x**) and FeCp$_2$ (**z**). Top: ^{1}H NMR ([D$_8$]toluene, 500 MHz, 300 K) spectrum after heating at 80 °C (3 h) and then 105 °C (16 h). From this reaction mixture, single crystals of [Ce$_4$(Me$_2$pz)$_{12}$(Me$_2$pzH)$_2$] (**2c**) were isolated.

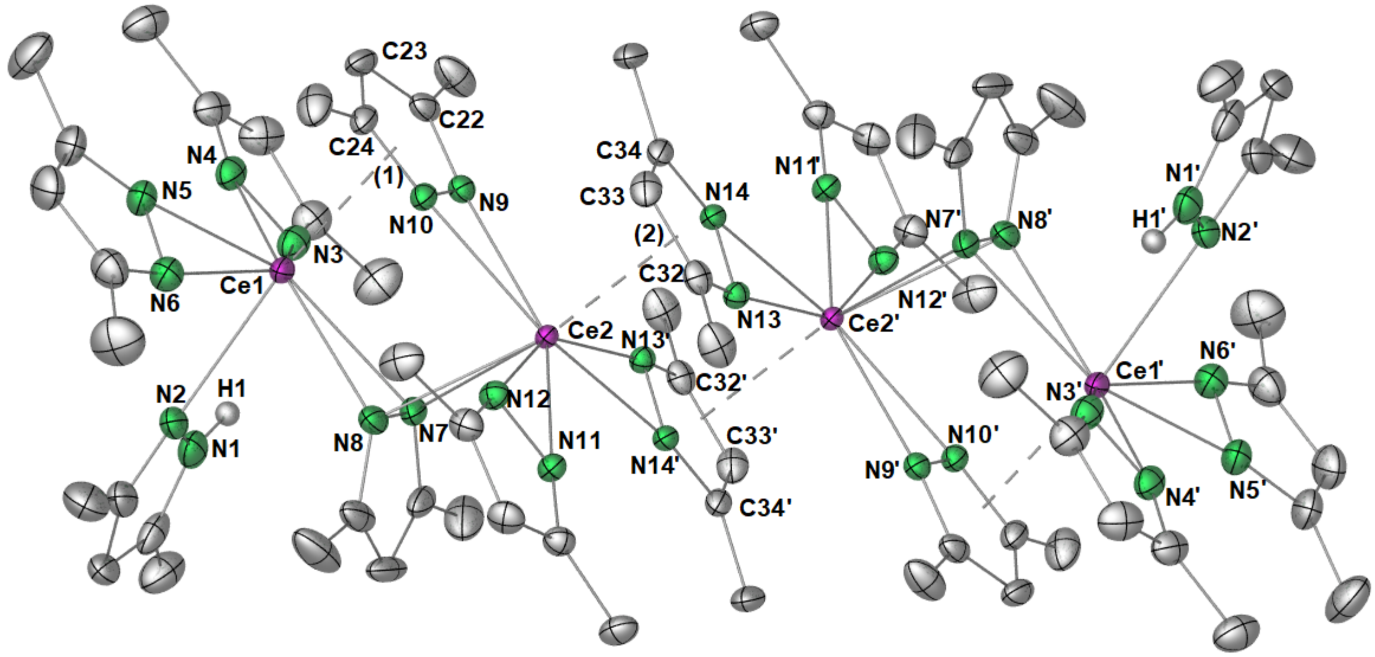

Figure S4. Crystal structure of [$Ce_4(Me_2pz)_{12}(Me_2pzH)_2$] (**2c**). Ellipsoids shown at 50% probability. Hydrogen atoms (with the exception of dimethylpyrazole H) are omitted for clarity. Selected bond lengths (Å) and angles (°): Ce1−Cent1 2.67610(8), Ce1−N2 2.6205(15), Ce1−N3 2.4943(16), Ce1−N4 2.4595(16), Ce1−N5 2.4533(15), Ce1−N6 2.5523(16), Ce1−N7 2.6457(14), Ce1−N8 2.6135(15), Ce1−N9 2.7240(13), Ce1−N10 2.7545(13), Ce1−C22 2.9665(17), Ce1−C23 3.1276(18), Ce1−C24 3.0120(17), Ce2−N7 2.7327(15), Ce2−N8 2.6726(15), Ce2−N9 2.5851(14), Ce2−N10 2.5500(13), Ce2−N11 2.4509(14), Ce2−N12 2.4717(14), Ce2−N13 2.7861(13), Ce2−N14 2.8014(13), Ce2−N13′ 2.5948(13), Ce2−N14′ 2.5491(14), Ce2−C32 3.0272(16), Ce2−C33 3.1601(18), Ce2−C34 3.0479(11), Ce2−Cent2 2.7280(8), Ce1−Cent1−Ce2 81.975(3) ; Ce2−Cent2−Ce2′ 84.139(2).

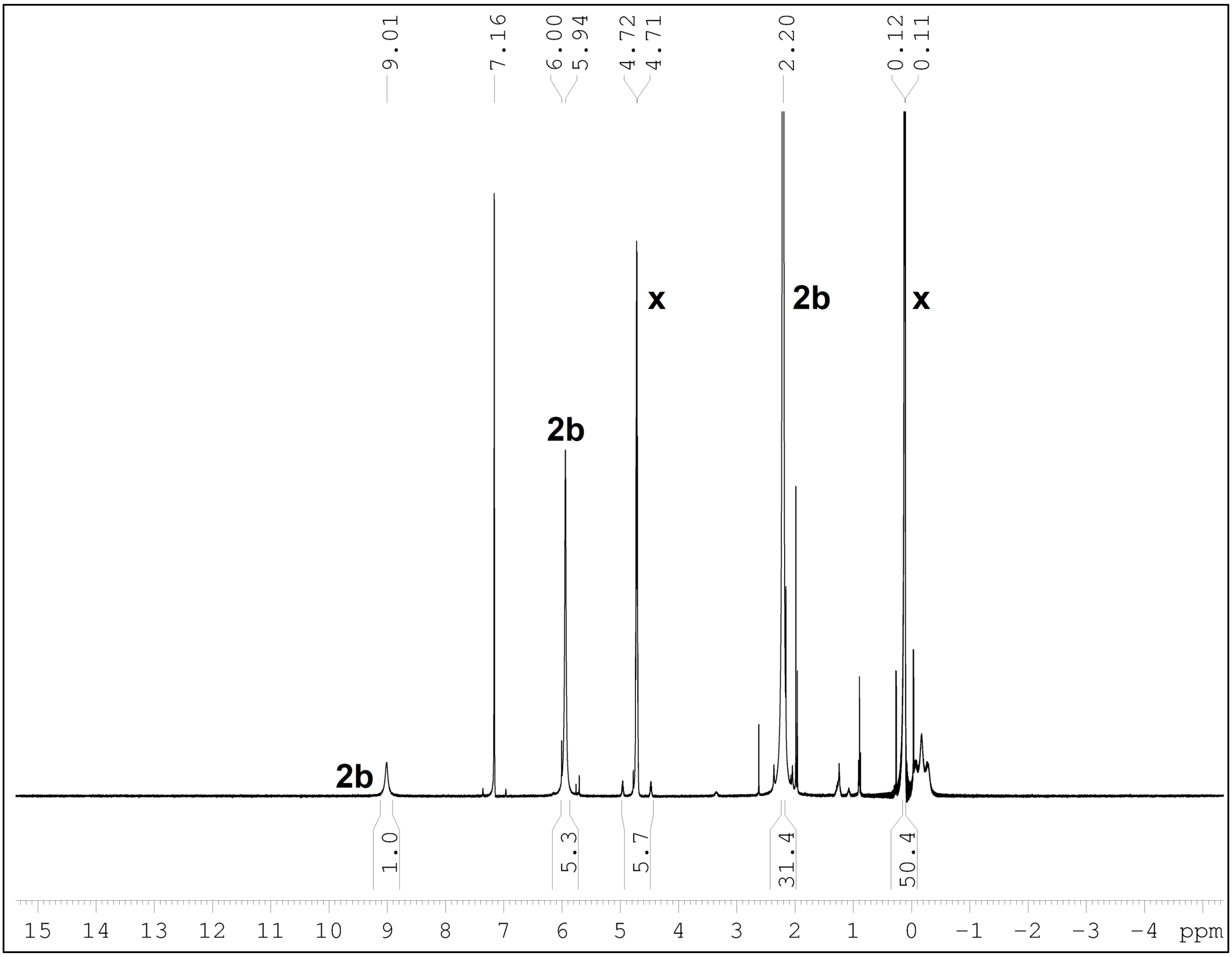

Figure S5. ^{1}H NMR ([D$_6$]benzene, 400 MHz, 300 K) spectrum of [Ce{N(SiHMe$_2$)$_2$}$_4$] and 5 equiv. Me$_2$pzH, giving [Ce(Me$_2$pz)$_4$(Me$_2$pzH)] (**2b**) and HN(SiHMe)$_2$ (**x**).

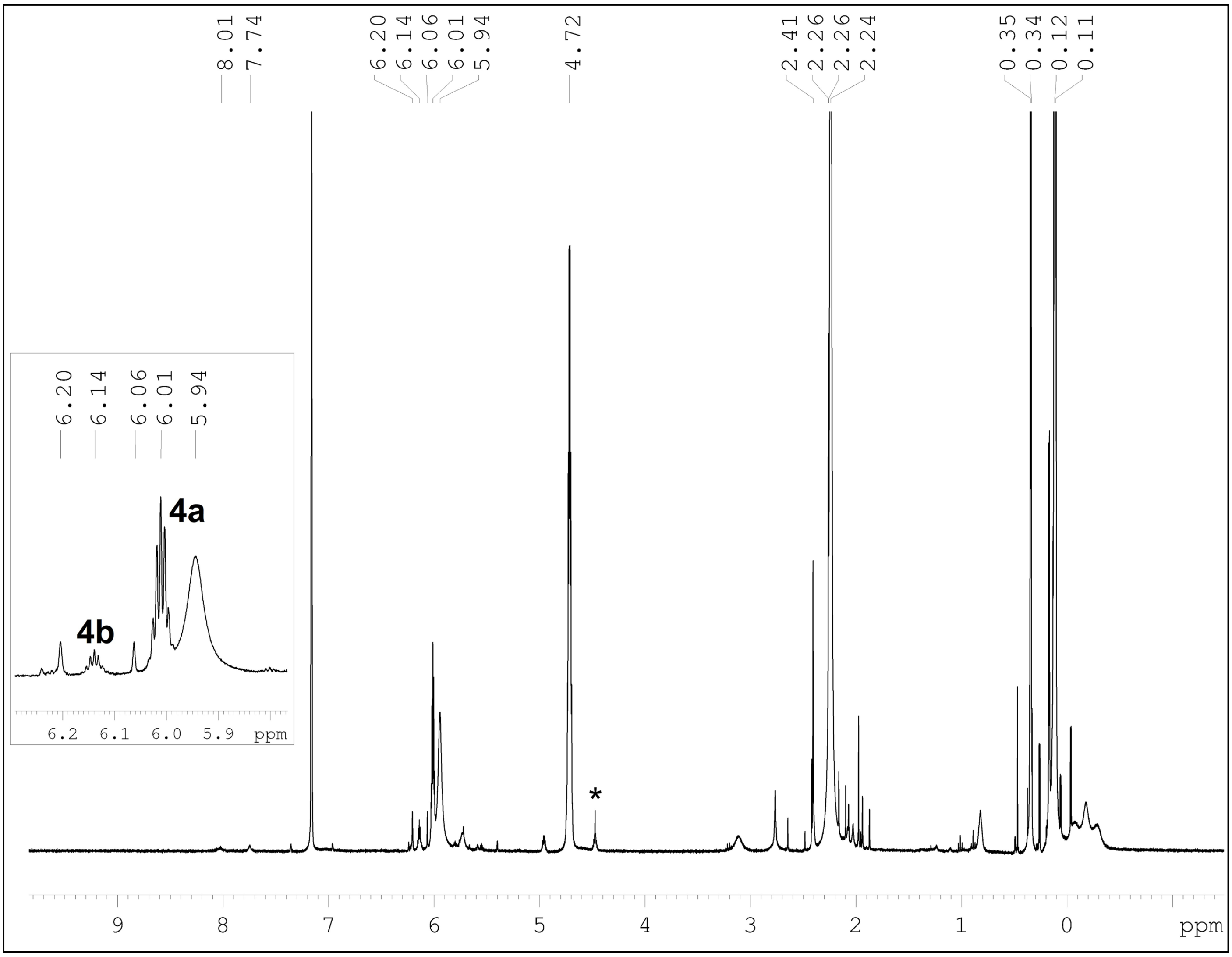

Figure S6. ^{1}H NMR ([D_6]benzene, 400 MHz, 300 K) spectrum of the reaction between [Ce{N(SiHMe$_2$)$_2$}$_4$] and 3 equiv. Me$_2$pzH after two minutes, indicating the onset of decomposition. The likely formation of intermediate [Ce(Me$_2$pz)$_2${N(SiHMe$_2$)$_2$}$_2$] (**4a**) and [Ce(Me$_2$pz)$_3${N(SiHMe$_2$)$_2$}] (**4b**) are indicated by the SiH septets (* the resonance at 4.47 ppm indicates the formation dihydrogen).

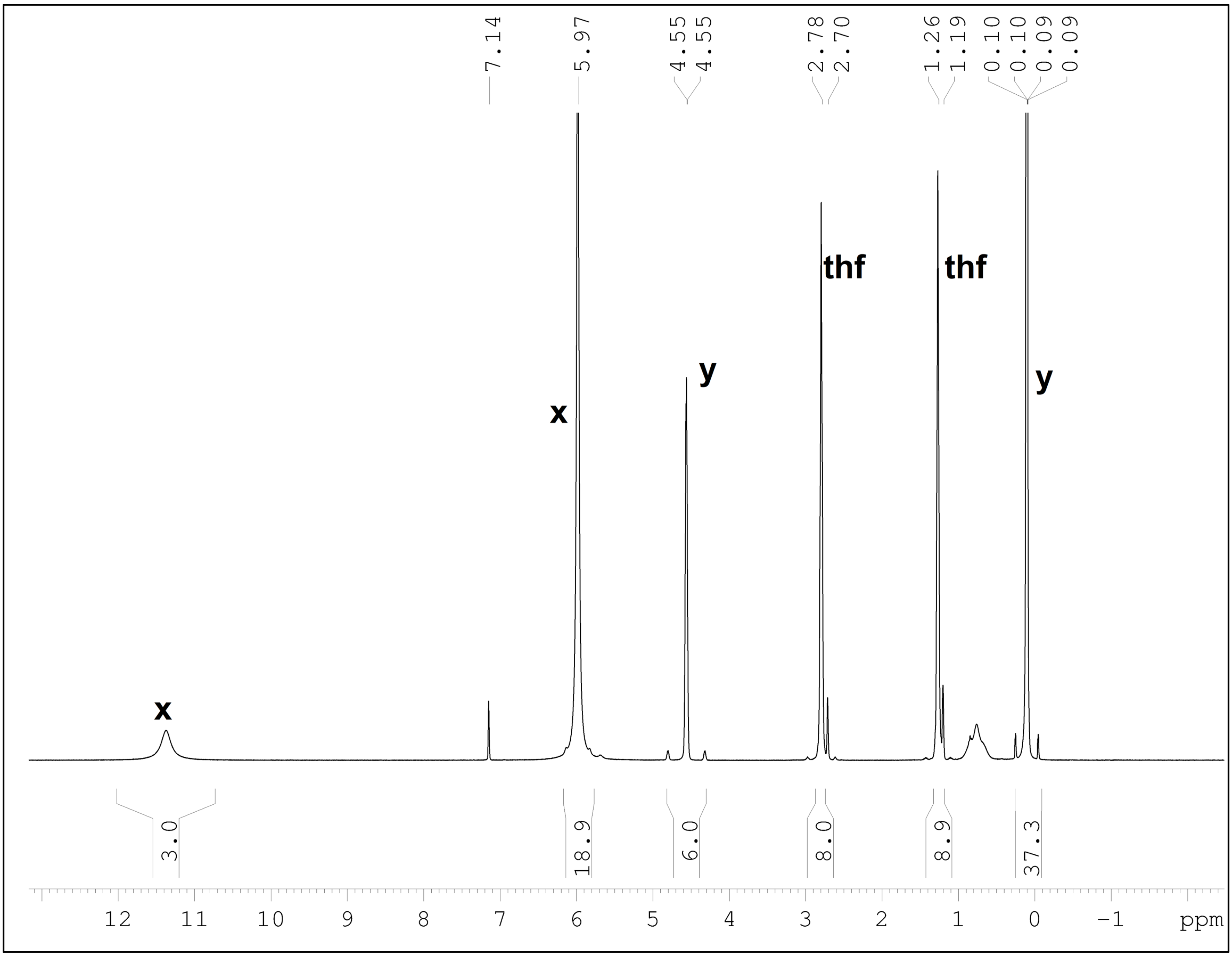

Figure S7. ^{1}H NMR ([D$_6$]benzene, 400 MHz, 300 K) spectrum of the reaction between [Ce{N(SiHMe$_2$)$_2$}$_3$(thf)$_2$] and 3 equiv. Me$_2$pzH after 30 minutes, giving [Ce(Me$_2$pz)$_3$] (**x**) and HN(SiHMe$_2$)$_2$ (**y**). No changes were observed in the spectrum over three days, or when heated at 60 °C for two days.

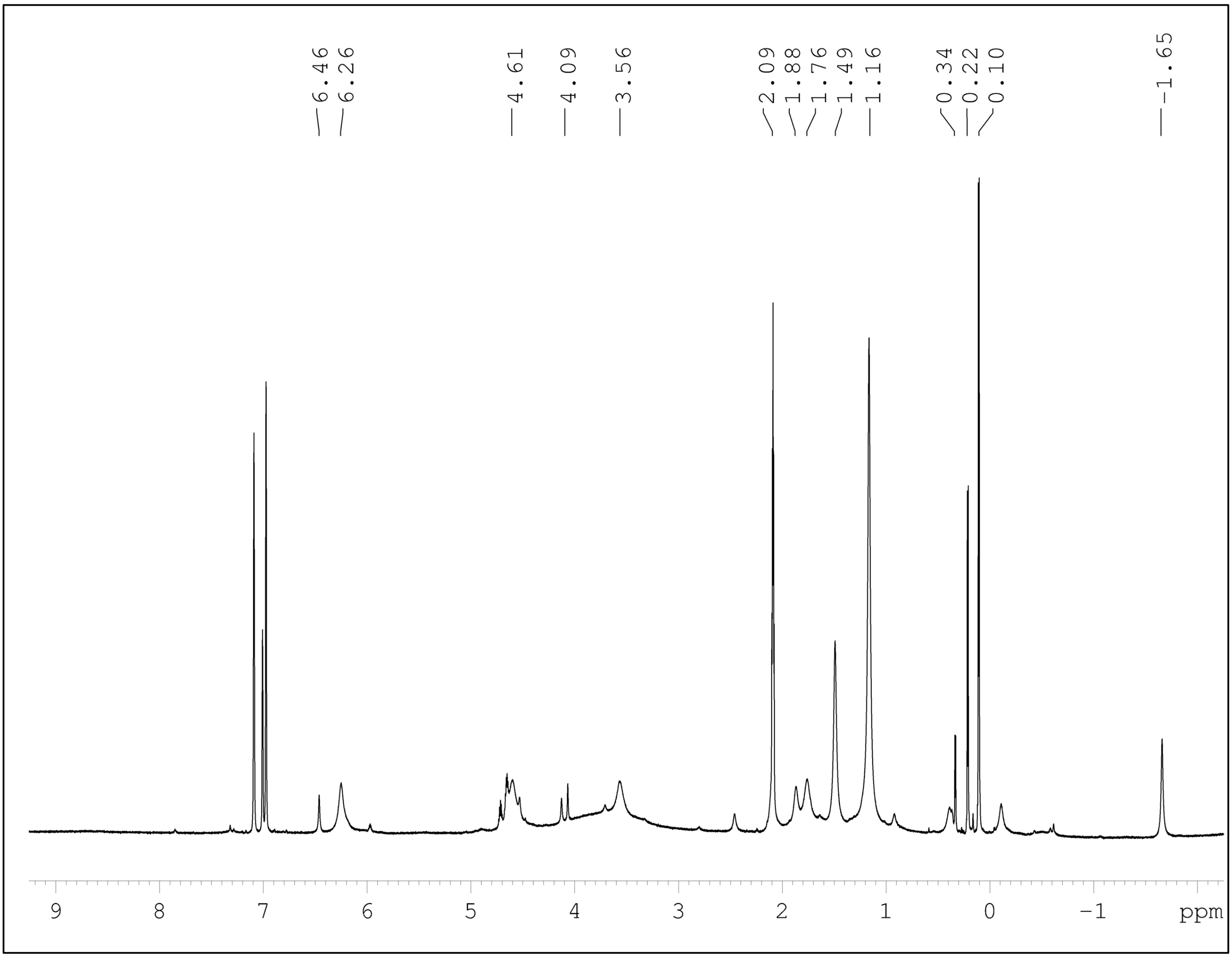

Figure S8. ^{1}H NMR ([D$_8$]toluene, 400 MHz, 300 K) spectrum of the reaction between [Ce(Me$_2$pz)$_3$(thf)$_2$] and [Li{N(SiHMe$_2$)$_2$}]. This reaction mixture did not change over time and after storage at −35 °C, single crystals of [Li$_2$(thf)$_2$Ce(Me$_2$pz)$_2${N(SiHMe$_2$)$_2$}$_3$] (**5**) were obtained.

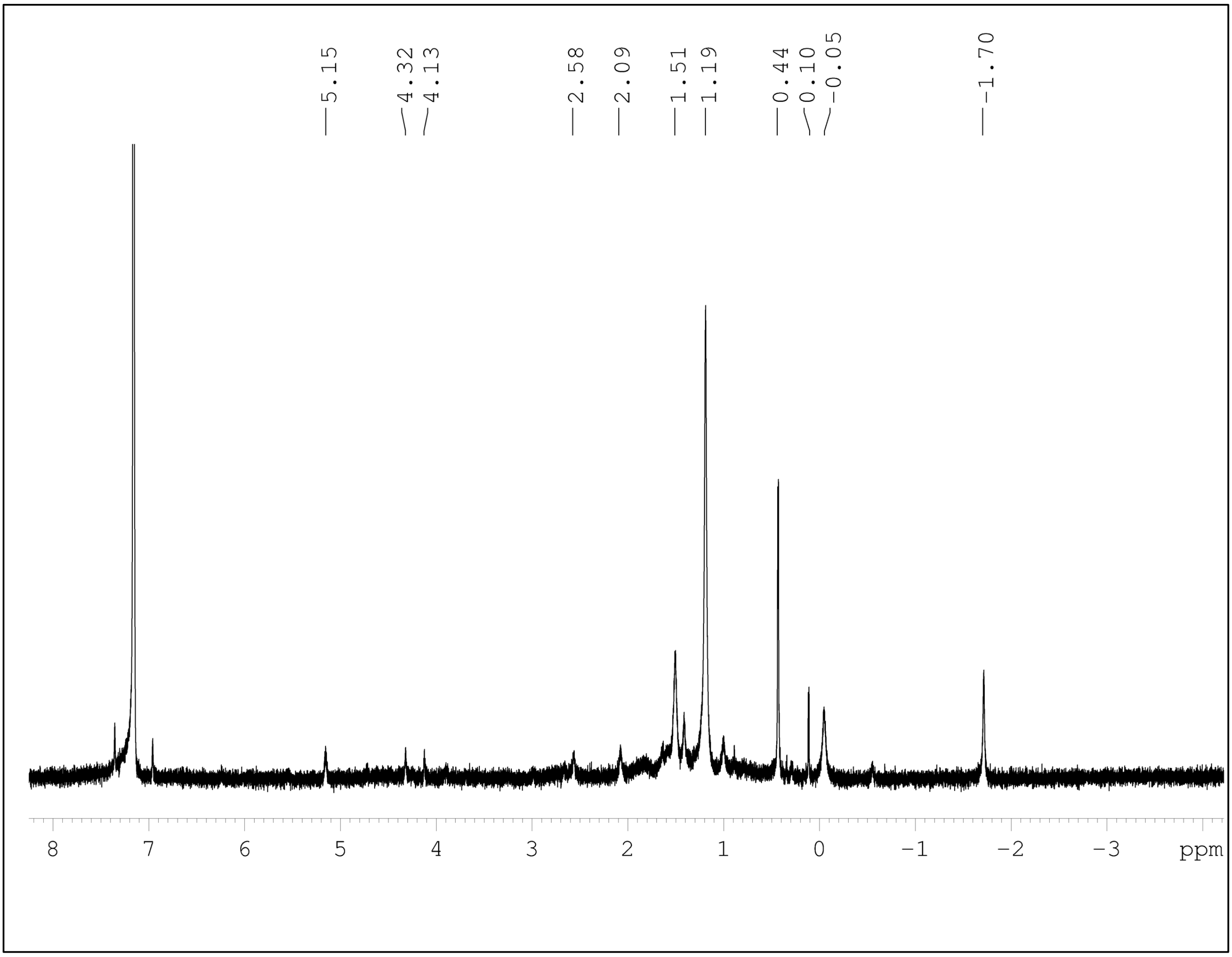

Figure S9. ^{1}H NMR ([D$_6$]benzene, 400 MHz, 300 K) spectrum of crystalline [$Li_2(thf)_2Ce(Me_2pz)_2\{N(SiHMe_2)_2\}_3$] (**5**) (crystals were sparingly soluble).

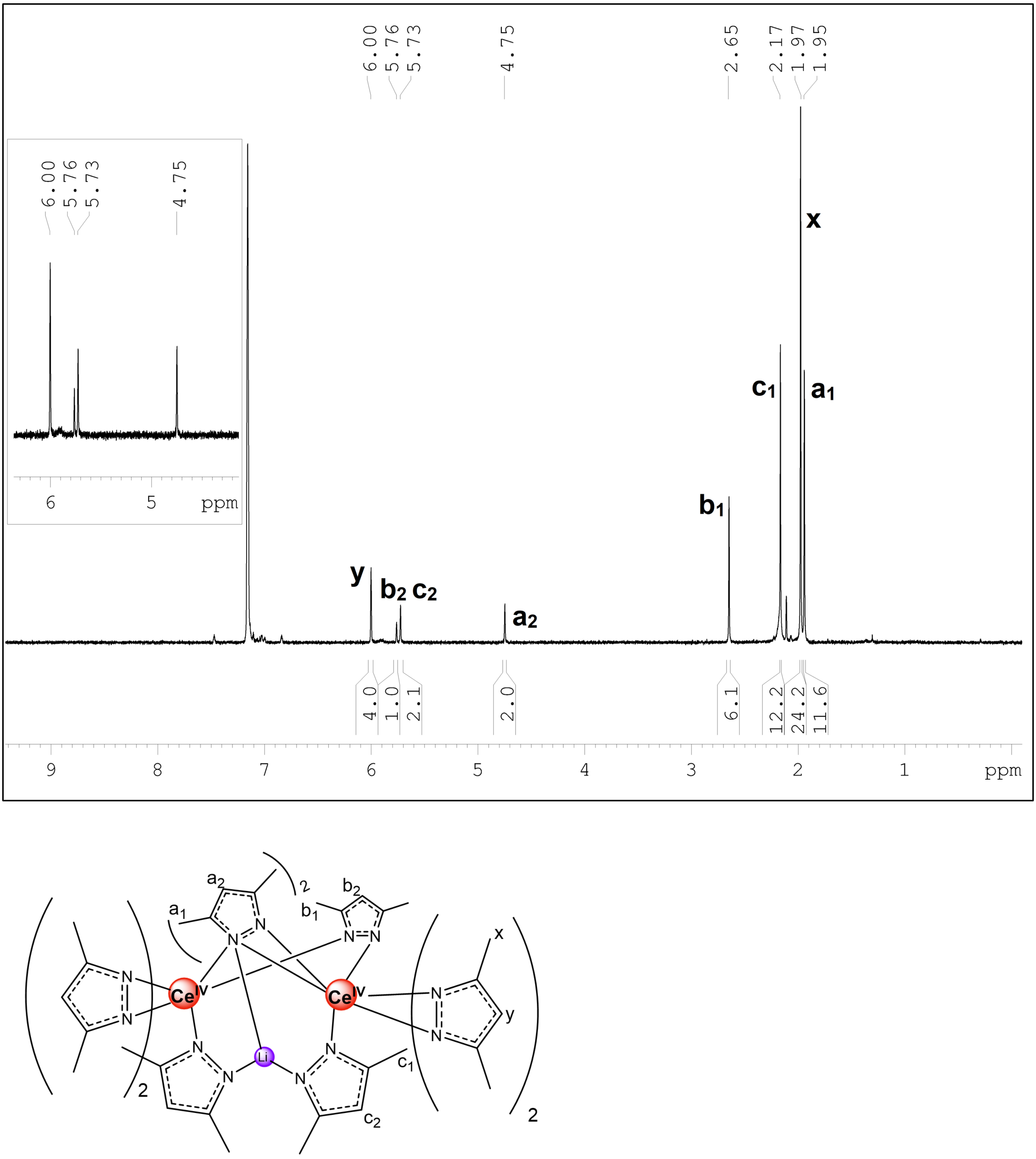

Figure S10. ^{1}H NMR ([D$_6$]benzene, 250 MHz, 300 K) spectrum of [LiCe$_2$(Me$_2$pz)$_9$] (**6**).

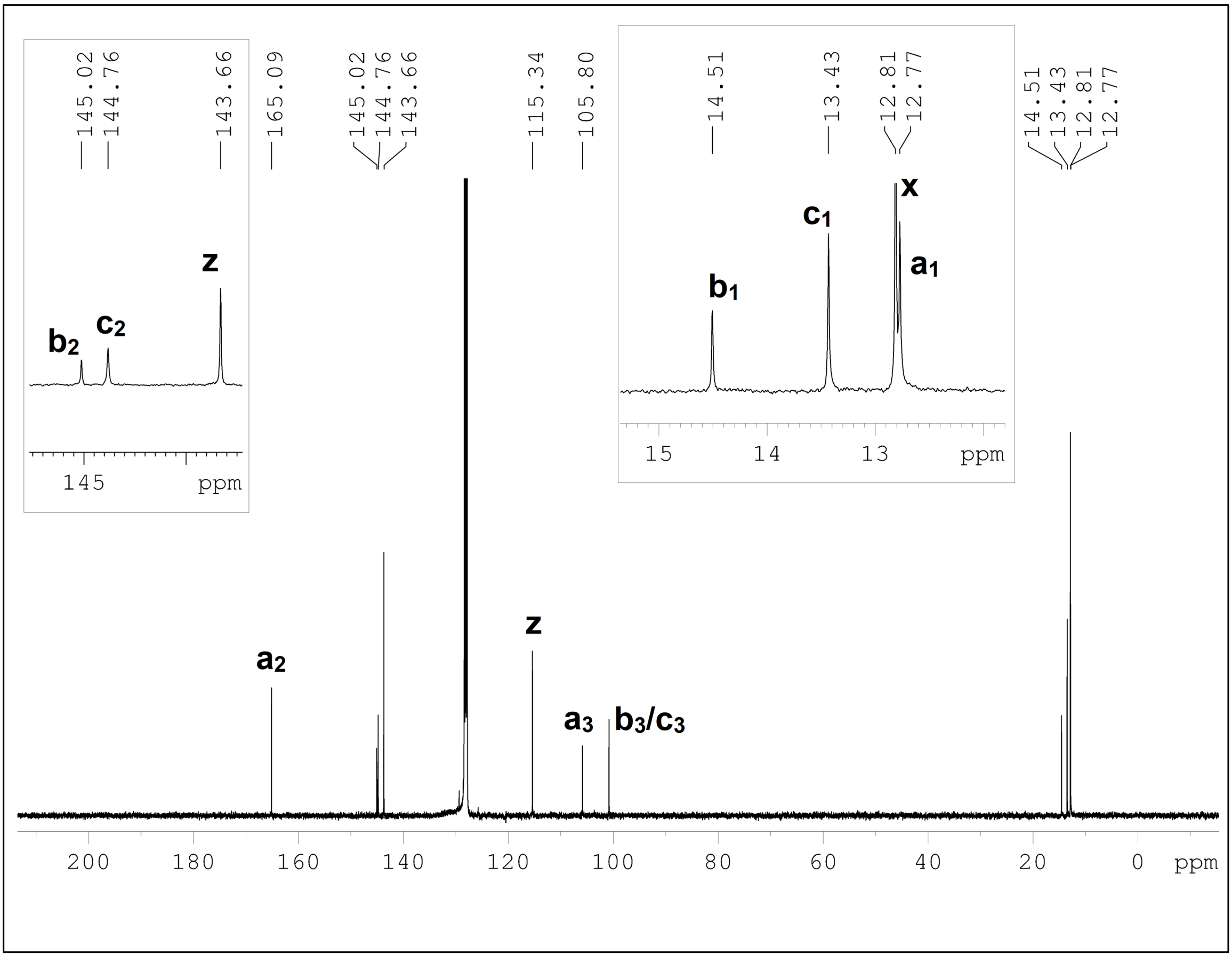

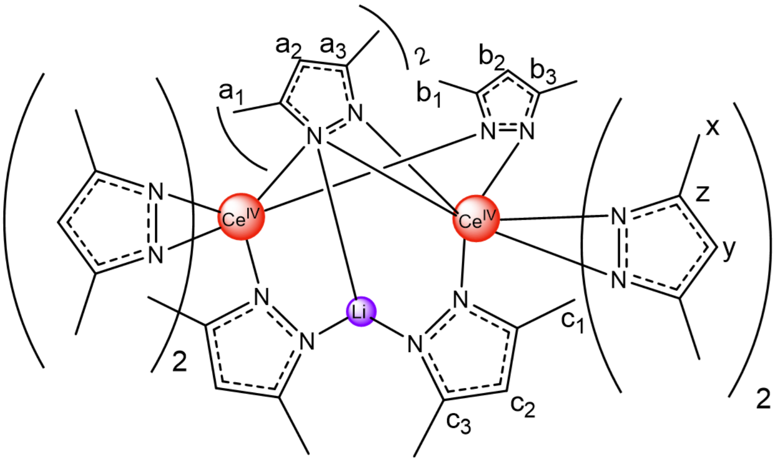

Figure S11. ^{13}C NMR ([D$_6$]benzene, 100.6 MHz, 300 K) spectrum of [LiCe$_2$(Me$_2$pz)$_9$] (**6**).

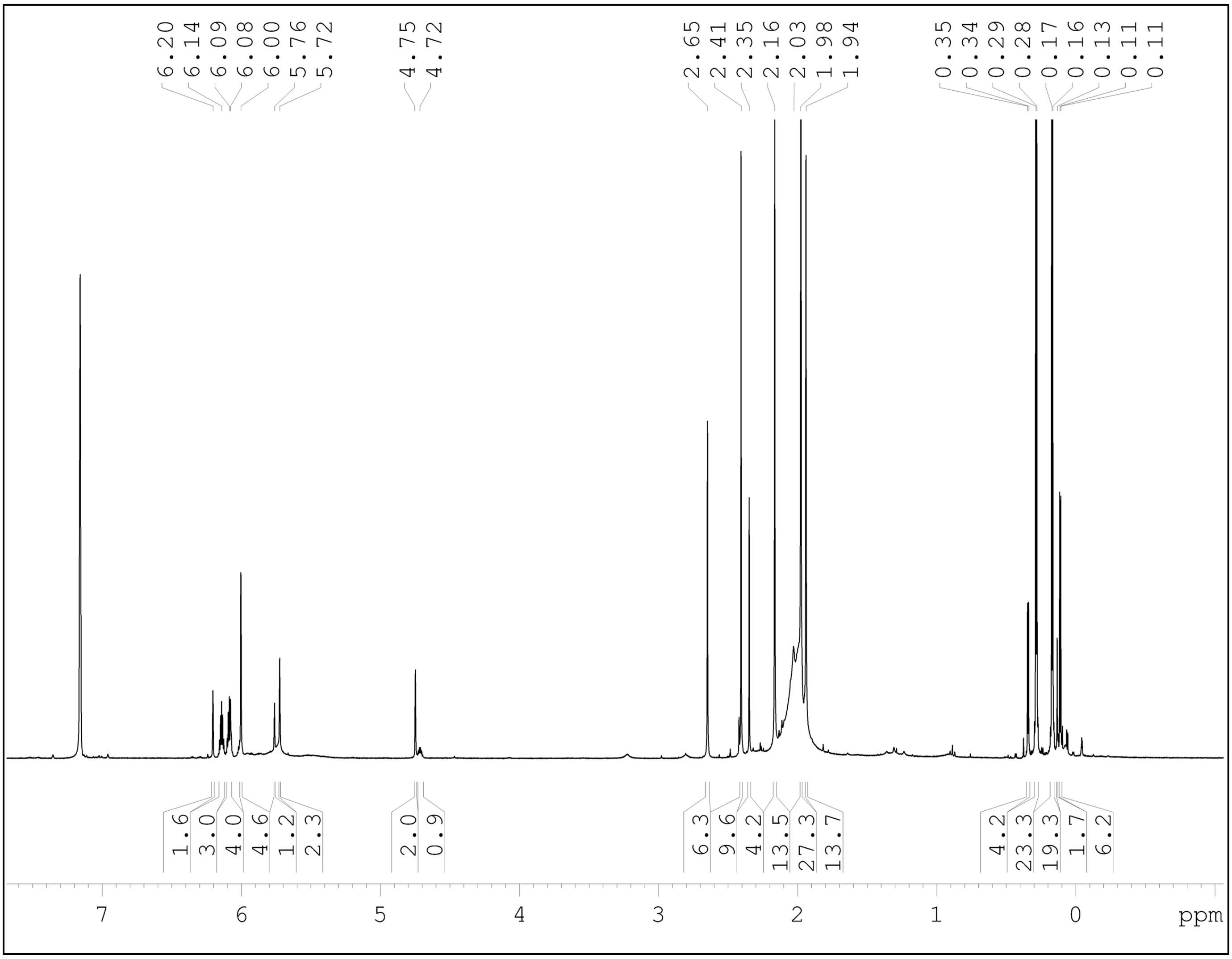

Figure S12.1 ^{1}H NMR ([D$_6$]benzene, 400 MHz, 300 K) spectrum of the reaction between [Ce(Me$_2$pz)$_4$]$_2$ (**1**) and [Li{N(SiHMe$_2$)$_2$}] after several minutes, see 12.2 and 12.3 for reactivity of present species.

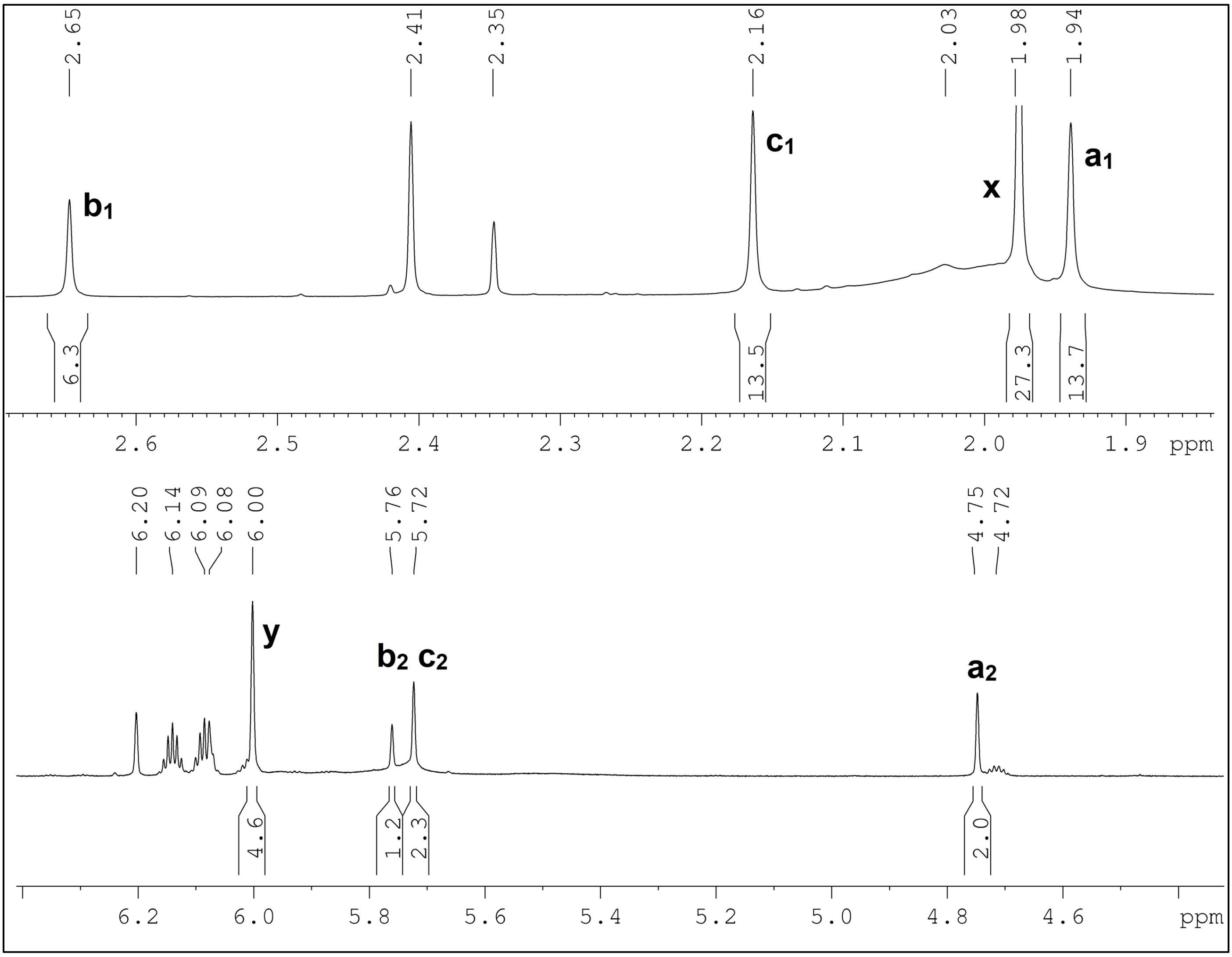

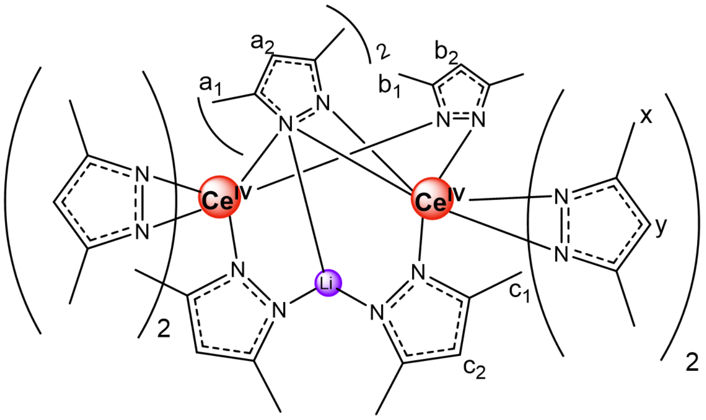

Figure S12.2 ^{1}H NMR ([D$_6$]benzene, 400 MHz, 300 K) spectrum of the reaction between [Ce(Me$_2$pz)$_4$]$_2$ (**1**) and [Li{N(SiHMe$_2$)$_2$}] after several minutes, with integrations showing compound **6** (please note that signals of minor intermediates overlap with resonances of **6**, influencing the integration).

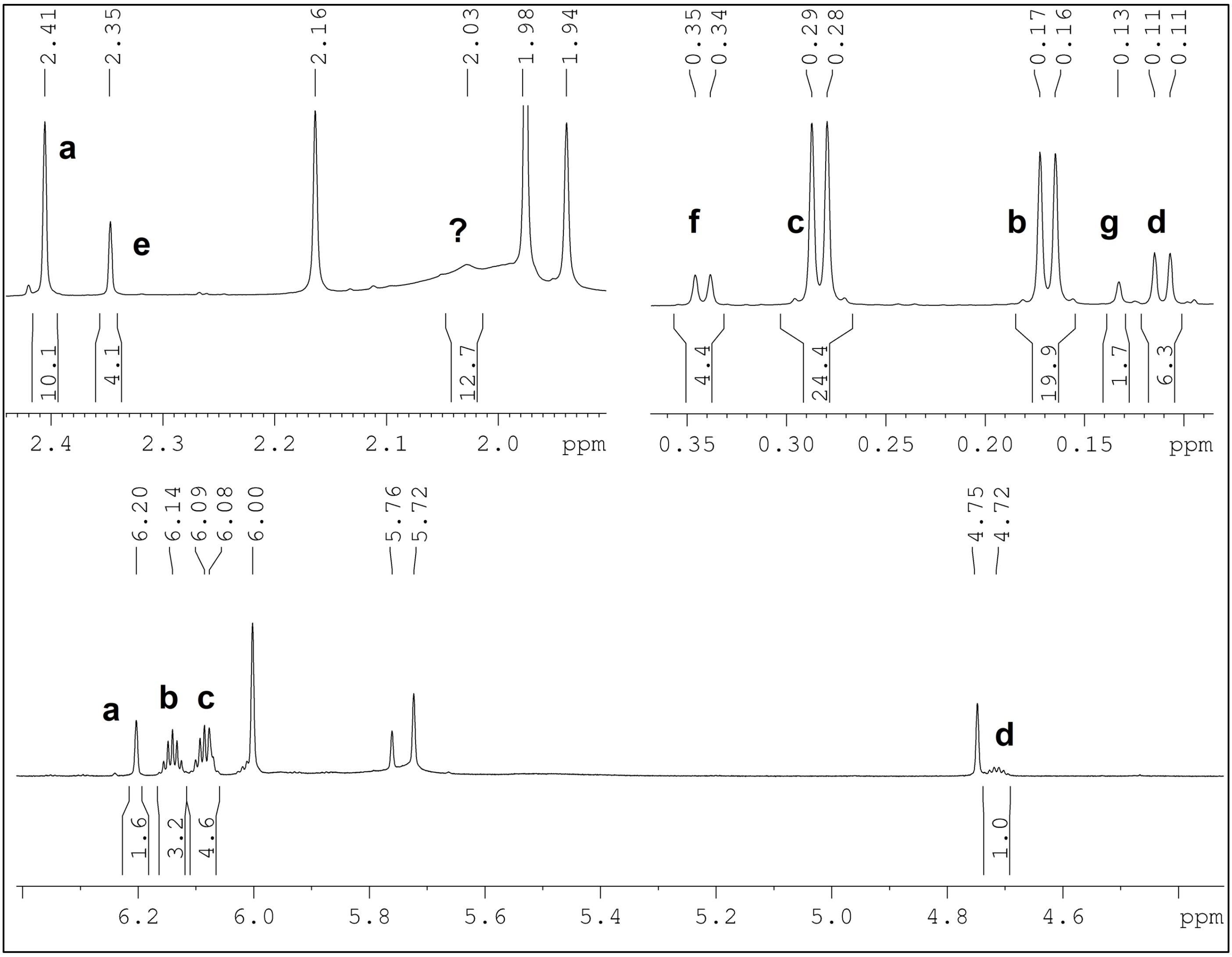

a) [Ce (Me$_2$pz)$_y${N(SiHMe$_2$)$_2$}$_x$] (y = 2 x = 2, or y = 3 x = 1)

b) Ce {N(SiHMe$_2$)$_2$} **e**) "Me$_2$pz" **?** = unknown intermediates

c) Ce {N(SiHMe$_2$)$_2$} **f**) "SiHMe$_2$"

d) HN(SiHMe$_2$)$_2$ **g**) "bpsa"

Figure S12.3 ^{1}H NMR ([D$_6$]benzene, 400 MHz, 300 K) spectrum of the reaction between [Ce(Me$_2$pz)$_4$]$_2$ (**1**) and [Li{N(SiHMe$_2$)$_2$}] after several minutes, with integrations referenced to ½ HN(SiHMe$_2$)$_2$, indicating the formation of **a-g** (**b** and **c** do not relate to [Li{N(SiHMe$_2$)$_2$)}] = δ = 4.66 ppm, and indicate the coordination of ({N(SiHMe$_2$)$_2$)}) to Ce as [Ce(Me$_2$py)$_x${N(SiHMe$_2$)$_2$)}$_y$] (x = 2, y = 2 (**4a**), or x = 3, y = 1 (**4b**)).

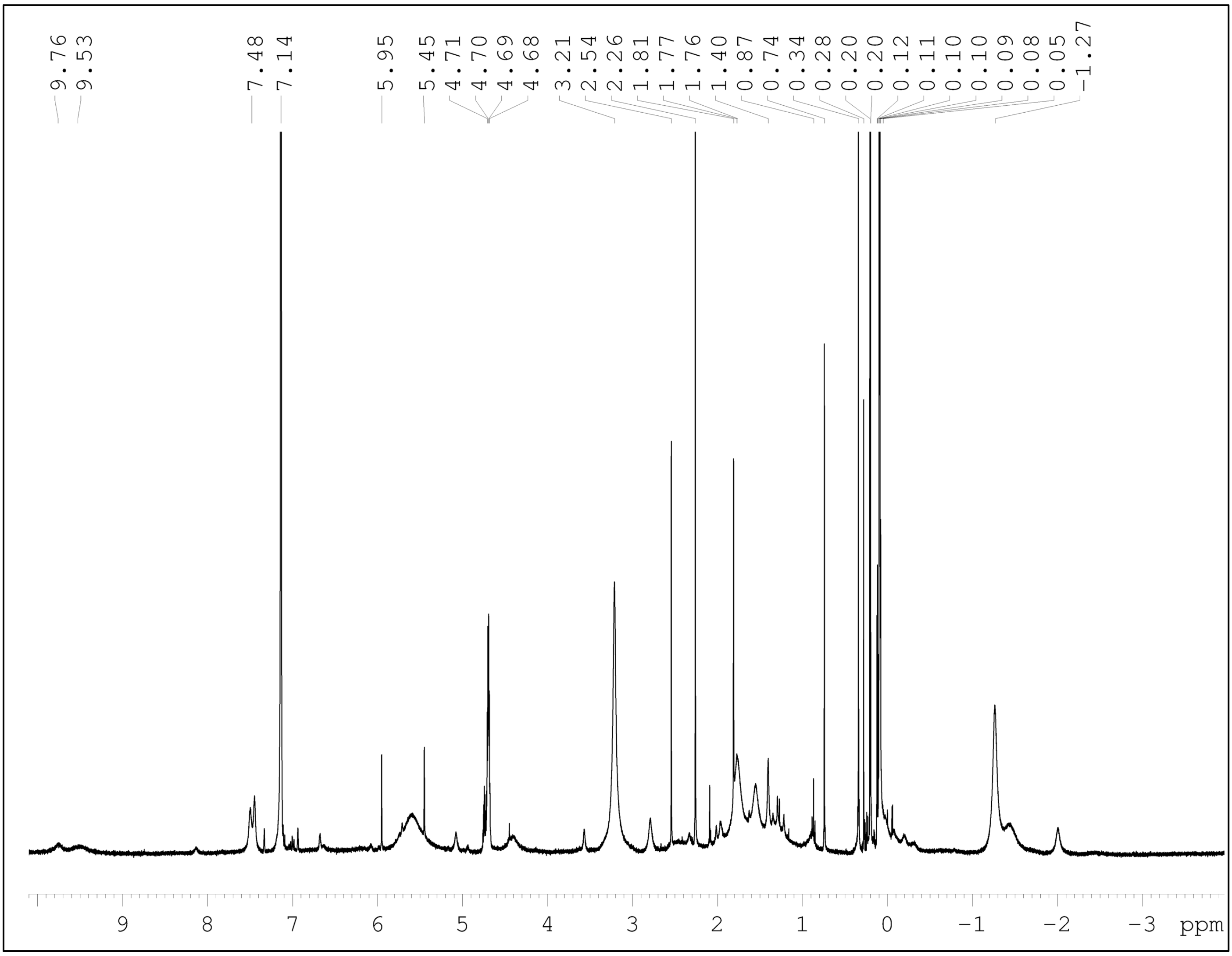

Figure S13. ^{1}H NMR ([D$_6$]benzene, 400 MHz, 300 K) spectrum of [Ce(Me$_2$pz)$_4$]$_2$ (**1**) and [Li{N(SiHMe$_2$)$_2$}] after two days, indicating the formation of a multitude of products (including paramagnetic cerium(III) complexes).

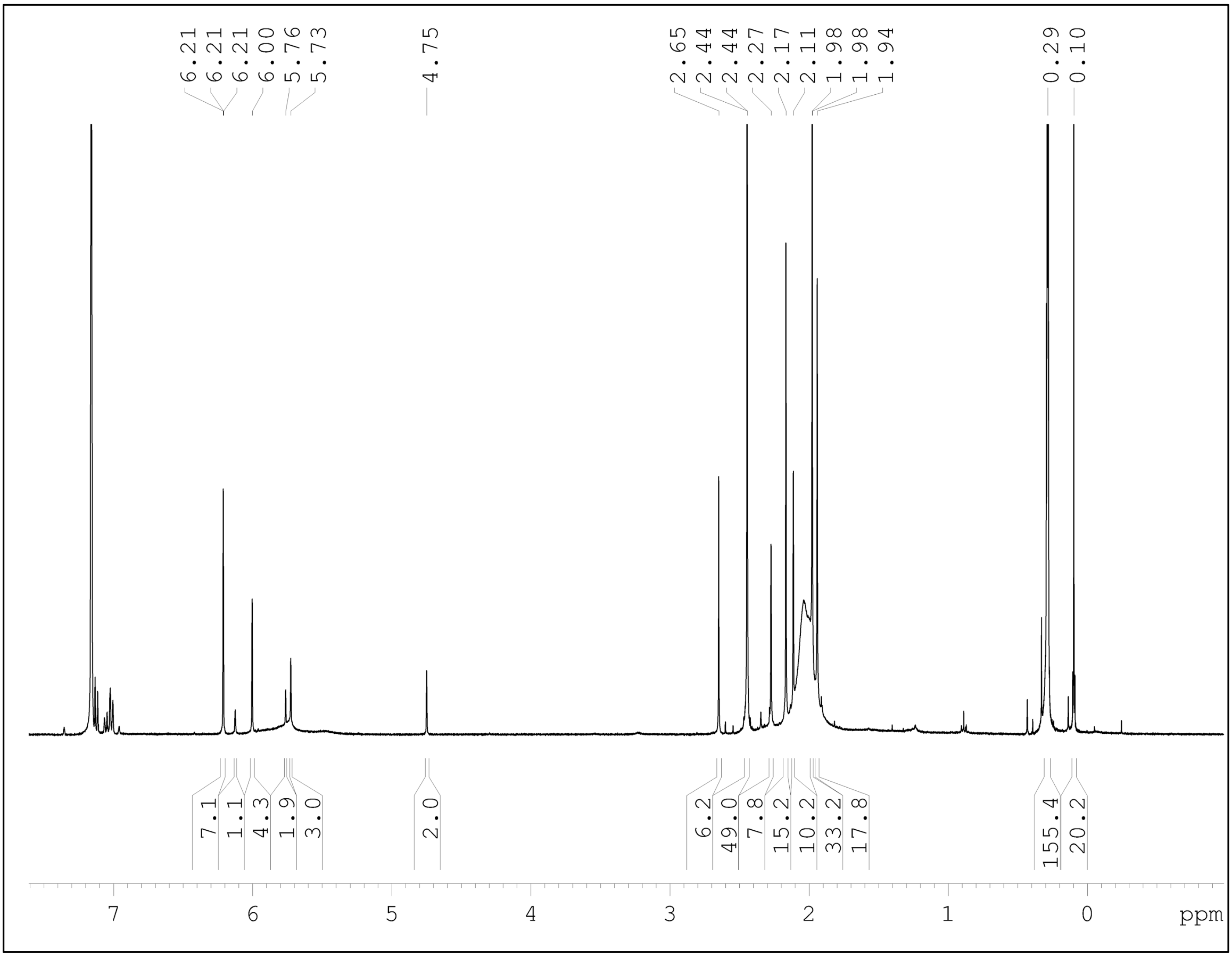

Figure S14.1 ¹H NMR ([D$_6$]benzene, 400 MHz, 300 K) spectrum of [Ce(Me$_2$pz)$_4$]$_2$ (**1**) and [Li{N(SiMe$_3$)$_2$}]. No changes in this reaction mixture occurred over several days (see Figure S11.2 and 11.3 for assignments).

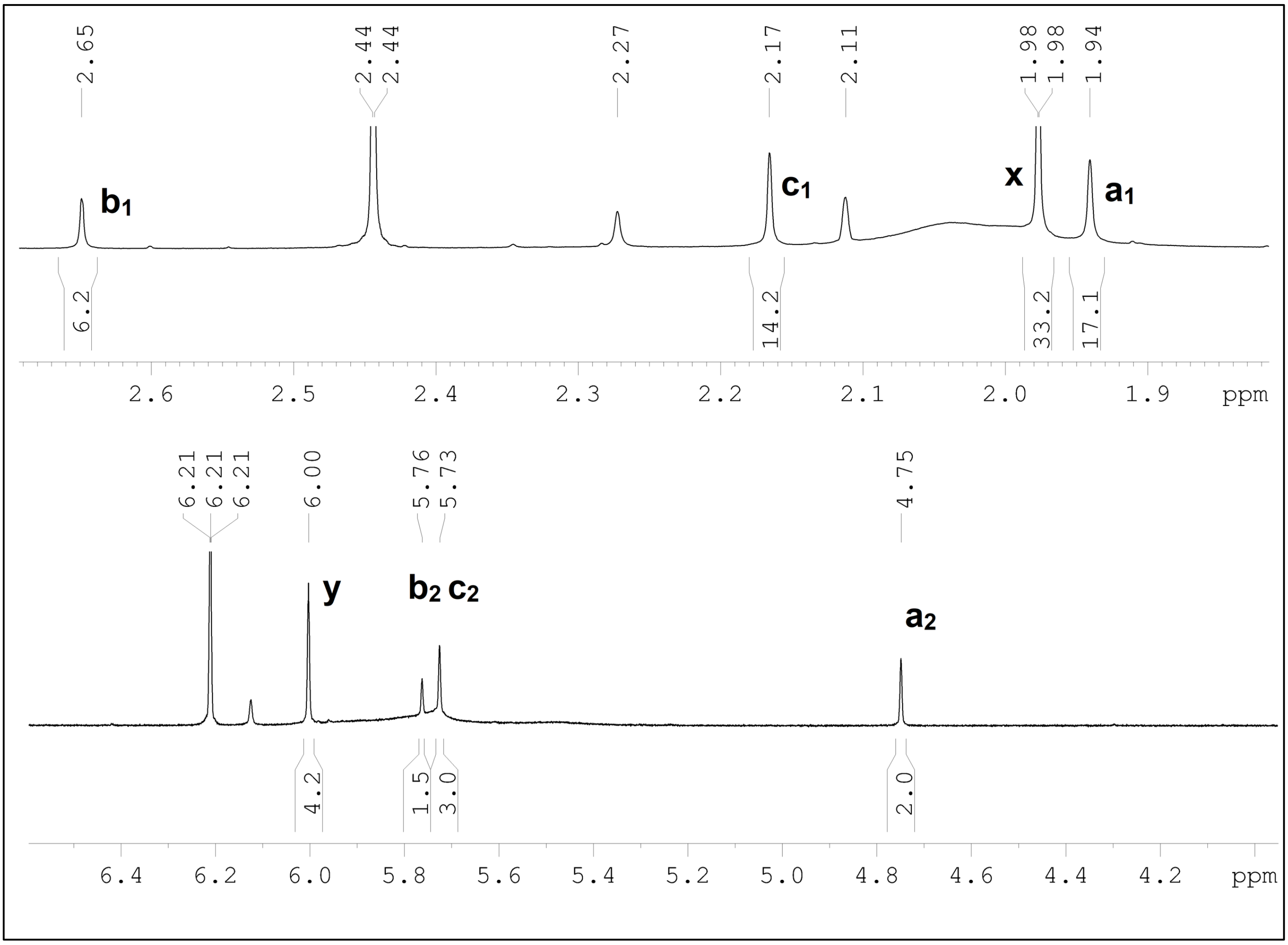

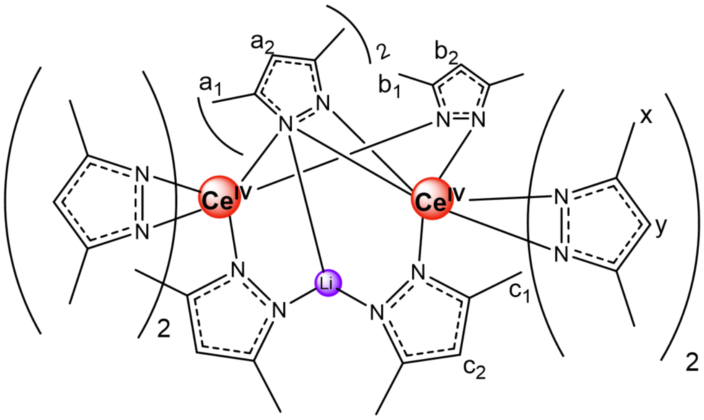

Figure S14.2 ^{1}H NMR ([D$_6$]benzene, 400 MHz, 300 K) spectrum of [Ce(Me$_2$pz)$_4$]$_2$ (**1**) and [Li{N(SiMe$_3$)$_2$}], showing the integrations for compound **6** (note: presence of other intermediate products underneath resonances influenced the integrations).

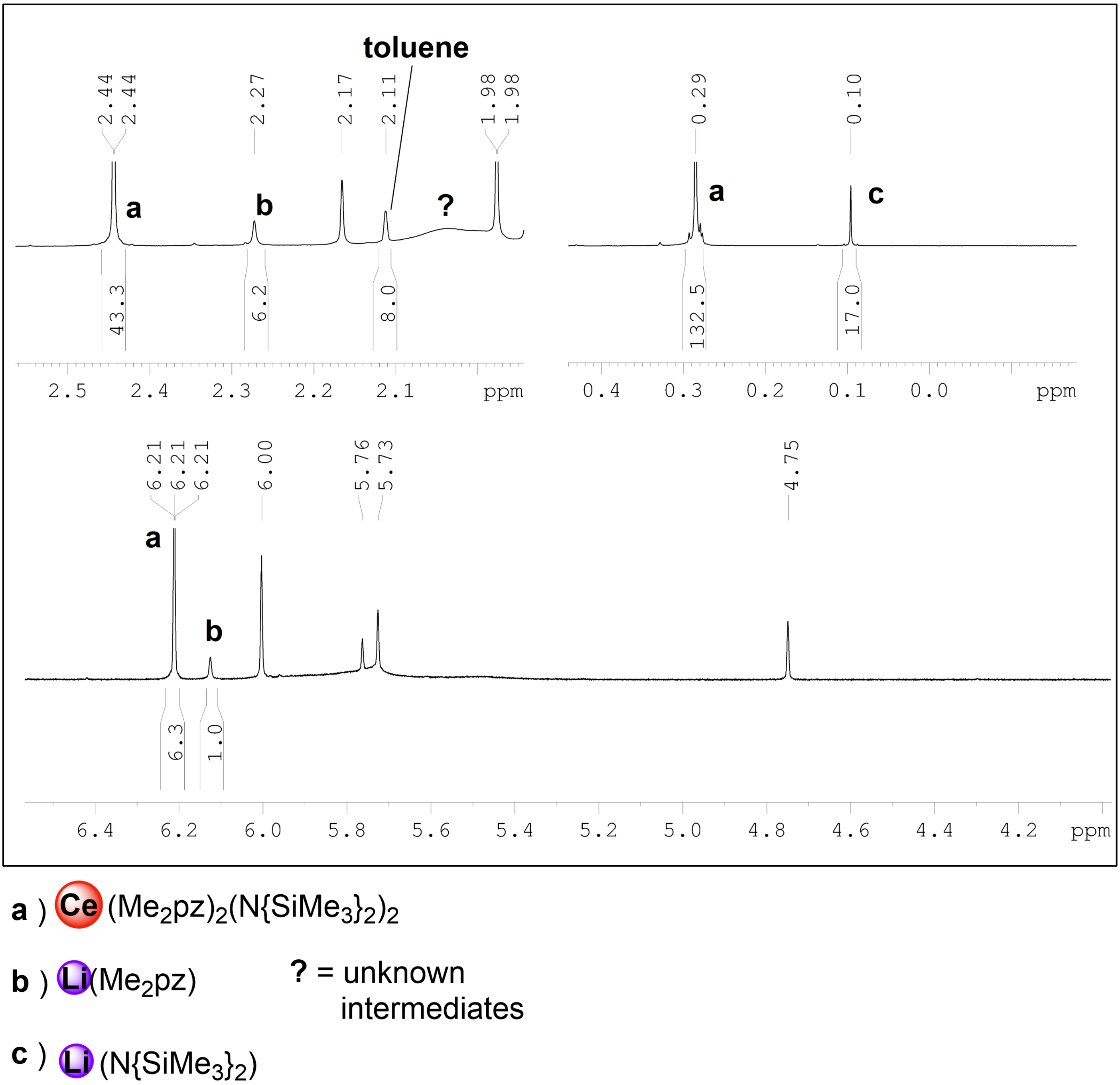

Figure S14.3 ^{1}H NMR ([D$_6$]benzene, 400 MHz, 300 K) spectrum of [Ce(Me$_2$pz)$_4$]$_2$ (**1**) and [Li{N(SiMe$_3$)$_2$}], showing the integrations for species **a**-**c**.

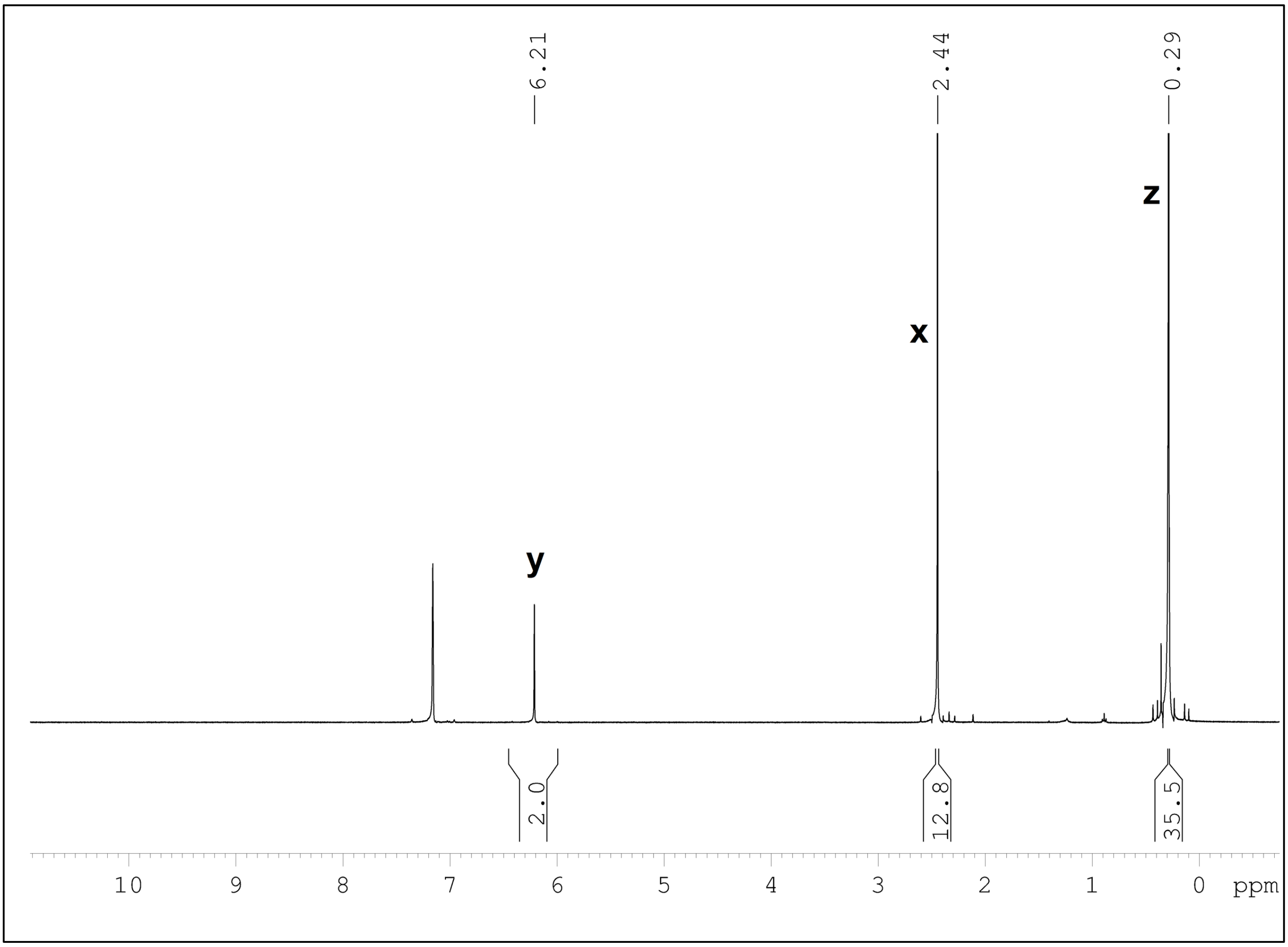

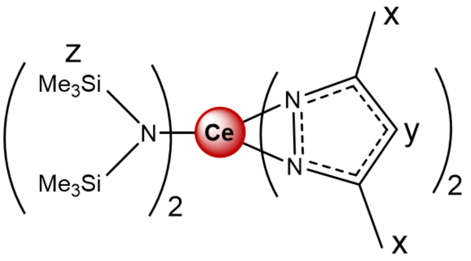

Figure S15. ^{1}H NMR ([D6]benzene, 400 MHz, 300 K) spectrum of [Ce(Me_2pz)$_2${N($SiMe_3$)$_2$}$_2$] (**7**).

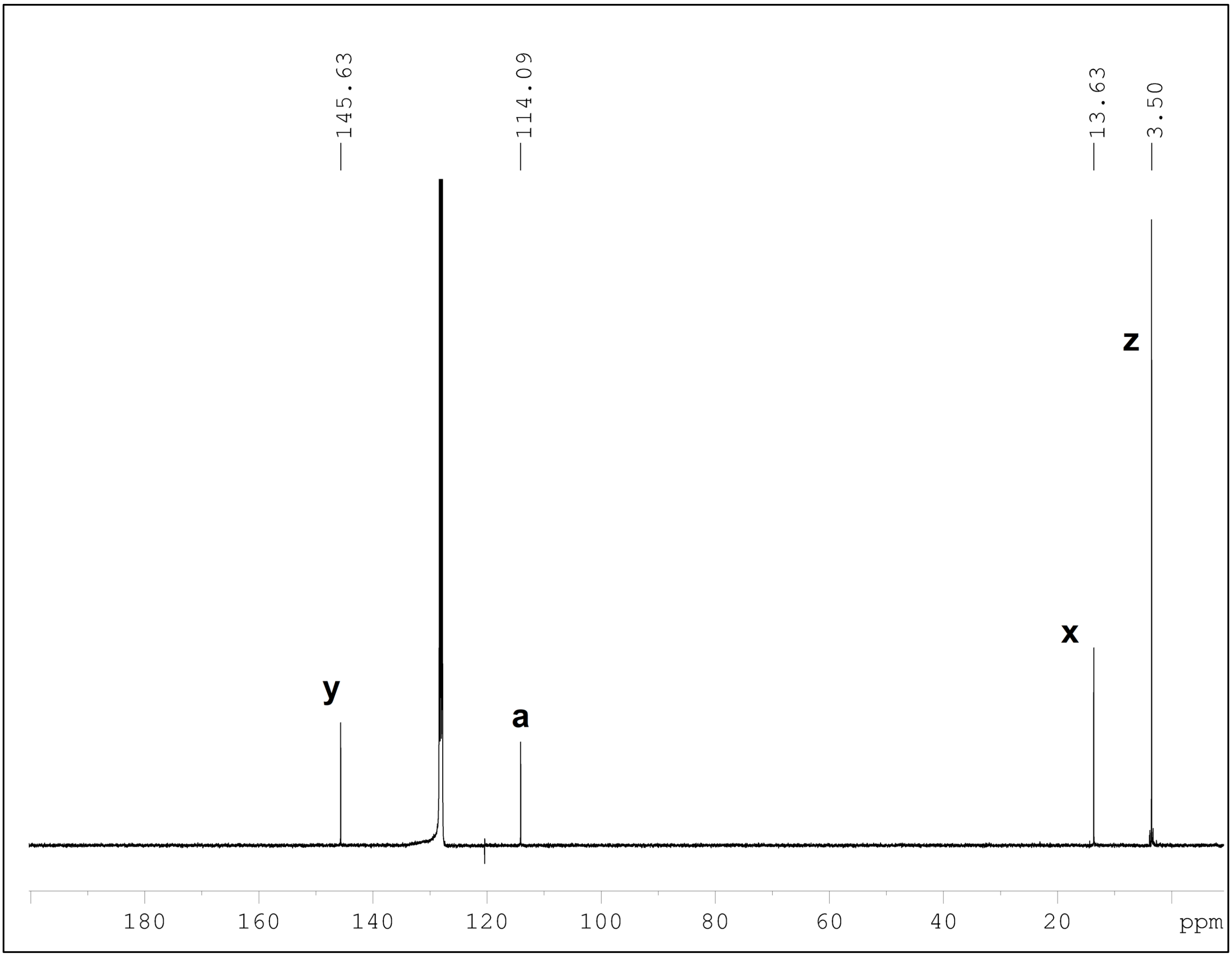

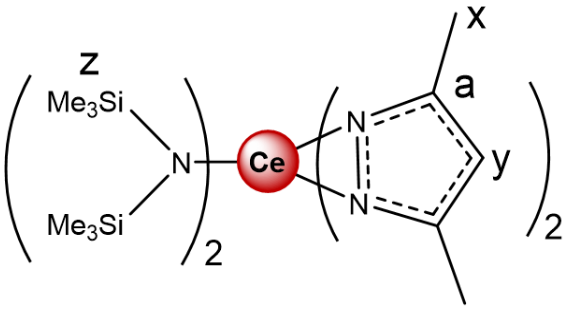

Figure S16. ^{13}C NMR ([D$_6$]benzene, 100 MHz, 300 K) spectrum of [Ce(Me$_2$pz)$_2${N(SiMe$_3$)$_2$}$_2$] (**7**).

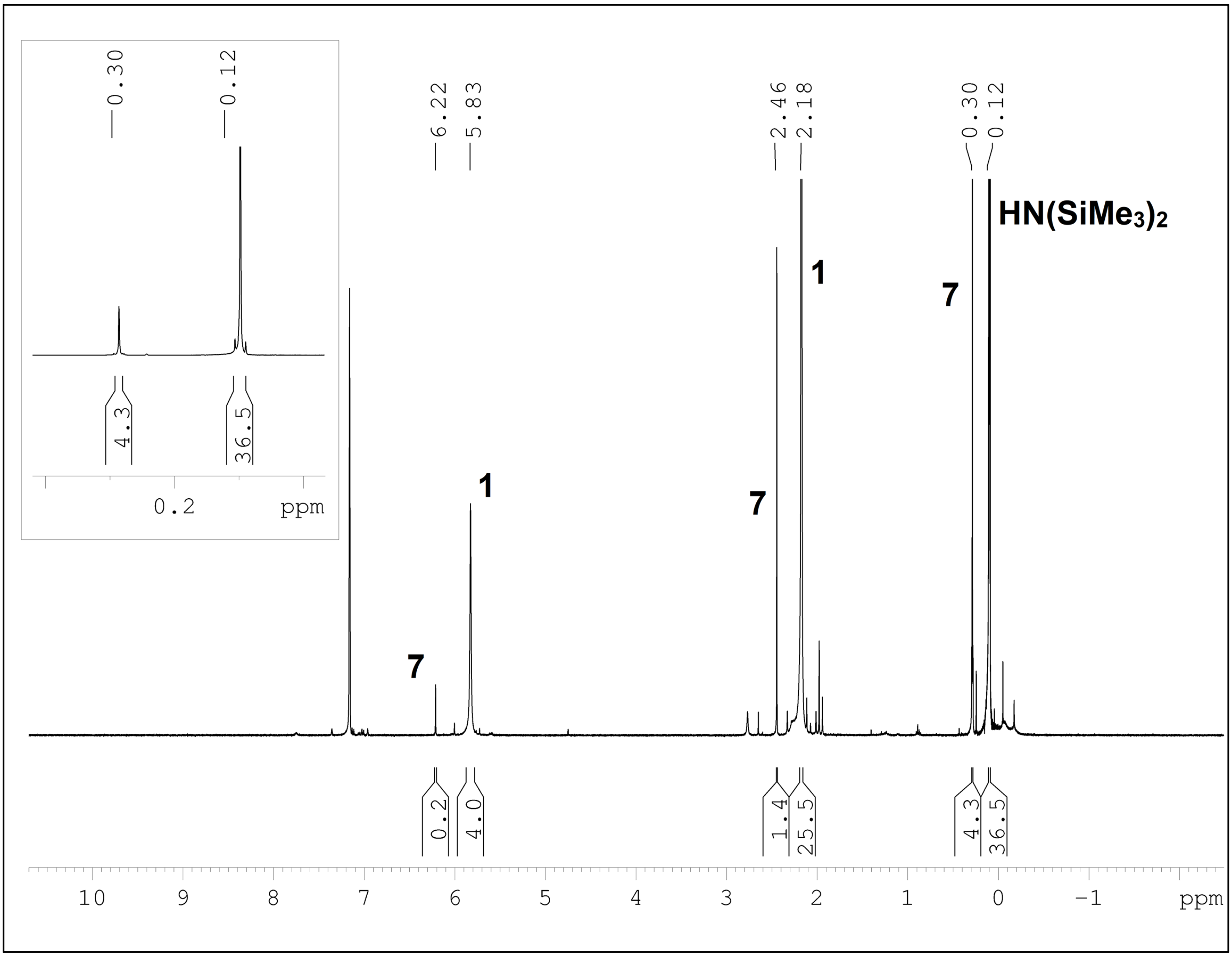

Figure S17. ^{1}H NMR ([D$_6$]benzene, 400 MHz, 300 K) spectrum of the reaction between exc. [Ce(Me$_2$pz)$_2${N(SiMe$_3$)$_2$}$_2$] (**7**) and Me$_2$pzH, giving [Ce(Me$_2$pz)$_4$]$_2$ (**1**), HN(SiMe$_3$)$_2$ and unreacted **7**.

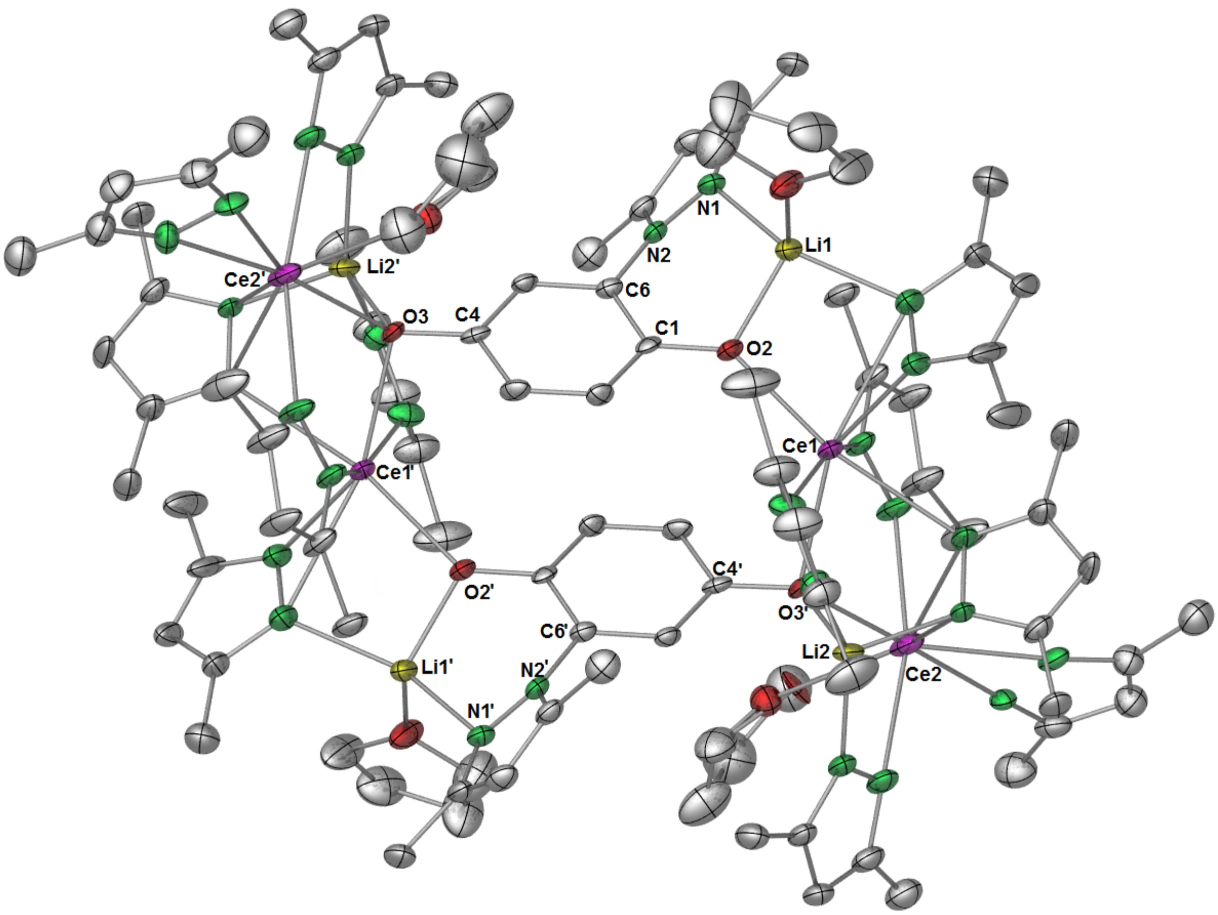

Figure S18. Crystal structure of $[Li_4Ce_4(Me_2pz)_{10}(Me_2pzH)_4(pzHq)_2]\cdot 2PhMe$ (**8**): ellipsoids shown at 30% probability and hydrogen atoms and lattice solvent removed for clarity. Selected bond lengths: Ce1–O2 2.323(5), Ce1–O3′ 2.409(5), Ce2–O3′ 2.443(5), Li1–O2 1.93(2), Li2–O3′ 2.09(2), Li1–N1 2.090(6), C6–N2 1.43(2), C6–C1 1.40(2), C1–O1 1.35(2), C4–O3 1.36(2).

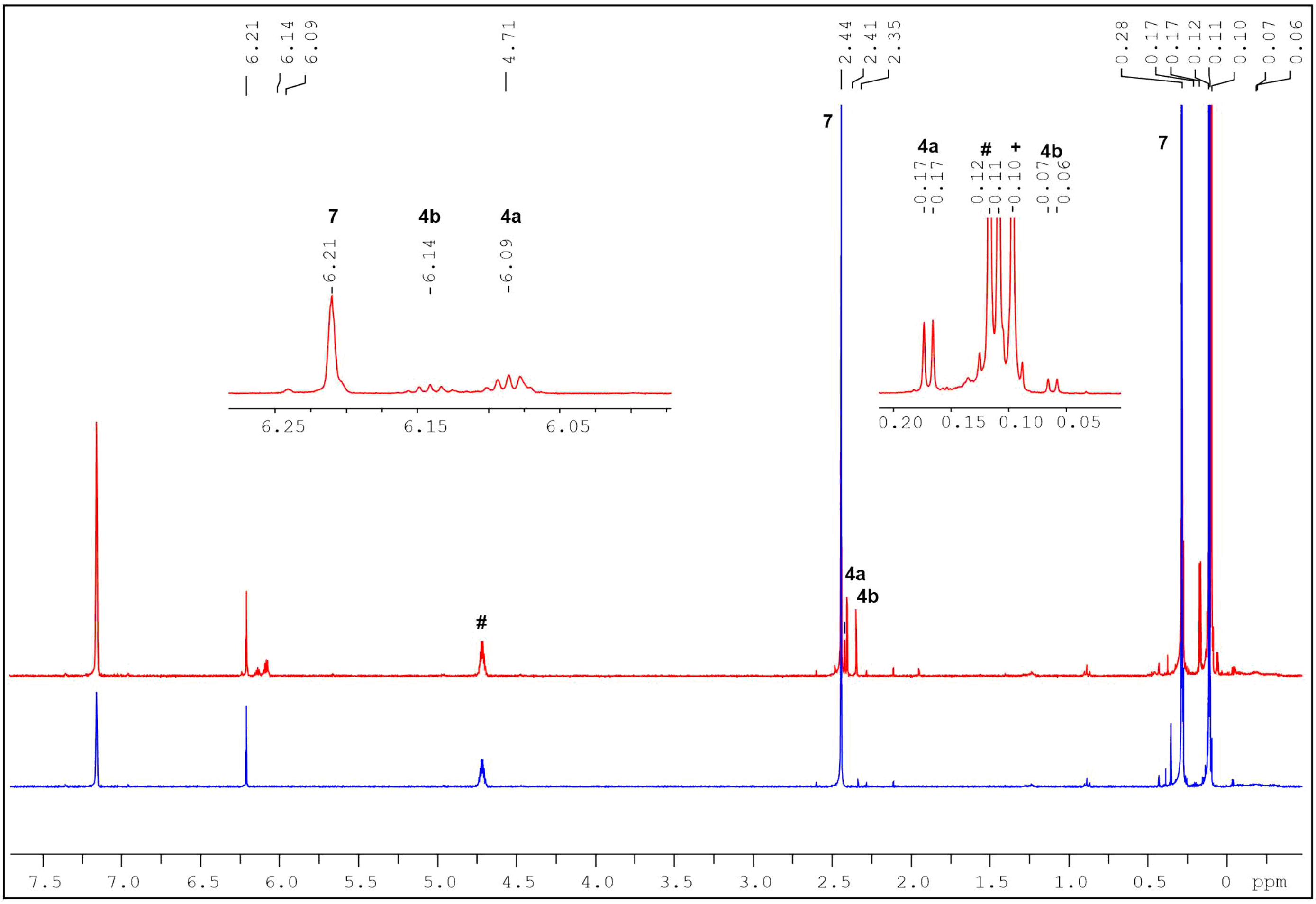

Figure S19. ^{1}H NMR ([D$_6$]benzene, 400 MHz) spectra of the reaction between [Ce(Me$_2$pz)$_2${N(SiMe$_3$)$_2$}$_2$] (**7**) and HN(SiHMe$_2$)$_2$, at ambient temperature upon addition (blue, bottom), and after 30 min at 60 °C (red, top), indicating the formation of **4a** and **4b** as well as decomposition.

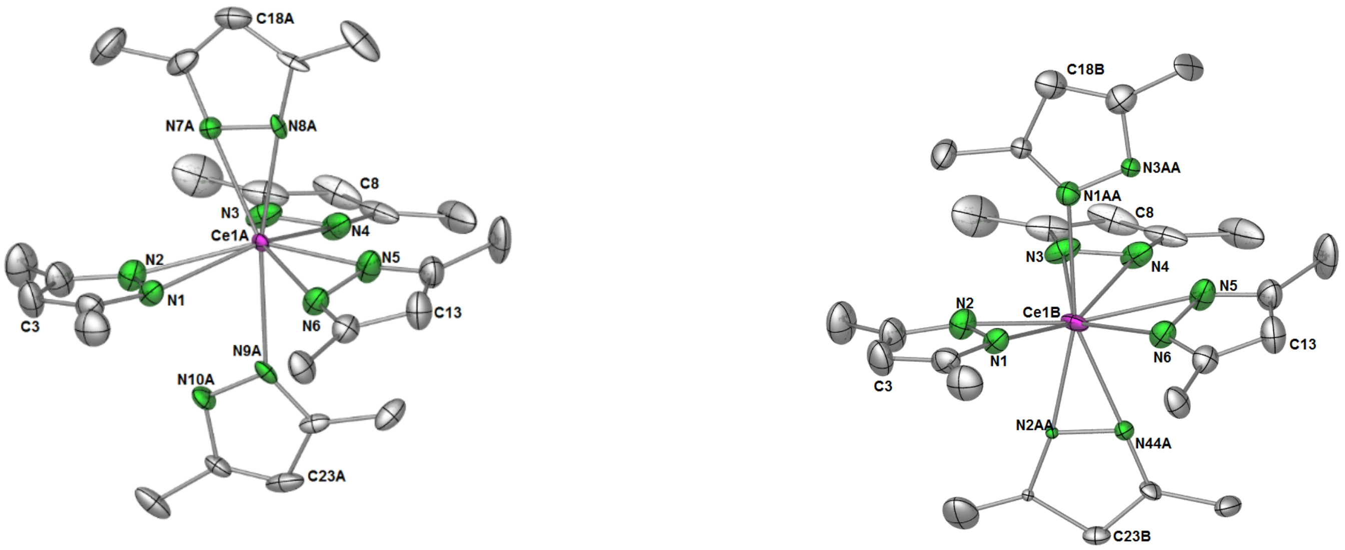

Figure S20. Connectivity of [Ce(Me_2pz)$_4$(Me_2pzH)] (**2b**). Ellipsoids shown at 50% probability, hydrogen atoms removed for clarity. Note: two molecules are present within the asymmetric unit. The positional disorder of both molecules is omitted for clarity.

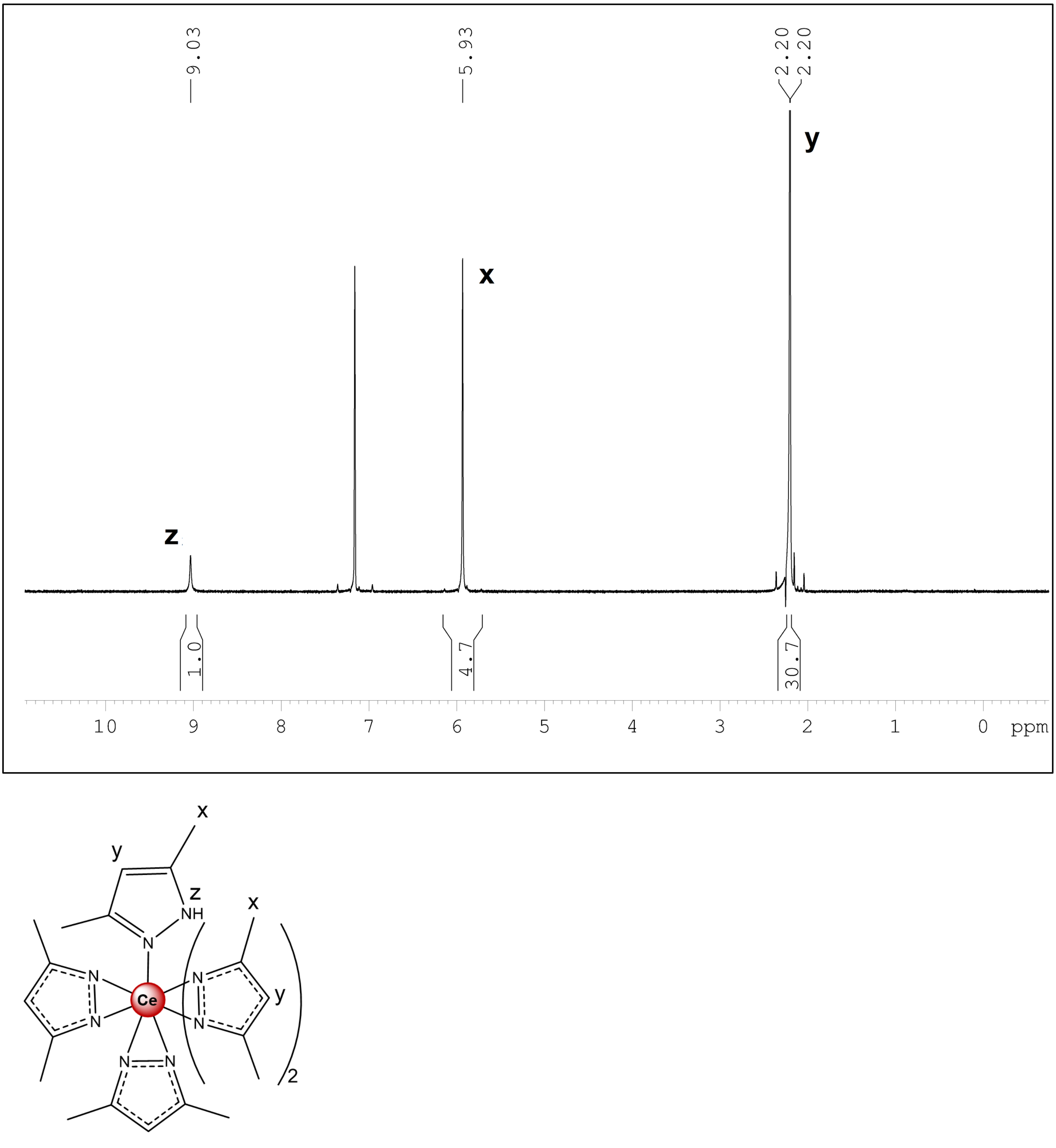

Figure S21. ^{1}H NMR ([D$_6$]benzene, 400 MHz, 300 K) spectrum of [Ce(Me$_2$pz)$_4$(Me$_2$pzH)] (**2b**).

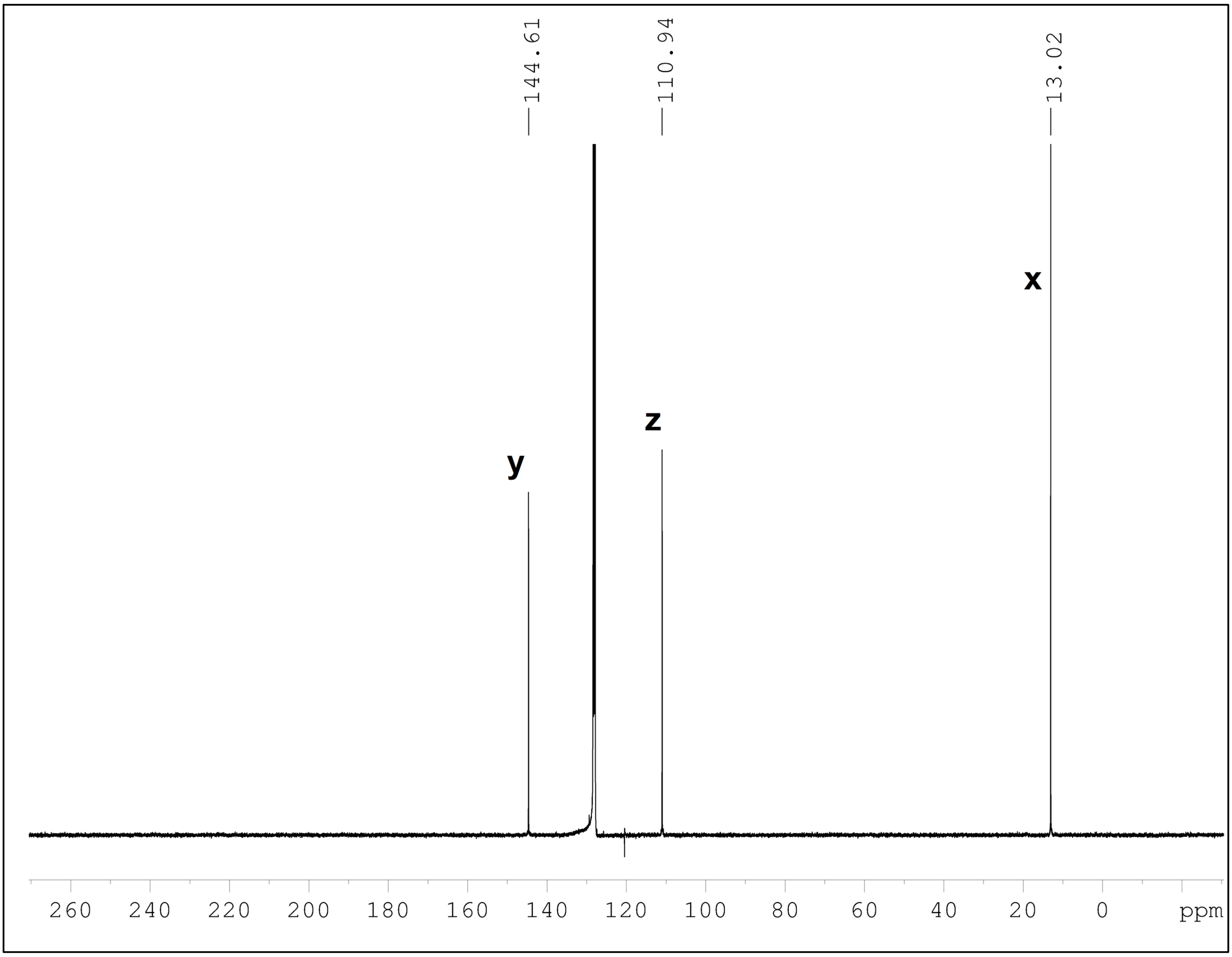

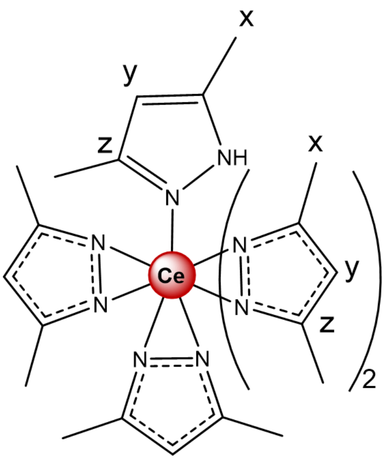

Figure S22. ^{13}C NMR ([D$_6$]benzene, 100 MHz, 300 K) spectrum of [Ce(Me$_2$pz)$_4$(Me$_2$pzH)] (**2b**).

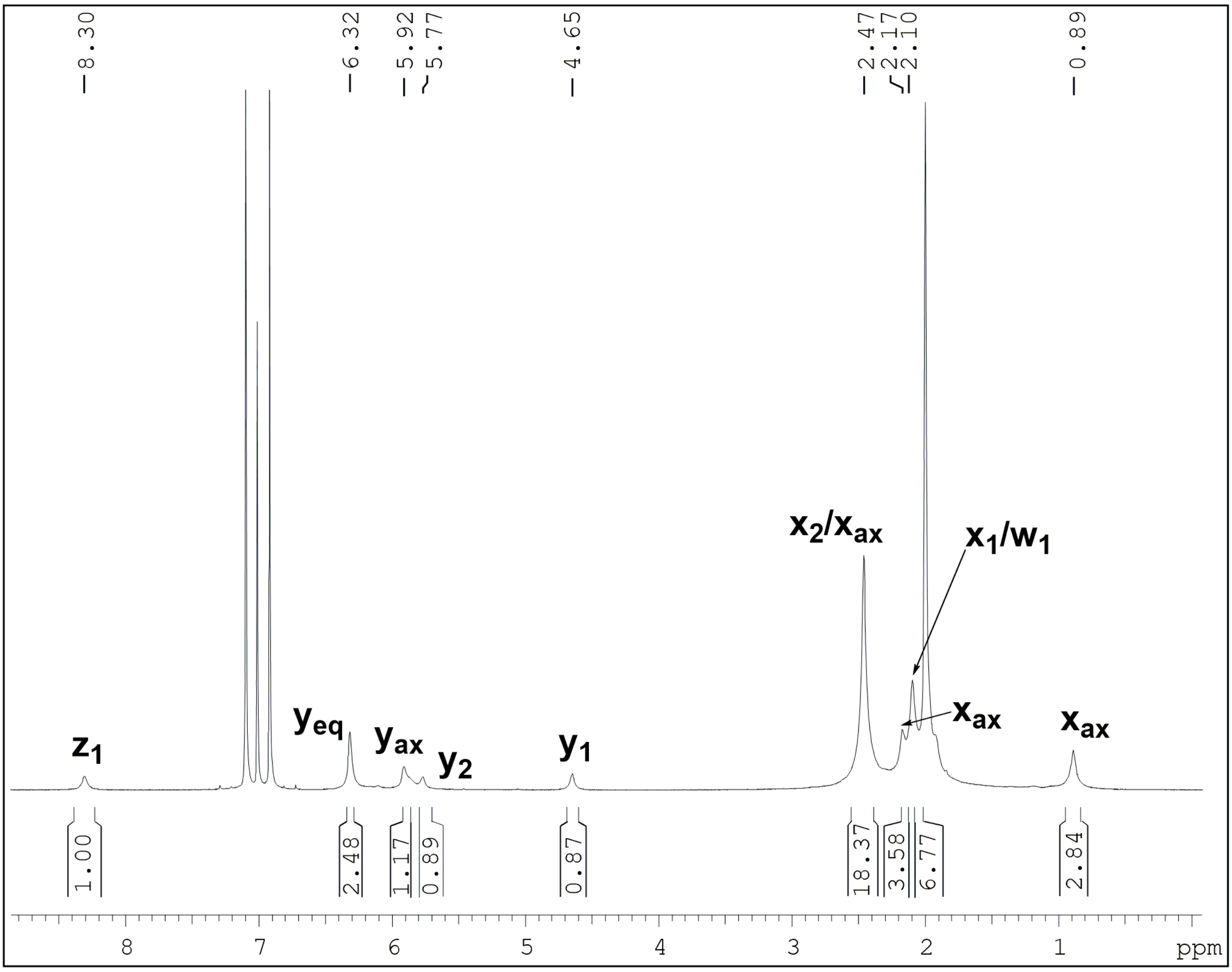

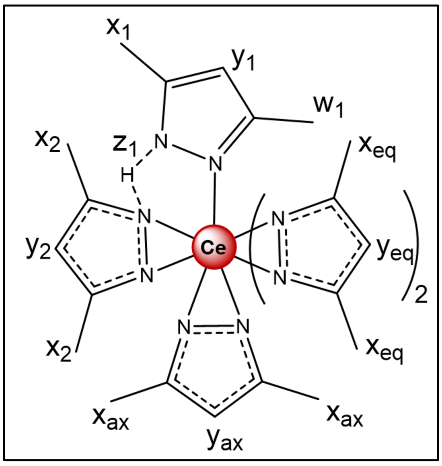

Figure S23. ^{1}H NMR ([D8]toluene, 400 MHz, 173 K) spectrum of [Ce($Me_2pz)_4(Me_2pzH$)] (**2b**).

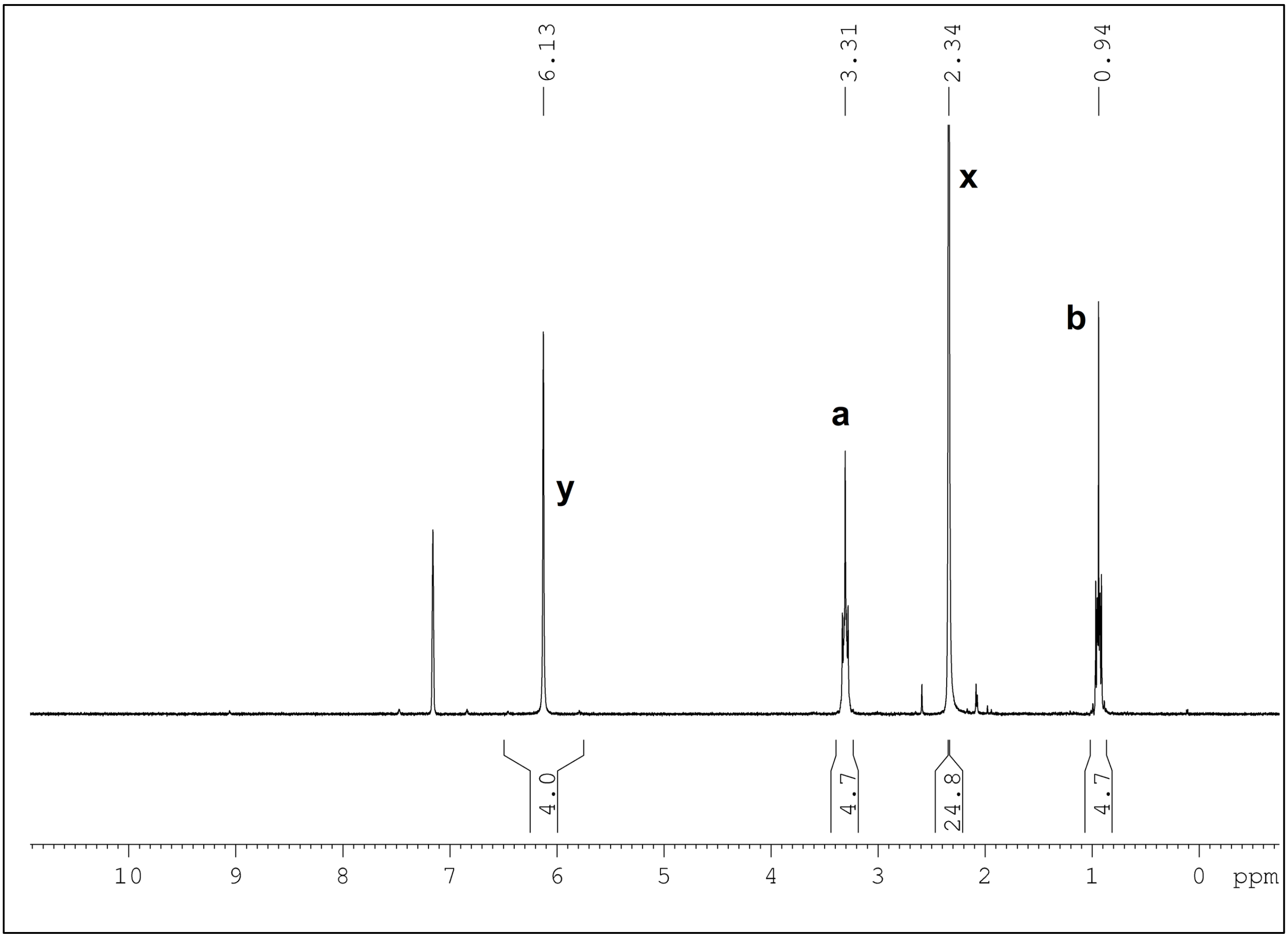

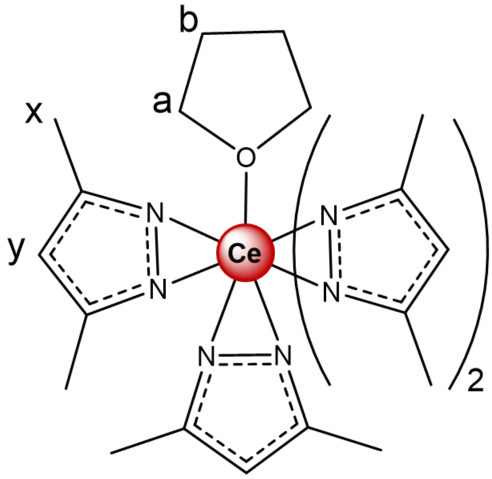

Figure S24. ^{1}H NMR ([D$_6$]benzene, 250 MHz, 300 K) spectrum of [Ce(Me$_2$pz)$_4$(thf)] (**2a**).

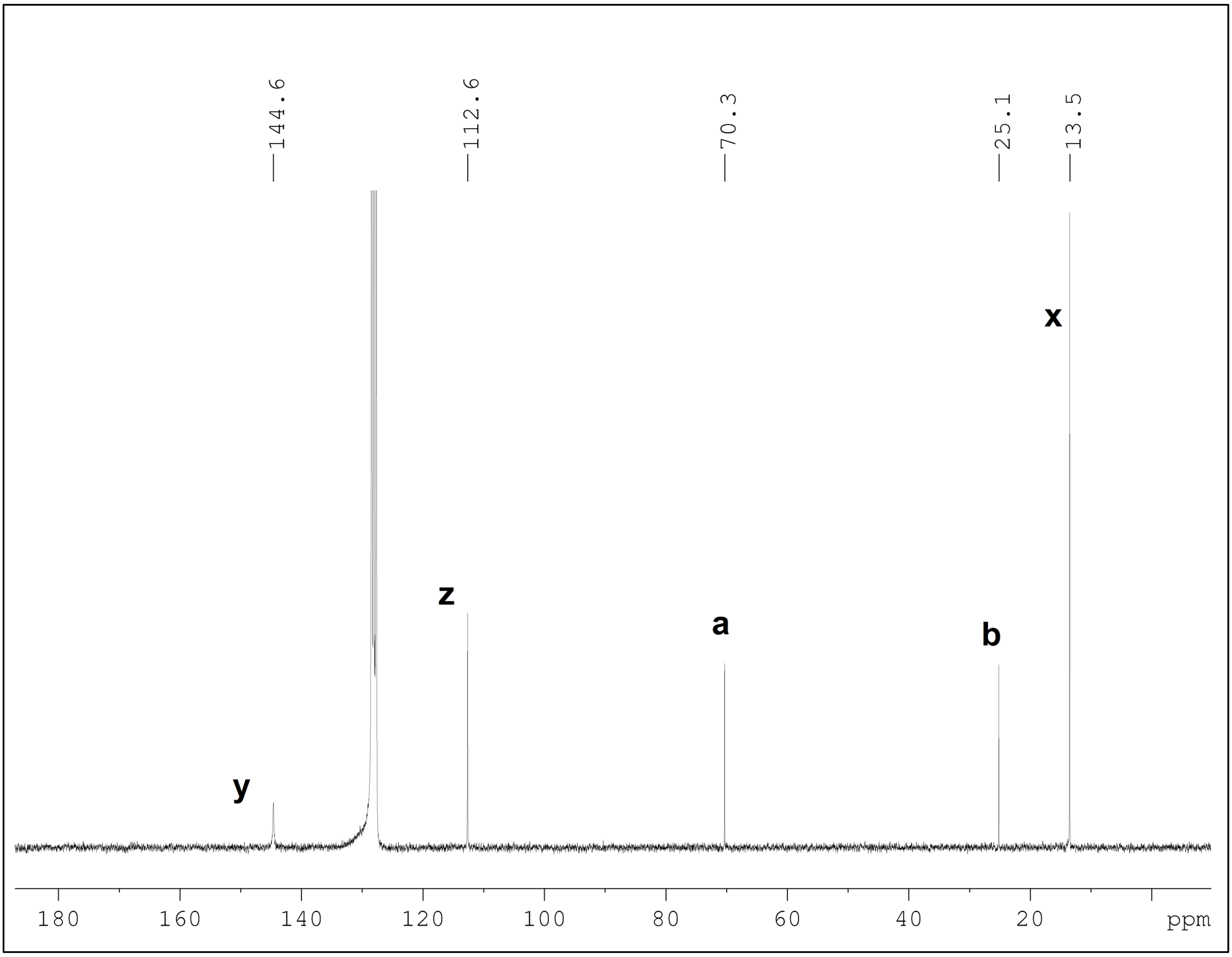

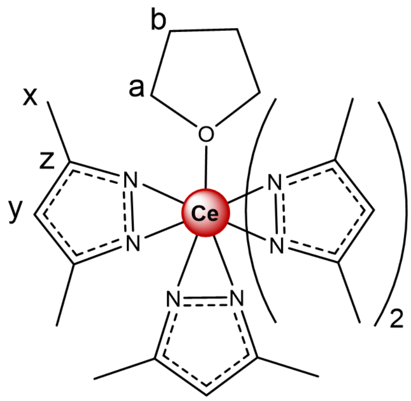

Figure S25. ^{13}C NMR ([D$_6$]benzene, 63 MHz, 300 K) spectrum of [Ce(Me$_2$pz)$_4$(thf)] (**2a**).

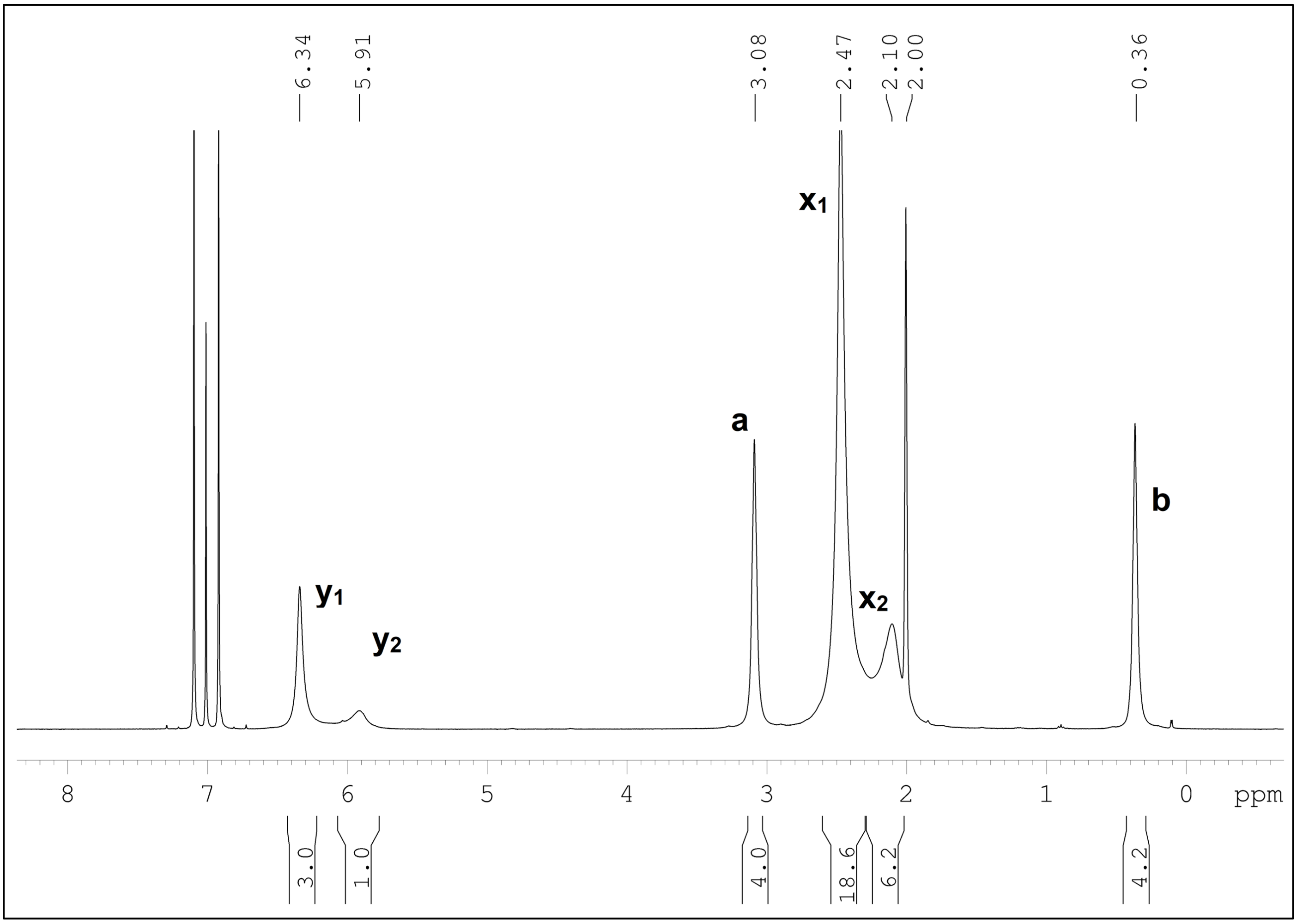

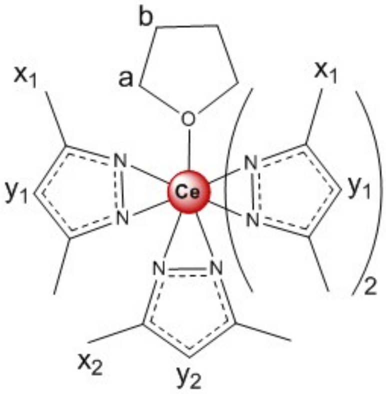

Figure S26. ^{1}H NMR ([D$_8$]toluene, 400 MHz, 173 K) spectrum of [Ce(Me$_2$pz)$_4$(thf)] (**2a**).

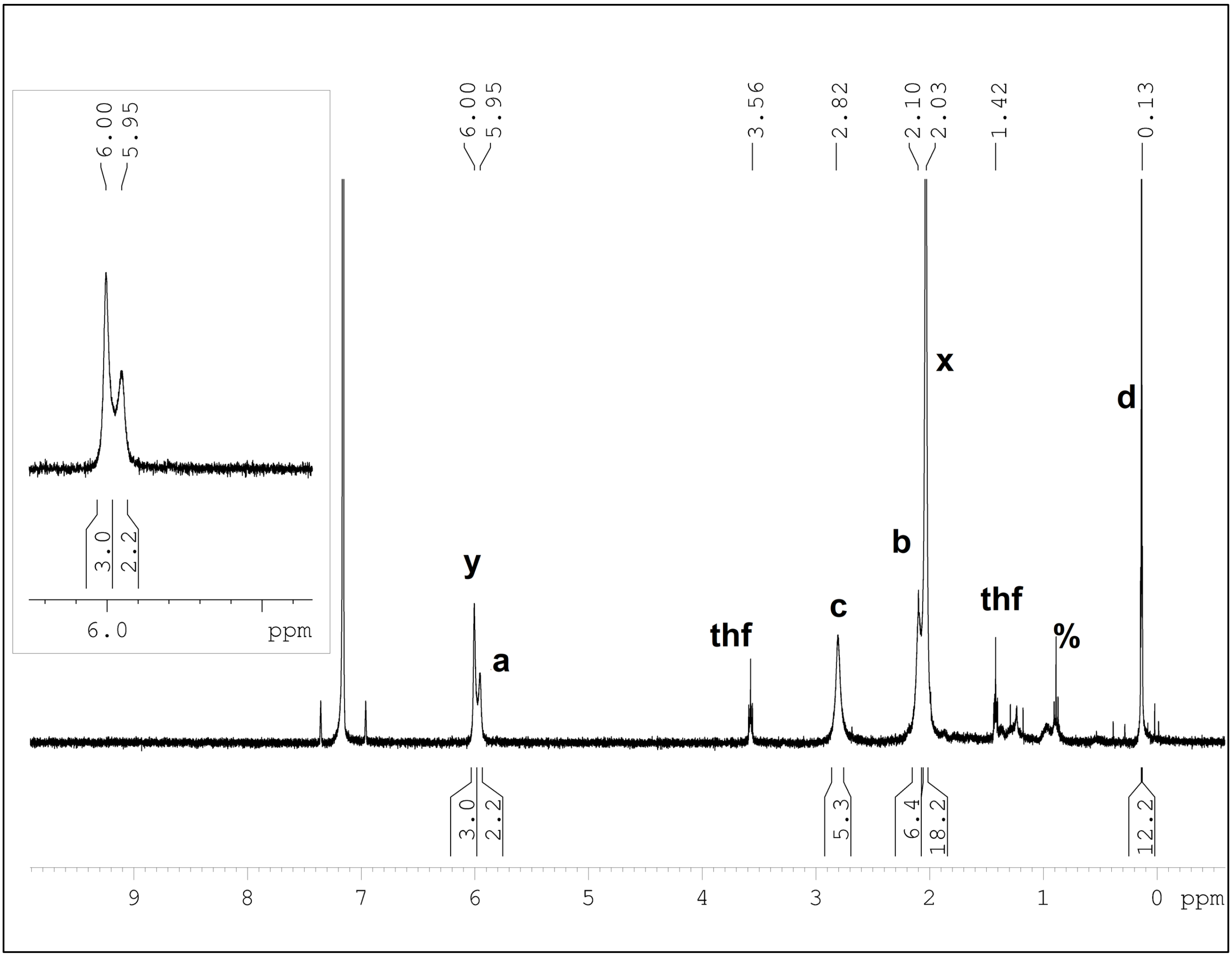

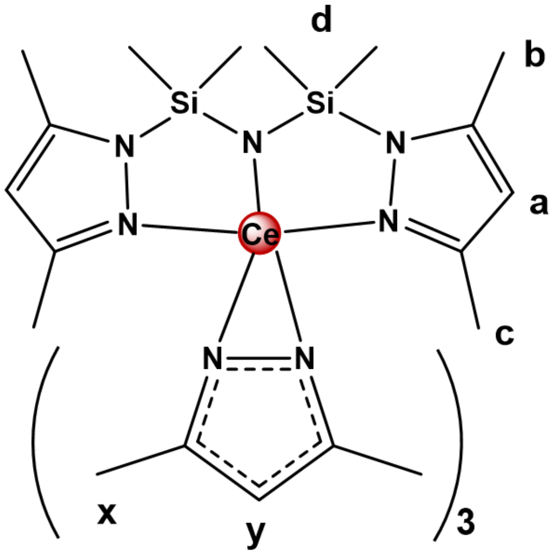

Figure S27. ^{1}H NMR ([D$_6$]benzene, 400 MHz, 300 K) spectrum of [Ce(Me$_2$pz)$_3$(bpsa)] (**3**). Trace solvent peaks came from glovebox atmosphere (thf and *n*-hexane %).

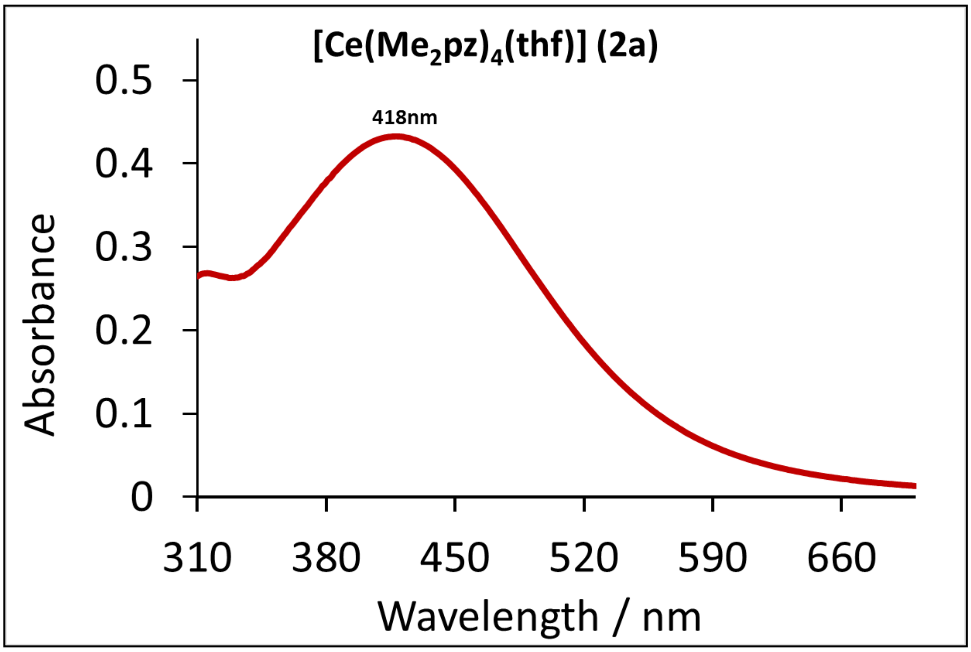

Figure S28. UV vis spectrum of [Ce(Me_2pz)$_4$(thf)] (**2a**) in toluene at ambient temperature.

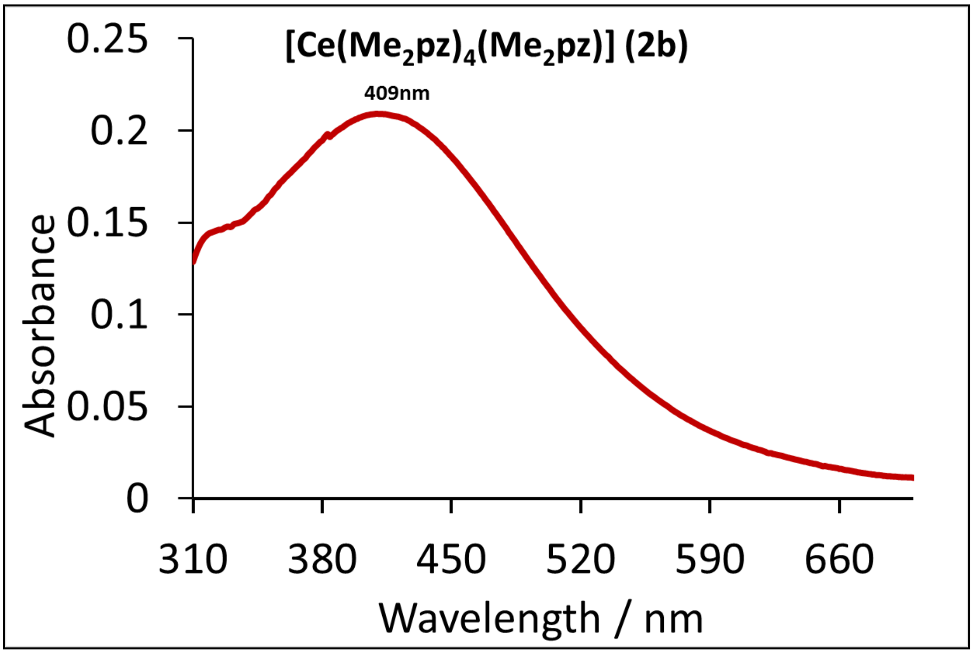

Figure S29. UV vis spectrum of [Ce(Me_2pz)$_4$(Me_2pzH)] (**2b**) in toluene at ambient temperature.

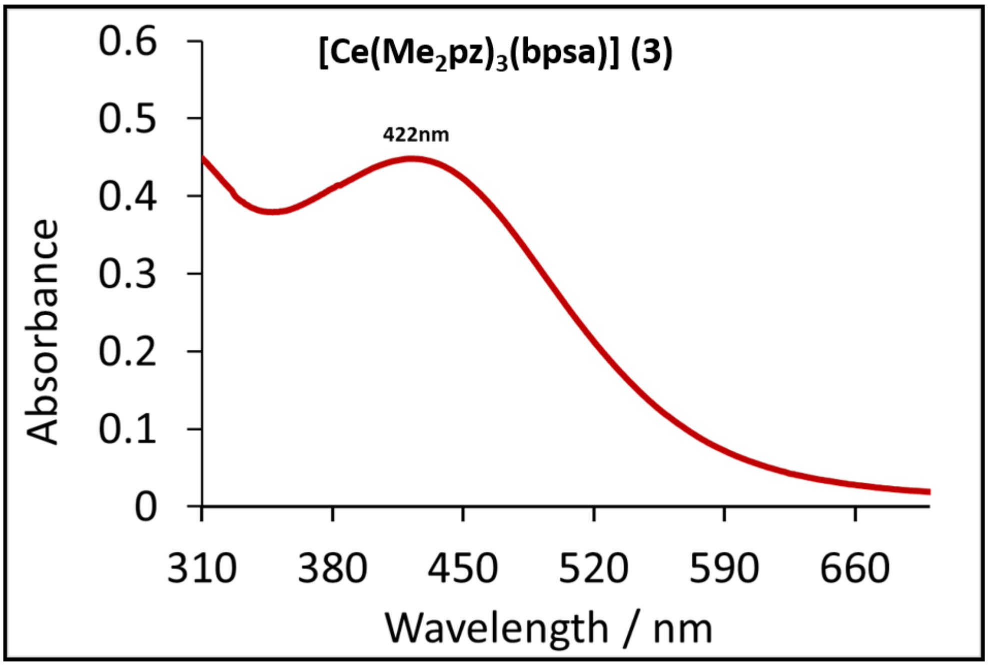

Figure S30. UV vis spectrum of [Ce(Me$_2$pz)$_3$(bpsa)] (**3**) in *n*-hexane at ambient temperature.

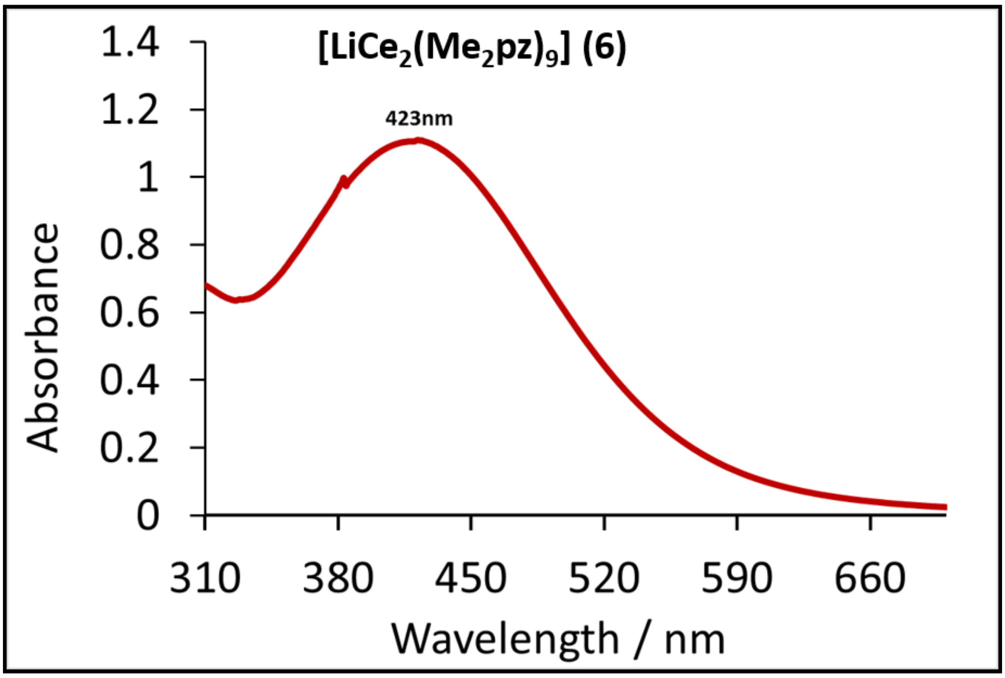

Figure S31. UV vis spectrum of [LiCe$_2$(Me$_2$pz)$_9$] (**6**) in toluene at ambient temperature.

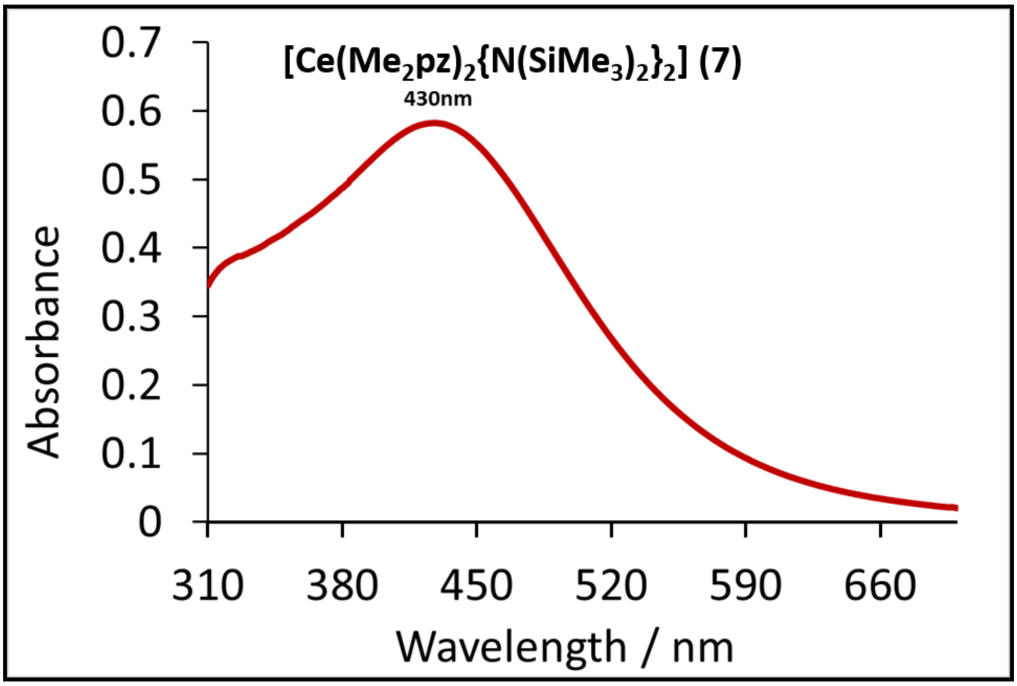

Figure S32. UV vis spectrum of [Ce(Me_2pz)$_2${N($SiMe_2H$)$_2$}$_2$] (**7**) in toluene at ambient temperature.

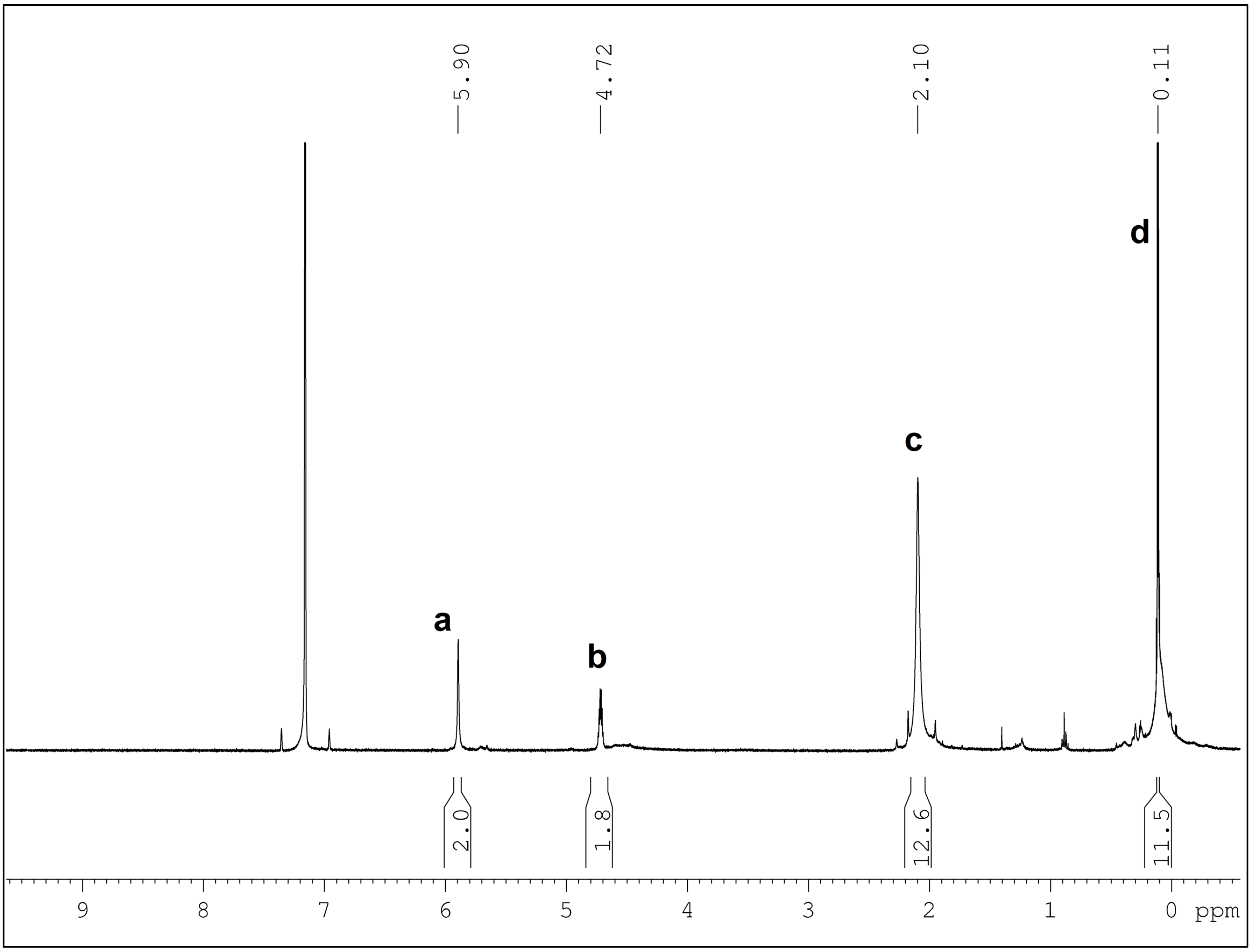

Figure S33. ^{1}H NMR ([D_6]benzene, 400 MHz, 300 K) spectrum of the reaction mixture between two equivalents of [Li(Me_2pz)] (**a** and **c**) and [Li{N($SiHMe_2$)$_2$}] (**b** and **d**) after heating at 115°C for two days showing minimal reactivity.

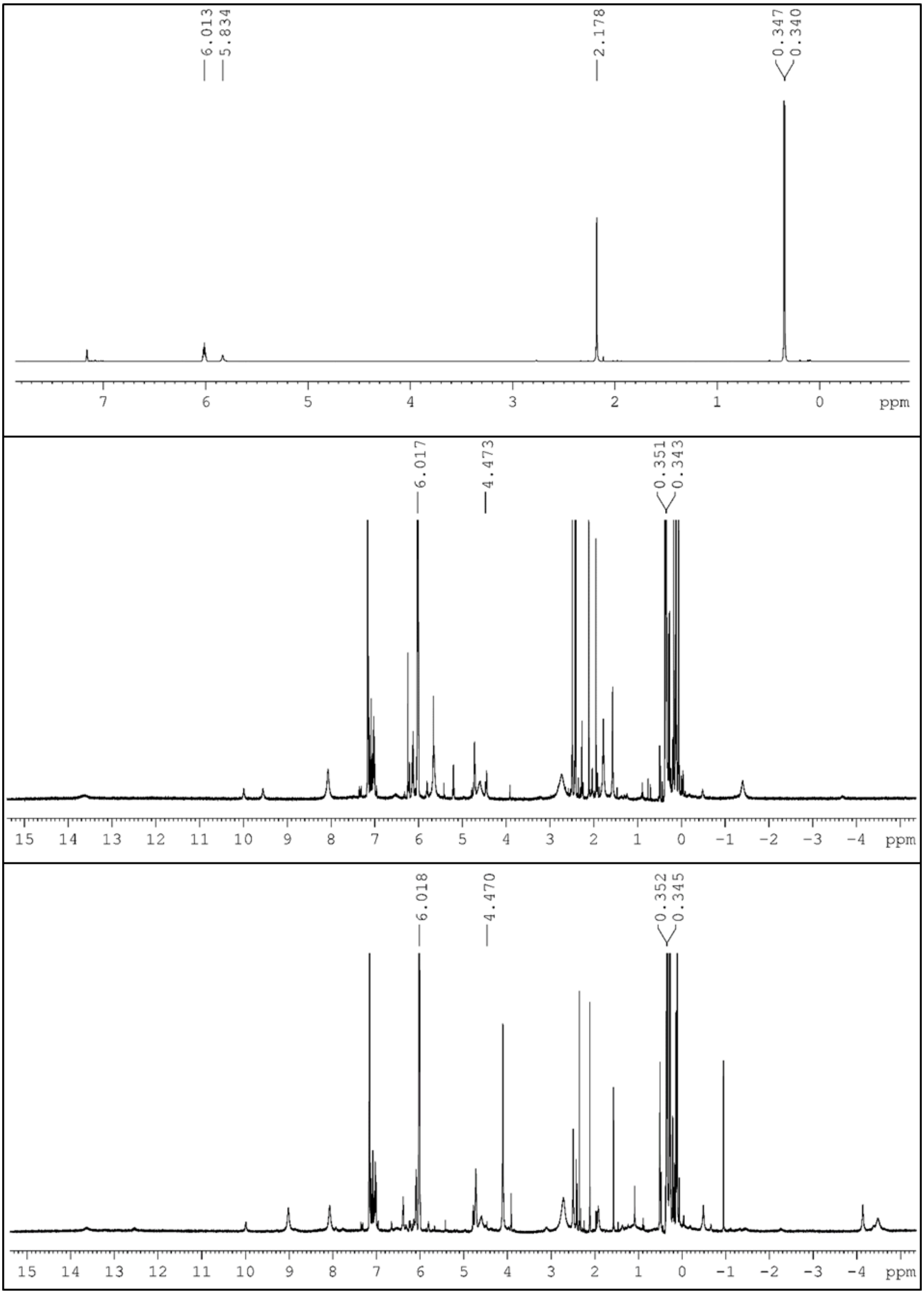

Figure S34. *Top*: ^{1}H NMR (C_6D_6, 400 MHz, 300 K) spectra of $[Ce(Me_2pz)_4]_2.{}^1/_2PhMe$ + $[Ce\{N(SiHMe_2)_2\}_4]$ after 15 minutes at room temperature. Middle: after one day. Bottom: after five days.

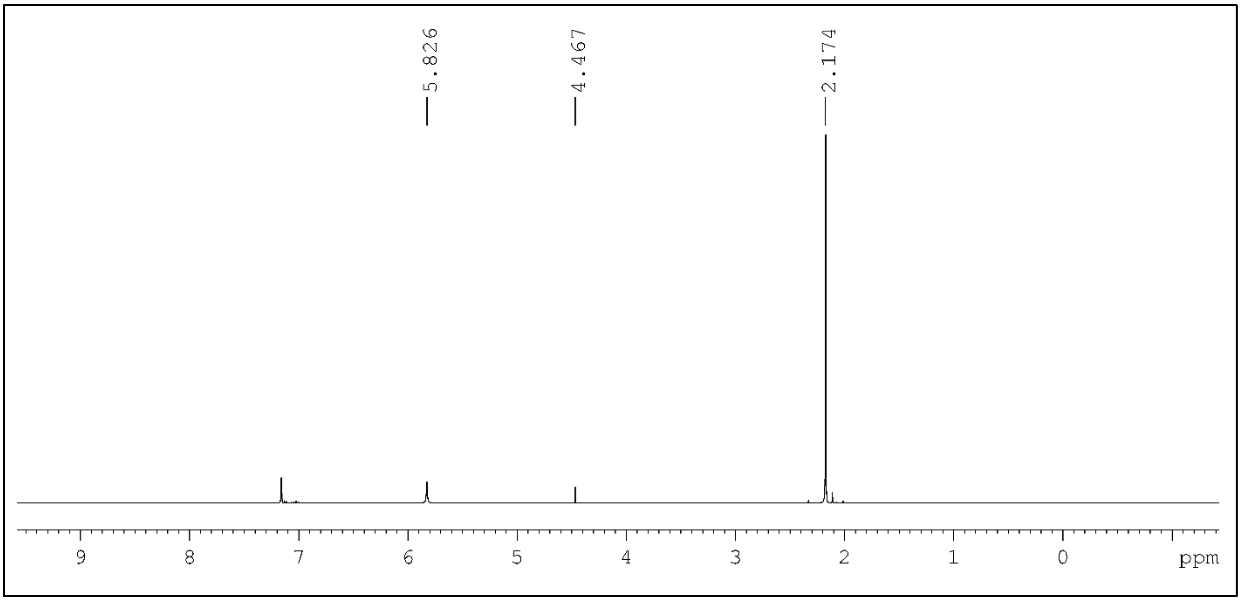

Figure S35. ^{1}H NMR (C_6D_6, 400 MHz, 300 K) spectrum of $[Ce(Me_2pz)_4]_2.{}^{1}/_{2}PhMe + H_2$.

Table S1. Crystallographic parameters for complexes **2a*-3**

**Identification code**	**2a***	**2b**	**2c**	**3**
**CCDC number**	1540352	1540356	1858038	1540354
**Empirical formula**	$C_{72}H_{108}Ce_3N_{24}O_3$	$C_{50}H_{70}Ce_2N_{20}$	$C_{70}H_{100}Ce_4N_{28}$	$C_{29}H_{47}CeN_{11}Si_2$
**Formula weight**	1778.18	1231.50	1894.26	746.07
**Crystal system**	triclinic	triclinic	triclinic	monoclinic
**Space group**	*P*-1	*P*-1	*P*-1	*C*2/*c*
**a/Å**	12.1247(19)	8.4522(7)	10.6603(3)	11.2391(12)
**b/Å**	17.863(3)	18.0527(14)	11.1917(3)	16.5453(17)
**c/Å**	19.384(3)	19.9637(16)	18.4557(5)	18.971(2)
**α/°**	90	100.946(3)	95.0271(12)	90
**β/°**	106.855(2)	93.742(4)	91.6842(12)	90.805(2)
**γ/°**	90	102.637(3)	113.0951(11)	90
**Volume/Å^3**	4017.9(11)	2900.1(4)	2012.74(10)	3527.4(6)
**Z**	2	1	1	4
**ρ_{calc}g cm^{-3}**	1.470	1.410	1.563	1.405
**μ/mm^{-1}**	1.730	1.601	2.276	1.395
**F(000)**	1812.0	1252.0	948	1536.0
**Data/restraints/parameters**	14164/30[d]/960	10194/188[d]/935	9230/0/519	4384/0/202
**Goodness-of-fit on F^2[a]**	1.054	1.086	1.049	1.028
**Final R indexes [I>=2σ (I)][b, c]**	R_1 = 0.0391, wR_2 = 0.0754	R_1 = 0.0518, wR_2 = 0.1335	R_1 = 0.0165, wR_2 = 0.0393	R_1 = 0.0307, wR_2 = 0.0630

[a]GOF = $[\Sigma w(F_0^2 - F_c^2)^2 / (n_0 - n_p)]^{1/2}$. [b]R1 = $\Sigma(||F_0| - |F_c||) / \Sigma|F_0|$, $F_0 > 4\sigma(F_0)$. [c]$wR_2 = \{\Sigma[w(F_0^2 - F_c^2)^2 / \Sigma[w(F_0^2)^2]\}^{1/2}$.[d] ISOR commands were used to remove NPD atoms generated from either poor crystal quality or complicated ligand disorder.

Table S2. Crystallographic parameters for complexes **5**-**8**

**Identification code**	**5**	**6**	**7**	**8**
**CCDC number**	1858039	1540351	1540350	1540355
**Empirical formula**	$C_{30}H_{72}CeLi_2N_7O_2Si_6$	$C_{52}H_{71}Ce_2LiN_{18}$	$C_{22}H_{50}CeN_6Si_4$	$C_{50.75}H_{70}Ce_2Li_2N_{14}O_4$
**Formula weight**	885.49	1235.44	651.16	1234.32
**Crystal system**	monoclinic	monoclinic	monoclinic	monoclinic
**Space group**	$P2_1/c$	*C*2/*c*	*C*2/*c*	*C*2/*c*
**a/Å**	15.0690(11)	28.146(7)	18.3765(18)	28.089(2)
**b/Å**	16.5305(12)	13.749(3)	14.1163(18)	17.547(2)
**c/Å**	19.2223(14)	15.636(4)	12.7881(14)	27.882(3)
**α/°**	90.00	90	90	90
**β/°**	95.9636(12)	107.712(5)	99.308(2)	105.919(2)
**γ/°**	90.00	90	90	90
**Volume/Å^3**	4762.3(6)	5764(3)	3273.7(6)	13215(3)
**Z**	4	4	4	8
**ρ_{calc}g cm^{-3}**	1.235	1.424	1.321	1.241
**μ/mm^{-1}**	1.139	1.610	1.557	1.407
**F(000)**	1860.0	2512.0	1352.0	5012.0
**Data/restraints/parameters**	11777/0/468	7232/19[d]/349	4799/0/158	11606/133[d]/758
**Goodness-of-fit on F^2[a]**	1.046	1.045	1.051	1.076
**Final R indexes [I>=2σ (I)][b,c]**	R_1 = 0.0267, wR_2 = 0.0646	R_1 = 0.0256, wR_2 = 0.0570	R_1 = 0.0265, wR_2 = 0.0532	R_1 = 0.0949, wR_2 = 0.2592

[a]GOF = $[\Sigma w(F_0^2 - F_c^2)^2 / (n_0 - n_p)]^{1/2}$. [b]R1 = $\Sigma(||F_0| - |F_c||) / \Sigma|F_0|$, $F_0 > 4\sigma(F_0)$. [c]$wR_2 = \{\Sigma[w(F_0^2 - F_c^2)^2 / \Sigma[w(F_0^2)^2]]\}^{1/2}$.[d] ISOR commands were used to remove NPD atoms.
